# Global-scale computational analysis of genomic sequences reveals the recombination pattern and coevolution dynamics of cereal-infecting geminiviruses

**DOI:** 10.1038/srep08153

**Published:** 2015-01-30

**Authors:** Beilei Wu, Xiaonan Shang, Jörg Schubert, Antje Habekuß, Santiago F. Elena, Xifeng Wang

**Affiliations:** 1 State Key Laboratory for Biology of Plant Diseases and Insect Pests, Institute of Plant Protection, Chinese Academy of Agricultural Sciences, Beijing 100193, China; 2 Institute for Biosafety in Plant Biotechnology, Federal Research Institute for Cultivated Plants, Julius Kuehn Institute, Erwin-Baur-Straβe 27, 06484, Quedlinburg, Germany; 3 Institute for Resistance Research and Stress Tolerance, Federal Research Institute for Cultivated Plants, Julius Kuehn Institute, Erwin-Baur-Straβe 27, 06484, Quedlinburg, Germany; 4 Instituto de Biología Molecular y Celular de Plantas (CSIC-UPV), Campus UPV CPI 8E, Ingeniero Fausto Elio s/n, 46022 València, Spain; 5 The Santa Fe Institute, 1399 Hyde Park Road, Santa Fe NM87501, USA

## Abstract

Genetic diversity and recombination patterns were evaluated for 229 isolates of *Wheat dwarf virus* (WDV), which are important cereal-infecting geminiviruses. Recombination hot spots were concentrated at the boundary of the genes encoding for the replication protein (*Rep*), the coat protein (*cp*) and the movement protein (*mp*), as well as inside *Rep* and *cp* and in the short intergenic regions (SIR). Phylogenomic analyses confirmed that the global population of WDV clustered into two groups according to their specific host: wheat and barley, and the crucial regions for the division of two groups were *mp* and the large intergenic regions (LIR). The computationally inferred pattern of coevolution between amino acid residues and the predicted 3D structure for the viral proteins provided further differences among the strains or species at the genome and protein level. Pervasive interaction between Rep and Rep A proteins in WDV-wheat-specific group reflected their important and complex function in the replication and transcription of WDV. Furthermore, significant predicted interactions between CP and Rep and CP and Rep A proteins in the WDV-wheat-specific group are thought to be crucial for successful encapsidation and movement of the virus during infection.

Geminiviruses are characterized by one or two small circular single-stranded (ss) DNA genomes and classified into four genera: *Mastrevirus*, *Curtovirus*, *Topocuviru*s, and *Begomovirus* based on viral vectors, host range and genomic characteristics^1^,^2^. In the past 20 years, geminiviruses have caused devastating yield losses in vegetable and field crops, including tomato, beans, cassava, cotton, cucurbits, pepper, maize, and wheat^3^. Many factors contribute to the emergence and spread of geminiviruses, including the evolution of variants through mutations, recombination and pseudo-recombination; acquisition of satellite-like DNA molecules; changes in cropping systems; and increases in the population and distribution of the insect vectors^1^,^3^. In addition, global climatic changes and human activity have also played an important role in the emergence of serious geminiviruses-induces diseases^4^,^5^. The genetic diversity reflected in present-day geminiviruses will provide important insights into the evolution and biology of these important viruses.

A number of key evolutionary steps must have occurred allowing geminiviruses to continue their adaptation to plants. Estimates of short-term and longer-term evolution rates are available for geminiviruses. The substitution rates of *Beet curly top virus* (BCTV) and *Maize streak virus* (MSV) are as high as 1.5 × 10^−4^ − 3.8 × 10^−4^ substitution/site/year, similar to that of RNA viruses^6^,^7^. Recombination has played and continues to play a pivotal role in geminiviral evolution, whose genome organization and rolling circle replication (RCR) have the potential to induce recombination in different parts of the genome^4^,^8^,^9^. A number of different mechanisms could be responsible for the observed patterns of recombination in geminiviruses, such as replication origins^10^, sequence similarity^11^, ssDNA secondary structure^12^, transcription–replication clashes^13^, and differential degrees of ssDNA exposure within mini-chromosomes^14^,^15^. In the *Curtovirus* genus, the *v-ori* represents a recombination hotspot because of the replication release of viral genomes from genomic concatemers produced during RCR^9^. In some members of the *Begomovirus* genus, recombination can often be easily triggered between or among species that have identities as low as 82%^16^. Strong statistical evidence of recombination hotspots was detected near the coat protein gene (*cp*)/short intergenic region (SIR) interface and at the *v-ori*, while a recombination cold spot spans almost the entire *cp* of MSV, a typical member of the *Mastrevirus* genus^17^.

*Wheat dwarf virus* (WDV) belongs to the genus *Mastrevirus* within the family *Geminiviridae*, which was first reported in the western parts of the Czech Republic and then in many other parts of the world^18^,^19^. Its ssDNA genome encodes four proteins: the coat protein (CP) and the movement protein (MP) on the viral sense strand and two replication-associated proteins (Rep and Rep A) on the complementary strand^19^. The *Rep* gene of WDV possesses an intron of 86 nucleotides^19^,^20^. The 3′ terminal part of the *mp* gene also serves as the initiation site of the *cp* gene, which is translated in the +1 reading frame relative to the *mp* reading frame^19^. The presence of an intron in the *Rep* gene makes it possible to produce two forms of the replication protein, Rep and Rep A. Splicing out of an intron leads to a larger Rep protein. Large and small intergenic regions (LIR and SIR, respectively) contain regulatory elements for viral replication and transcription. The LIR comprises the origin of the RCR^21^. The SIR contains polyadenylation signals and a region to which a short complementary primer for the second strand synthesis binds^22^.

On the basis of a survey of field-derived cereal samples and on DNA sequences of distinct virus isolates, Schubert *et al.* (2007) proposed that WDV should be divided into three species: WDV, *Barley dwarf virus* (BDV) and *Oat dwarf virus* (ODV)^23^. Muhire *et al.* (2013) suggested ODV should be considered as a separate species and that WDV should be divided into five strains (named as A to E) based on genome-wide phylogenetic analyses, which have been approved by the executive committee of the ICTV^24^. The LIR region was first checked for the recombination breakpoint in population of limited 28 Chinese isolates^25^, then with larger set of 30 isolates of WDV, and recombination between WDV and BDV was uncovered with many more hotspots, including the central part of the *cp*, the 3′-terminal part of *Rep*, the 5′-terminal part of *Rep*/*RepA* and the SIR^26^.

In recent years, as wheat dwarf disease has spread more extensively across Europe, Africa and Asia^27^,^28^,^29^, many isolates of WDV have been sequenced, providing the opportunity to check whether new genetic variants have been generated by mutation and recombination. To explore this issue, we used the full genome sequences of 229 WDV and 1 ODV isolates to further reveal the population genetic structure and patterns of molecular evolution of these cereal-infecting geminiviruses. We also looked for discrepancies in the strains or species at the genome level for further research and understanding. Particular attention was given to prove whether the recombination patterns previously observed in other geminiviruses were conserved in WDV^17^,^30^. If so, the difference between strains or species of the WDV and ODV should be reflected in coevolutionary patterns among amino acids both at the intra- and the inter-proteins level of these viruses.

## Results and discussion

### Phylogenetic analyses of WDV and ODV populations

**Figure 1:**
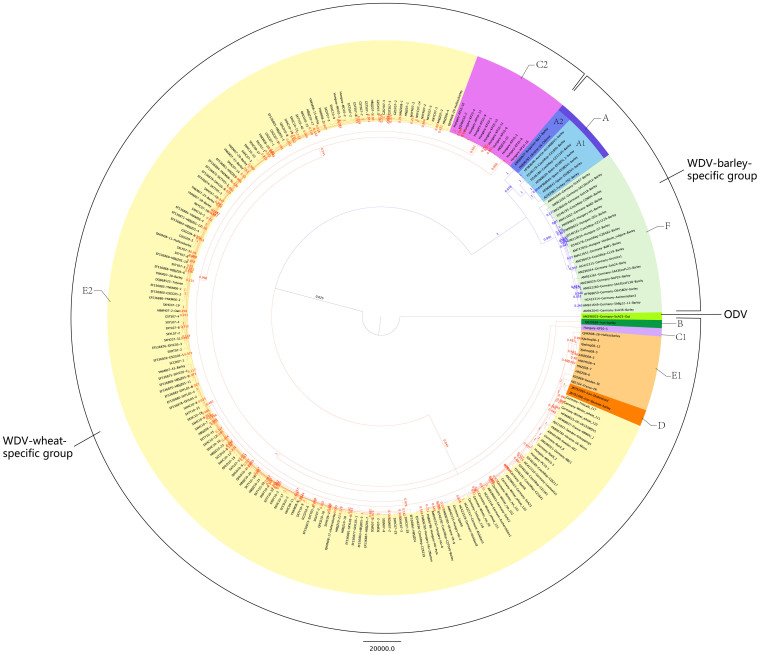
Phylogenetic maximum credibility clade (MCC) tree obtained for the 230 isolates of cereal-infecting geminiviruses. WDV was separated into A, B, C, D, E, and F strains, labeled in different colors in the tree.

For further identifying different strains within this worldwide WDV sample of 230 isolates, pairwise nucleotide identities were computed and a phylogenetic maximum credibility clade (MCC) tree generated (Fig. 1 and Supplementary Fig. S1, nucleotide identities are not shown). The resulting MCC tree was divided into WDV and ODV clusters. In the WDV cluster, WDV-wheat-specific and WDV-barley-specific groups were significantly separated. Six strains (A–F) were detected based on the sequence similarity between isolates and the phylogenetic relationship; A and F strains were mainly from barley and B–E were mainly form wheat, as clearly indicated in the Fig. 1; sequence similarities within strains A–E were in the same range previously shown by Muhire *et al*. (2013)^24^, and isolates from the new strain F had sequence similarities ranging from 97.86% to 100% (Supplementary Table S1), and <94% with isolates from other clusters; for instance, the representative strain of type A^24^, AJ783960 had similarities <94% with all those here included in the F group, being the lowest similarity of 93.19%. D strain, constituted only by two isolates from Iran, had the lowest similarity (94.11%), while A strain was in the order with 94.37% (Supplementary Table S1). For minimizing the distorting effect that recombinant genomes may have on the phylogenetic relationship and identifying which of the viral genes may better help to define the two taxonomic groups, MCC trees with 209 non recombinant isolates were constructed using BEAST version 1.5.4^31^. The MCC trees for *cp*, *mp*, *Rep*, LIR, and SIR + Intron (Supplementary Fig. S1) all showed three major clusters: a WDV-wheat-specific group, a WDV-barley-specific group and ODV. Moreover, the isolates did not group strictly according to the host. The genes and intergenic region that better supported this division in two groups were as *mp*, LIR and SIR + Intron. For example, FJ620684-Iran-barley clustered with the WDV-wheat-specific group on the genomic sequence, *cp* and *Rep*, respectively, while in the MCC trees of *mp*, LIR and SIR + Intron, the strain belonged to the WDV-barley-specific group.

The clusters reflected their geographical origin. The WDV-wheat-specific group had three branches; one with only Chinese isolates, the second with isolates from Europe and Iran, and the third branch with only the Iranian isolate JN791096-Iran-Bavanat-barley, which is the most ancient WDV isolate characterized so far. Isolates from Qinghai and Xinjiang regions of China occupied a basal position in the cluster of the Chinese population. The WDV-barley-specific group formed two clusters, one from Asia, another one from Europe. Only one German isolate of ODV was identified, and it always represented an independent branch in the MCC trees, regardless of the gene used.

Phylodynamic analyses help to relate the phylogenetic history of a pathogen with its genetic variation, selection, transmission, and other epidemiological characteristics^32^. Though the high similarity of the genomic sequences lowered the bootstrap support for the branches, the MCC trees of *mp*, *cp*, *Rep* and LIR showed the presence of three distinct clusters: WDV-wheat-specific group, WDV-barley-specific group and a third groups formed only by ODV (Fig. 1 and Supplementary Fig. S2), consistent with previous reports^23^,^24^,^25^,^26^,^33^. In our analysis with 229 isolates, a new strain was discovered, WDV-F, which is mainly found in Germany and mainly isolated from barley. Based on the genome-wide pairwise identity, isolates AJ783960, FJ620684, JQ647455, JN791096, and AM040732 were considered to be the reference strains for the five WDV strains^24^. However our recombination analyses have shown that isolates AJ783960 and JQ647455 were recombinants, so they must not be considered as good representatives. Moreover, isolates from China, France and Sweden grouped together, consistent with a previous report^25^. In the present study, the oldest isolates in WDV population were from Iran. Not surprisingly, Iran is in the Mesopotamia area, the origin of agriculture and of cultivated wheat^34^.

In this study, we based the definition of strains only on two criteria, namely the pairwise percentage of nucleotide identity and the consistency of groups in the MCC phylogenetic tree, similar to what was previously done by Muhire et al. (2013)^24^. Regarding possible biological differences between strains, it was not possible to see any differential symptoms among isolates. However some differences exist in term of host range: isolates belonging to the wheat-specific group infect barley, wheat, oat, rye, and triticale, while isolates from the barley-specific group infect only barley and only very rarely wheat (Habekuss, unpublished). Therefore, to some degree, these differences in host range give means the molecular data are supported by biological data to the clustering obtained from genomic sequence identities. Nevertheless relevant differences among strains on their biology need to be further explored, such as the infectivity rate of the leafhopper vector, differences in protein functions and so on.

### Evidence for recombination of WDV

**Figure 2:**
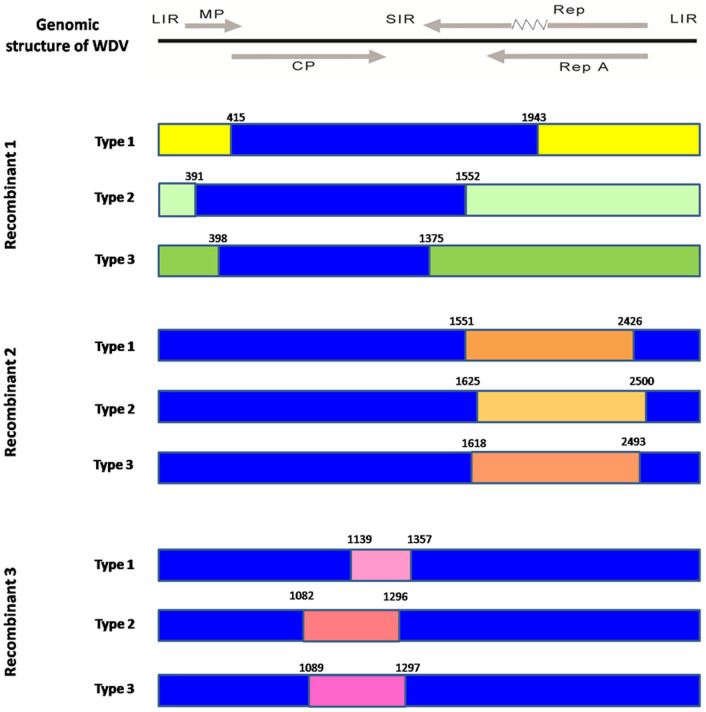
Recombination events detected for the 229 isolates of *Wheat dwarf virus* and 1 isolate of *Oat dwarf virus*. The results showed three recombinants. The genomic structure of WDV was marked by gray color on the top of the figure, in which the *mp* and *cp* were on the positive-sense strand, whilst *Rep*, *Rep* *A* and intron were indicated with a zigzag line were on the antisense strand. The three recombinants have three different configurations. Nucleotide sites in the genomic sequence are labeled. Each color represents a different type of recombinant. The blue framework represents the genomic structure of WDV.

The split-decomposition network analysis showed the existence of conflicting phylogenetic signals among the 230 sequences including the ODV isolate (Supplementary Tables S2 and S3). This intricate reticulate-like pattern of evolution is consistent with extensive recombination among viral genomes, in contrast to a purely bifurcating tree that would reflect a steady diversification due to accumulation of point mutations. Recombination events were detected by seven of the eight statistical methods implemented in RDP4 with high probability (range *P* = 3.72 × 10^−83^ to 1.14 × 10^−3^). Overall, three events and 21 recombinant genomes were found. In general, recombination breakpoints were localized on the boundary of *Rep*, *cp* and *mp*, and inside *Rep*, *cp* and SIR (Table 1 and Fig. 2).

Recombinants in the first event were from the C strain, resulting from an exchange between the A and F strains, the breakpoints were laid in the middle of the intron (at about site 1943), the 5′ end of *cp* (at about nucleotide site 415; amino acid M1), and the 3′ end of *Rep* (at about nucleotides 1552 and 1375; amino acids Y320 and Y322) and *mp* (at about residues 391 and 397; amino acids I72 and G73) (Table 1). The diagnosed recombinants were from Hungary and China with major parental sequences from Germany, Ukraine and Hungary, while minor parental sequences were from China. The reassembled fragments from the minor parent were 2093 nucleotides long. The second event was also from strain C with a single isolate from recombination between strains A and F with 1375 nucleotides, which affected the inner boundary of *Rep* (nucleotides 2500, 2493, 2426, 1625, 1618, and 1551; amino acids Y29, P7, G296, M321, P298, and A349). The regions with the 3′ terminus of *cp* (ca. nucleotide 1139; amino acid F242) as well as inside *cp* (residues 1082 and 1089; amino acids L223 and L225) and SIR (nucleotides 1296, 1297 and 1357) reflected the breakpoint locations of the third recombinant, which was from strain A, resulting from an exchange between strains F and B. The recombinants were mainly from European countries, such as Czech Republic, Spain, Austria, and Turkey. The major parental sequences were from Bulgaria, Ukraine and Germany, while the minor parental sequence in all cases was from isolate FJ620684-Iran-barley, donating 208–222 nucleotides.

The results of the three recombinant events support the following conclusions: (1) About 66.7% of the recombinants occurred between isolates from the WDV-wheat-specific group and the WDV-barley-specific group with about 2093 and 1375 nucleotides in the alignment, 33.3% of the recombinants were triggered in recombinations of the WDV-barley-specific group with about 210 nucleotides in the alignment. (2) Most recombination breakpoints were localized in the complementary sense genes. (3) Hot spot breakpoints were checked on the structural proteins of CP and Rep. (4) Recombinants resulted from the exchange of genetic material from viruses that are geographically distant (e.g., involving two or three countries). For instance, recombinant strains having a fragment from a German parental virus and another fragment from a Chinese parental virus were found in Hungary (Hungary-KP10-5). (5) The inter-strain recombination is common in WDV population.

Previous investigations on recombination of *Mastrevirus* or *Geminivirus* have been incomplete; our study thus aimed to provide the strongest evidence for the role of recombination in the diversification of the genus *Mastrevirus*. Previous investigations on MSV^8^ and WDV^26^ populations showed that v-*ori* was a combination hotspot, but we did not check this in the present study. However, the recombination event in the CP and Rep was similar between *Nanovirus* and *Geminivirus* DNA-1 satellite molecular data sets^7^. Mounting evidence from experimental and computational analyses suggests that the *cp* is a cold spot of recombination in mastreviruses and geminiviruses^8^,^15^,^30^. In our present study, we used the concatenated sequence of all of the genes and intergenic regions, while others used linear genomic sequences for analysis^8^,^26^,^35^. We first checked the CP protein as the hotspot of recombination in *Mastrevirus* and found the CP's breakpoints at amino acid positions L223, I/V226 and F242. Below, we will show that these breakpoints are under significant purification selection. Strong evolutionary constraints also existed for the R/G81, D/N83, Y320, Y322, I263, and P298 sites of Rep. Monjane *et al.* (2001)^36^ showed that Rep had a higher average degree of potential fold disruption than the CP did, which also reflected the greater stability of the CP with respect to Rep. Transcription–replication clashes in the structural proteins of CP and Rep is one potential mechanism to explain the very common recombination. Therefore, further studies should uncover the importance of the recombinant fragment to the fitness of different isolates, such as in the second recombinant event, in which the minor exchanged sequences in Rep were fatal to the adaptation of isolate Hungary-KP10-5, which could better explain the replication of the isolate in the Hungarian wheat. These results also indicated a significant geographic structure; gene flow may still occur at the global scale. Moreover, in general, recombinant strains resulted from the exchange of a single fragment (i.e., a single recombination event); however, there were so many isolates that resulted from two or three recombination events, generating highly mosaic genomes.

The key factor determining the survival of recombinants is the degree to which recombination disrupts coevolved intra-genomic interactions^15^. At the whole-genome scale, potentially disrupted interactions could include sequence–specific interactions between viral proteins, DNA and RNA^15^. Here, we focused on potential disruptions of sequence-specific interactions in the individual virus caused by the recombination and checked D346 of Rep in the WDV-wheat-specific group for a breakpoint of recombination and its implication in coevolving groups of amino acids (see below).

### Codon selection analyses

We evaluated the selective constraints that potentially operated at each codon for each protein for three data sets: (1) for the whole population (209 isolates), (2) for the WDV-wheat-specific group (184 isolates) and (3) for the WDV-barley-specific group (24 isolates). In the whole population as well as the WDV-wheat-specific group, all proteins showed evidence of purification selection. However, Rep A showed the strongest selection pressure, followed by MP, Rep, and CP (Supplementary Table S4). In sharp contrast, in the case of the WDV-barley-specific group, MP was under positive selection, whereas the other proteins were under strong negative selection. The CP was the most conserved protein for both groups. The discrepancy in selection pressure on the proteins reflected the difference in the two groups. The region that affected the clustering of the two groups was Rep in the WDV-wheat-specific group; but in the WDV-barley-specific group, MP was the best. Interestingly, recombination breakpoints on P7, Y320 and Q351 of Rep were under significant positive selection, while most other breakpoints were either under purifying selection or neutral evolution with 79% rate (marked in the lower right corner of the amino acid sites in Table 1 and Supplementary Table S5).

### Coevolution and binding site analyses

**Figure 3:**
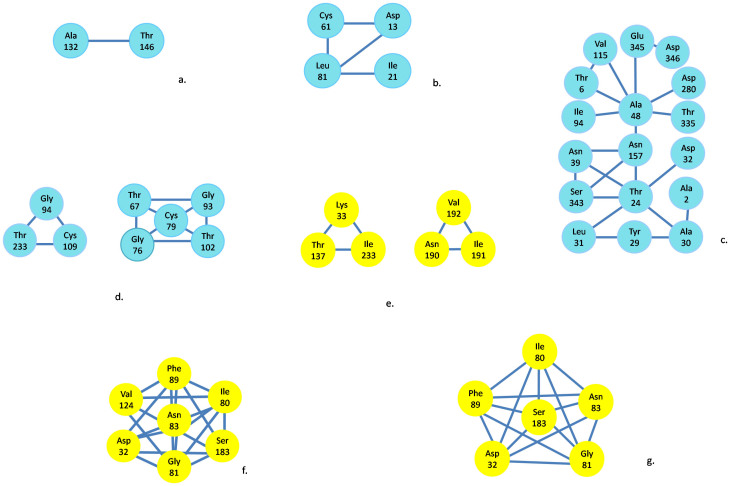
Results of the analyses done to detect groups of amino acids coevolving intra-protein for *Wheat dwarf virus*-W and *Wheat dwarf virus*-B populations. Networks of coevolving amino acid sites within the protein; the three-letter code for amino acids is used. Sites coevolving within (a) WDV-W CP; (b) WDV-W MP; (c) WDV-W Rep; (d) WDV-W Rep A; (e) WDV-B CP; (f) WDV-B Rep; (g) WDV-B Rep A. Residues in the four proteins of WDV-W are in blue; residues in the four proteins of WDV-B are in yellow.

**Figure 4:**
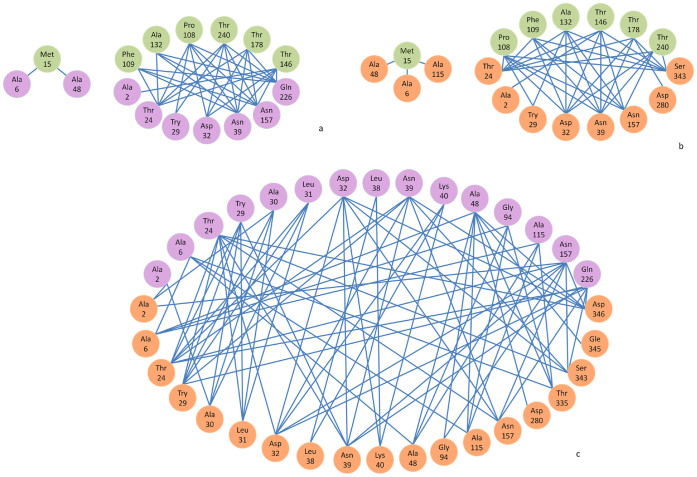
Inter-proteins networks of coevolving amino acids detected for *Wheat dwarf virus*. Network of coevolving amino acid sites within the protein; three-letter code for amino acids is used. (a) CP and Rep A; (b) CP and Rep; (c) Rep and Rep A. CP residues are in green, Rep residues are in orange-yellow, Rep A residues are in purple.

We hypothesized that overlapping coding sequences of CP/MP and of Rep/Rep A should be coevolving and that amino acid residues within a single protein also could be interacting. To uncover any differences between two WDV-host-specific groups, coevolution analyses of intra- and inter-proteins were conducted. Through the analysis with the algorithms implemented in the software CAPS^37^, significant traits were found in two groups of WDV (Fig. 3 and Table 2). Above all, four proteins of the WDV-wheat-specific group show evidences of intra-protein coevolving sites, as did the proteins of the WDV-barley-specific group, except for the MP. The coevolving sites of each protein had its own characters. For the CP protein, the WDV-wheat-specific and WDV-barley-specific groups did not have the same sites. The wheat-specific group only had two site residues (A132 and T146), while the CP of the barley-specific group had six site residues (K33, T137, N190, I191, and V192, I233) (Fig. 3 and Table 2). In the WDV-wheat-specific group, Rep had more coevolving sites than Rep A (Table 2); however, the two proteins had the same I94 site, which means it may play an essential evolutionary role for both proteins. The status of the coevolving inter-protein sites differed in the two groups of isolates. For the WDV-wheat-specific group, the coevolving sites between proteins were only reflected in CP/Rep, CP/Rep A and Rep/Rep A, while in the WDV-barley-specific group, the four proteins did not interact with each other at all. CP/Rep and CP/Rep A of the wheat-specific group involved six groups of coevolving sites with the same seven amino acids in CP (M15, P108, F109, A132, T146, T178, and T240) (Fig. 4 and Table 2); these sites should play a functional role all together. In the WDV-wheat-specific group, Rep and Rep A, the A2, A6, T24, Y29, D32, N39, A48, and N157 were not only coevolving inter-protein, but also intra proteins, which also reflected their functional potential. Relationships of the inter-proteins MP/Rep or MP/Rep A were not found in either group. The MPs of the two groups did not have interactive sites with other proteins. However, MP could interact with CP and itself in the WDV-wheat-specific group, while it could not do so with CP or itself in WDV-barley-specific group. Since only one ODV isolate was available, we excluded it from the covariation analyses.

The distribution of the positively selected sites along the tertiary structure of the proteins may illustrate which parts of the structure are more or less affected by selection^38^. In general, the evolutionary forces were unevenly distributed on every protein. In the WDV-wheat-specific group, CP only has one amino acid, A132, under positive selection in the two coevolving sites, and three fourths of the coevolving sites of the MP were under positive selection. At the same time, as the lapped site for coevolution of inter-protein with Rep and Rep A, the F109, T146, T178 and T240 of CP were under negative selection, which reflected their strong conservation involving in an important function. In Rep, 44% of the intra-protein coevolving sites were under negative selection or evolving neutrally, while in Rep A, only one eighth of intra-protein interactive sites were under positive selection. In the coevolving sites of inter-proteins CP/Rep and CP/Rep A, A2, A6, T24, Y29, D32, N39, A48, and N157 were common for both interactions, 56% of which were under positive selection. In the WDV-barley-specific group, most intra-protein coevolving sites of CP, Rep and Rep A were identified as under positive selection. I191 and I233 of CP as well as F89 of Rep and Rep A were under negative purifying selection.

Moreover the D346 amino acid site of WDV-wheat-specific group was the only site involved both in significant coevolutinary interactions and also in recombination events. The extent in which changes in D346 may disrupt the integrated function of coevolving groups and their implication in establishing the right folding/function needs to be evaluated. To our knowledge, recombination and coevolution analyses have never been used to analyze any naturally generated chimeric proteins; we realized they should also be useful for understanding breakpoint distribution patterns found within coding regions of recombining virus genomes.

Mapping predicted binding site residues on the predicted fold of each protein helped to highlight the differences between WDV and ODV (Table 3). Comparing the CP from two groups of WDV, we found that they shared the same coevolving sites: A123, V158, V159, K160, and R202 except for V203 of the CP in the barley-specific group. However, ODV CP had totally different highlighted binding sites, thus suggesting that different patterns of binding sites exist among the CP structures of WDV and ODV. In MP and Rep, the predicted binding sites residues were also quite different between WDV-wheat-specific and WDV-barley-specific groups and ODV (Table 3). Furthermore, whereas the amino acid identity between any two representatives of CP from WDV-wheat-specific group and barley-specific group was 84.6%, the amino acid identity between CP of WDV-wheat-specific group and ODV was 72.4%, and 73.9% between the CP of the WDV-barley-specific group and ODV, which also supports the divergence between ODV and the WDV types.

Through these coevolutionary analyses and comparing the predictions of 3D structures generated by *in silico* protein folding algorithms for every protein, discrepancies among the WDV-wheat-specific group, the WDV-barley-specific group and ODV were further uncovered. Complex networks of evolutionary dependencies among amino acid residues helped to identify differences in the selective constraints that have been imposed on the two species (WDV and ODV), as well as to understand their functional significance in previous and future studies. Our bioinformatics results are a good complement to previous observations. For instance, CP proteins in *Geminivirus* were not only responsible for the encapsidation function, but also executed intra- as well as inter-plant virus transmission and yielded viral DNA with an ATPase function through the packaging and triggering of the replication function of Rep in the infected tissues^30^,^39^. There is a reasonably strong interaction between Rep and CP reported in *Mung bean yellow mosaic India virus* in the region of Rep spanning amino acids H120 to 362 bound by GST-CP2 in yeast cells^40^. In the WDV-wheat-specific group, the CP had intra-protein interaction and inter-protein interaction with Rep and Rep A, while the WDV-barley-specific group did not. From this case, the difference between two groups was significant. Strong interactions for Rep A/Rep A and Rep/Rep were previously found for MSV^41^. In our study, we observed significant interactions between Rep A and Rep for the WDV-wheat-specific group, but we failed to detect similar interactions in the WDV-barley-specific group.

In the research of Schubert *et al.* (2014)^26^, because of the limited set of 30 sequences, recombination was uncovered only within WDV-B strain. Our research with a much larger set of WDV isolates revealed that recombinants were triggered between the WDV-wheat-specific group and the barley-specific group, and we also evaluated purification selection as a modulator of the observed recombination pattern. Following the naming of the WDV strain and establishment of classification standards for *Mastrivirus*^24^, we suggested that a new strain, F, should be proposed. At the same time, the discrepancies between the WDV-wheat-specific and barley-specific groups were further explored using coevolutionary networks of amino acids and 3D structure prediction of proteins, including coevolving amino acid residues at the inter- and intra-protein levels, binding site residues and predicted active site residues analyses. Future studies should continue testing and verifying the characteristics of every protein of WDV and ODV, e.g using yeast two-hybrid, BiFC or co-immunoprecipitation experiments.

## Methods

### Virus isolates

A total of 171 WDV isolates were collected throughout China, Germany and Hungary during field surveys in the growing seasons from 2004 to 2011. The isolate names, their hosts, time and sites of collection are given in Supplementary Table S2. All isolates were amplified by polymerase chain reaction (PCR) followed by sequencing of amplicons. We also included 59 full genomic sequences of WDV isolates from other countries that were already available in GenBank (Supplementary Table S3).

### Cloning of entire genomes and sequencing

Total DNA was extracted from wheat leaves systemically infected with WDV^25^. DNA extracts were used as a template for PCR amplification in a 50 μL reaction solution containing 10× Taq Buffer, 2.5 mM dNTP (each), 0.4 mM of the viral sense and complementary sense primers designed according to the conserved sequences of WDV genomes (Supplementary Table S6)^27^,^42^, and 0.3 μL (5 U/μL) Ampli Taq DNA polymerase (Applied Biosystems, Foster City, CA, USA). PCR reactions were carried out for 35 cycles of denaturation at 94°C for 1 min, annealing at 55°C for 1 min, and extension at 72°C for 1 min, with 95°C for 2 min at the first step and 72°C for 10 min at the final step. The expected PCR products were 767 bp, 1152 bp and 1041 bp, using primer pairs 40F/806R, 735F/1886R and 1828F/118R, respectively (the number corresponds to the location of the primers in the genome sequences on the GenBank) (Supplementary Table S6), and together covered the entire length of the viral genome. The PCR product segments were electrophoresed in 1.0% agarose gels and purified by the BioTeq PCR quick Gel Extraction Kit (BioTeq, USA). The purified fragments were cloned into the pMD18-T vector (Takara, Dalian, China) and used to transform *Escherichia coli* strain JM110. Viral DNA from German isolates was amplified by RCA (General Electric Healthcare), and products were digested with appropriate restriction enzymes. The resulting full-length genomic fragments were gel-purified and cloned into pGEM-T (Promega, USA) previously digested with HindIII.

### DNA sequencing

Insert sequences were determined for two to three clones for each fragment using either the ABI (ABI BigDye 3.1, Applied Biosystems) or Beckman Systems (GeXP with Genome Lab DTCS sequencing kit). Sequence data were assembled using DNASIS version 3.5 (Hitachi, Tokyo, Japan), Laser gene (DNASTAR, Madison WI, USA) or BIOEDIT version 5.0.9^31^.

### Sequence alignments

Every cistron and noncoding region was identified and aligned independently with MUSCLE^43^ as implemented in MEGA version 5.0^44^. The six resulting alignments were then concatenated into a single long alignment with the order LIR + *mp* + *cp* + SIR + *Rep* + intron.

### Recombination analysis

SplitsTree version 4 was first used for the split-decomposition network analysis^45^. Then, the recombination breakpoints were identified using the methods RDP, GENECONV, BOOTSCAN, MAXCHI, CHIMAERA, SISCAN, and 3SEQ implemented in RDP4 and using the default configuration^46^. Only those recombination events predicted by at least five of the implemented methods were taken as valid. Recombinant genomes were discarded from the data set in all subsequent phylogenetic and selection-detection analyses.

### Phylogenetic analysis

Maximum credibility clade (MCC) phylogenetic reconstructions were conducted using BEAST version 1.5.4^47^. The best model of nucleotide substitution was determined by MODELTEST version 3.7^48^. The Markov chain Monte Carlo (MCMC) was run for 10^7^ generations to ensure convergence of all parameters. Branches with a posterior support probability <0.50 were collapsed.

### Estimation of selection pressures at different codons

Selective pressures operating at each codon were evaluated based on the difference between synonymous (*d_S_*) and nonsynonymous (*d_N_*) substitution rates for each gene (*cp*, *mp*, *Rep* and *Rep A*) calculated by MEGA version 5^44^. Values of *d_N_* − *d_S_* < 0, = 0 or > 0 indicate purifying selection, neutral evolution and positive selection, respectively.

### Coevolution analysis

To identify correlated variation among amino acid sites, in particular those with evidence of selection pressures, we analyzed coevolution within and between MP, CP, Rep, and Rep A proteins. Coevolution was identified using the program CAPS version 1.0^37^. The algorithm implemented in CAPS has been shown to outperform other coevolution-detection methods^49^. Briefly, this program identifies covariation between pairs of sites in the multiple sequence alignment by calculating the correlation in the variation in amino acid patterns between both sites. The BLOSUM amino acid substitution matrix is then used to score the strength of the amino acid variation for a particular amino acid site, and these scores are corrected by taking into account the divergence time between the sequences of the multiple sequence alignment (measured as the estimated number of synonymous substitutions). The significance of the correlation coefficients was tested using 10,000 pseudo-random pairs of amino acid sites and a confidence value α = 0.001. We also tested whether coevolving amino acids can be used to predict protein–protein contact interfaces. Both intra- and inter-domains analyses were performed.

Structural clustering of coevolving sites could shed light on their functional and structural reciprocal selective constraints. We previously modeled the 3D structure of WDV CP, MP and Rep proteins using the I-TASSER platform, a program that iteratively conducts threading assembly refinement starting with a single amino acid and generating 3D atomic models^50^. The modeling is performed in three stages. First, the query sequence is PSI-blasted against a non-redundant sequence database and secondary structures predicted with PSIPRED^51^. Then, the sequence and the predicted secondary structures are submitted against a PDB structure library using a suit of seven threading programs, all compiled in LOMETS^52^. Second, continuous fragments are excised from threading alignments and assembled to build structural conformations, with the structure of non-aligned regions modeled *ab initio*. Third, a consensus set of models, those that are closest to the centroid of the simulations, is used to refine the models. The final stage of the modeling provides a set of models and their corresponding scores (TM scores), with the highest score referring to the best model.

## Author Contributions

X.W. and B.W. designed the research. B.W. and X.S. performed the experiments, B.W., J.S., A.H. and S.F.E. analyzed the data, B.W., S.F.E. and X.W. wrote the manuscript.

## Supplementary Material

Supplementary Information Supplementary Information

## Figures and Tables

**Table 1:** Results of the recombination analyses performed for the population of *Wheat dwarf virus*

Recombination event number	Recombinant sequence(s)	Minor parental sequence(s)	Major parental sequence(s)	Identity (%) of parental sequences	Sites of breakpoints in alignment of nt 1–2854 (start-end)	Nucleotide site in genome sequences and amino acid site in proteins
1	Hungary-KP10-16	FM999832-Hungary-D01-Barley	EF536870-HBSJZ06-11	83.4	1) nt 2830-733: center of intron and 5' boundary of *cp*2) nt 2751-676: 3' of *rep* and *mp*3) nt 2758-683: 3' of the *rep* and *mp*	1) nt 1943 in the intron and nt 415 in the *cp*: M1^0^* in the CP;2) nt 1552 in the *rep* and nt 391 in the *mp*: Y320 in the Rep and G73^0^ in the MP;3) nt 1375 in the *rep* and nt 397 in the *mp*: Q351 in the Rep and I72^0^ in the MP
	HBSJZ10-10	FN806787-Ukraine-Uk-Odessa	EF536868-HBSJZ06-9	83.9		
	XIZANG10-2	HG422314-Germany-Aschersleben3	EF536863-HBSJZ06-3	82.9		
	Hungary-KP10-1	HG422315-Germany-Krostitz1	EF536867-HBSJZ06-7	83.4		
	Hungary-KP10-3	AM942044-Germany-SxA57-Barley	EF536878-SXYL05-2	83.6		
	Hungary-KP10-4	AM942045-Germany-SxA36-Barley	EF536882-SXYL05-6	83.6		
	Hungary-KP10-5	AM921649-Germany-SABg12-11-Barley	EF536880-SXYL05-4	81.9		
	Hungary-KP10-6	AM296024-Germany-SxA24-Barly	EF536877-SXYL05-1	83.6		
	Hungary-KP10-8	AM296020-Germany-McP20-Barley	EF536881-SXYL05-5	83.6		
	Hungary-KP10-9	AM296018-Germany-SxA18-Barley	EF536875-SXYC05-2	83.8		
	Hungary-KP10-10	AM411652-Germany-BaW2-Barley	EF536876-SXYC05-3	83.7		
	Hungary-KP10-11	AM411651-Germany-BaW1-Barley	EF536873-SXTY05-2	83.9		
	Hungary-KP10-13	HF968650-Germany-DE45BDV-Barley	EF536862-HBSJZ04	83.6		
	Hungary-KP10-15	AM922260-Germany-SA45EcoFL38-Barley	EF536859-GSGG05-1	83.5		
2	Hungary-KP10-5	AM411651-Germany-BaW1-Barley	HNZZ07-7	83.9	1) nt 1793-2668: 5' and 3' of the *rep*2) nt 1719-2594: 5' and in *rep*3) nt 1726-2601: 5' and in *rep*	1) nt 2426 and nt 1551 in the *rep*: Y29— and M321^0^ in the Rep;2) nt 2500 and nt 1625 in *rep*: A349^0^ and G296^0^ in Rep;3) nt 2493 and nt 1618 in *rep*: P7 and P298— in Rep
3	FJ546180-CzechRep-CZ11105-Barley	Unknown (FJ620684-Iran-Barley)	AM989927-Bulgarian-Bg17-Barley	90.7	1) nt 1457-1675: 3' of *cp* and in SIR2) nt 1400-1614: in *cp* and SIR3) nt 1407-1615: in *cp* and SIR	1) nt 1139 in *cp* and nt 1357 in SIR: F242— in CP;2) nt 1082 in *cp* and nt 1296 in the SIR: L223^0^ in CP;3) nt 1089in *cp* and nt 1297 in the SIR: L225— in CP
	FJ546179-CzechRep-CZ1800-Barley	Unknown (FJ620684-Iran-Barley)	FN806787-Ukraine-Uk-Odessa	90.9		
	HF968639-Spain-ES1BDV_1-barley	Unknown (FJ620684-Iran-Barley)	HG422314-Germany-Aschersleben3	89.9		
	HF968641-Spain-ES2BDV1-barley	Unknown (FJ620684-Iran-Barley)	HG422315-Germany-Krostitz1	90.4		
	HF968644-Spain-ES3BDV1-barley	Unknown (FJ620684-Iran-Barley)	AM942044-Germany-SxA57-Barley	90.6		
	HF968646-Austria-AU196BDV1-barley	Unknown (FJ620684-Iran-Barley)	AM942045-Germany-SxA36-Barley	90.4		
	AJ783960-Turkey-TR2-Barley	Unknown (FJ620684-Iran-Barley)	AM921649-Germany-SABg12-11-Barley	88.6		

*^—^ and ^0^ in the upper right corner of the amino acid means negative and neutral selection, respectively.

**Table 2:** List of coevolving amino acid residues both at the intra- and inter-protein levels found for *Wheat dwarf virus*

Intra-protein or inter-protein		Group	Number of amino acids	Number of isolates	Amino acid sites
WDV-wheat-specific group	CP	1	2	184	A132, T146^—^*
	MP	1	4	184	D13—, I21, C61, L81
	Rep	12	17	184	A2, T6, T24, Y29—, A30, L31, D32—, N39, A48—, I94—, V115—, N157—, D280^0^, T335—, S343, E345, D346^0^
	Rep A	2	8	184	T67—, G76—,C79—,G93—, I94—,T102—, C109—, T233
	CP/MP	0	0	184	NA
	CP/Rep	6	7	184	M15, P108, F109—, A132, T146—, T178—, T240— [A2, A6, T24, Y29—, D32—, N39, A48^0^, A115^0^, N157—, D280^0^, S343]
	CP/Rep A	6	7	184	M15, P108, F109—, A132, T146—, T178—, T240— [A2, A6, T24, Y29—, D32—, N39, A48^0^, N157—, Q226—]
	MP/Rep	0	0	184	NA
	MP/Rep A	0	0	184	NA
	Rep/Rep A	14	19	184	A2, A6, T24, Y29—, A30, L31, D32—, L38, N39, K40, A48^0^, G94—, A115—, N157—, D280^0^, T335—, S343, E345, D346^0^ [A2, A6, T24, Y29—, A30, L31, D32—, L38, N39, K40, A48^0^, G94, A115—, N157—, Q226]
WDV-barley specific group	CP	2	6	24	K33, T137, N190, I191—, V192, I233—
	MP	0	0	24	NA
	Rep	2	7	24	D32, I80, G81, N83, F89—, V124, S183
	Rep A	1	6	24	D32, I80, G81, N83, F89—, S183
	CP/MP	0	0	24	NA
	CP/Rep	0	0	24	NA
	CP/Rep A	0	0	24	NA
	MP/Rep	0	0	24	NA
	MP/Rep A	0	0	24	NA
	Rep/Rep A	0	0	24	NA
ODV	CP	0	0	1	NA
	MP	0	0	1	NA
	Rep	0	0	1	NA
	Rep A	0	0	1	NA
	CP/MP	0	0	1	NA
	CP/Rep	0	0	1	NA
	Rep A	0	0	1	NA
	MP/Rep	0	0	1	NA
	MP/Rep A	0	0	1	NA
	Rep/Rep A	0	0	1	NA

*^—^ and ^0^ in the upper right corner of the amino acid means negative and neutral selection, respectively. The sites in the brace are amino acids involved in inter-protein coevolution.

NA: not available.

**Table 3:** Interacting amino acid residues of proteins in *Wheat dwarf virus*-wheat-specific group, *Wheat dwarf virus*-barley-specific group and *Oat dwarf virus* as predicted with the I-TASSER protein folding prediction platform

Protein	Interacting sites
WDV-wheat-specific group CP	A123, V158, V159, K160, R202
WDV-barley-specific group CP	A123, V158, V159, K160, R202, V203
ODV-CP	A120, V155, V156, K157, R199, V200
WDV-wheat-specific group MP	L40, G44, L57, V60
WDV- barley-specific group MP	A45, V49, Y50
ODV-MP	V44, G45, I46, I47, Y48
WDV-wheat-specific group Rep	T108, T117, E119, T186
WDV- barley-specific group Rep	L245, I260, N270, F274
ODV-Rep	N100, C102, D104, E107
