# Supplementary material for: Global-scale computational analysis of genomic sequences reveals the recombination pattern and coevolution dynamics of cereal-infecting geminiviruses
Source: Sci Rep. 2015 Jan 30;5:8153. doi: 10.1038/srep08153 (PMC4311259; doi:10.1038/srep08153)
Supplement: Supplementary Information [file srep08153-s1.pdf]

Supporting Information for

Global-scale computational analysis of genomic sequences reveals the recombination pattern and coevolution dynamics of cereal-infecting geminiviruses

Beilei Wu[1], Xiaonan Shang[1], Jörg Schubert[2], Antje Habekuß[3], Santiago F. Elena[4], Xifeng Wang[1*]

[1]*State Key Laboratory for Biology of Plant Diseases and Insect Pests, Institute of Plant Protection, Chinese Academy of Agricultural Sciences, Beijing 100193, China*

[2]*Institute for Biosafety in Plant Biotechnology, Federal Research Institute for Cultivated Plants, Julius Kuehn Institute, Erwin-Baur-Straße 27, 06484, Quedlinburg, Germany*

[3]*Institute for Resistance Research and Stress Tolerance, Federal Research Institute for Cultivated Plants, Julius Kuehn Institute, Erwin-Baur-Straße 27, 06484, Quedlinburg, Germany*

[4]*Instituto de Biología Molecular y Celular de Plantas (CSIC-UPV), Campus UPV CPI 8E, Ingeniero Fausto Elio s/n, 46022 València, Spain*

*Corresponding author: Email: xfwang@ippcaas.cn

Supplementary Figure S1. Phylogenetic maximum credibility clade (MCC) tree for the 230 isolates of cereal-infecting geminiviruses.

Posterior probability values indicate support for each node, and time scale is shown at the bottom of tree. The isolates painted by the yellow color were the representatives of the strains suggested by Muhire *et al*. (2013). The different strains of WDV were clustered by the vertical virgule of purple color. The branches of WDV-specific group, WDV-specific group and ODV were colored by orange, blue and deep red respectively.

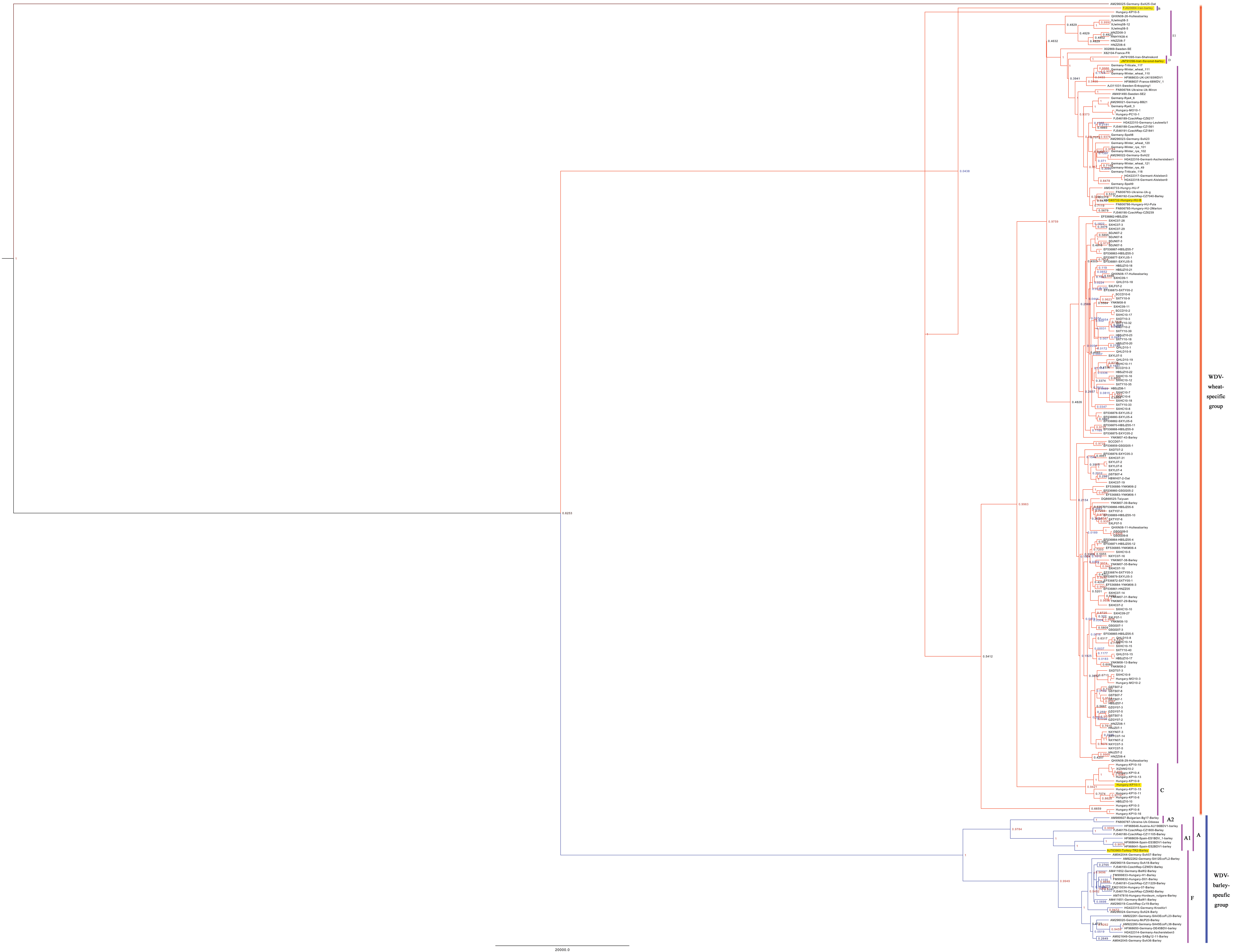

B
E1
D
C
A2
A1
A
F
WDV-
wheat-
specific
group
WDV-
barley-
speufic
group
20000.0

Supplementary Figure S2. Phylogenetic maximum credibility clade (MCC) tree of *mp*, *cp*, *rep*, LIR and SIR+intron for 209 isolates of cereal-infecting geminiviruses.

Posterior probability values indicate support for each node, and time scale is shown at the bottom of tree. The branches of WDV-wheat-specific group, WDV-barley-specific group and ODV were colored by orange, blue and deep red respectively. A: *mp* gene; B: *cp* gene; C: *rep* gene; D: LIR region; E: SIR+inton region.

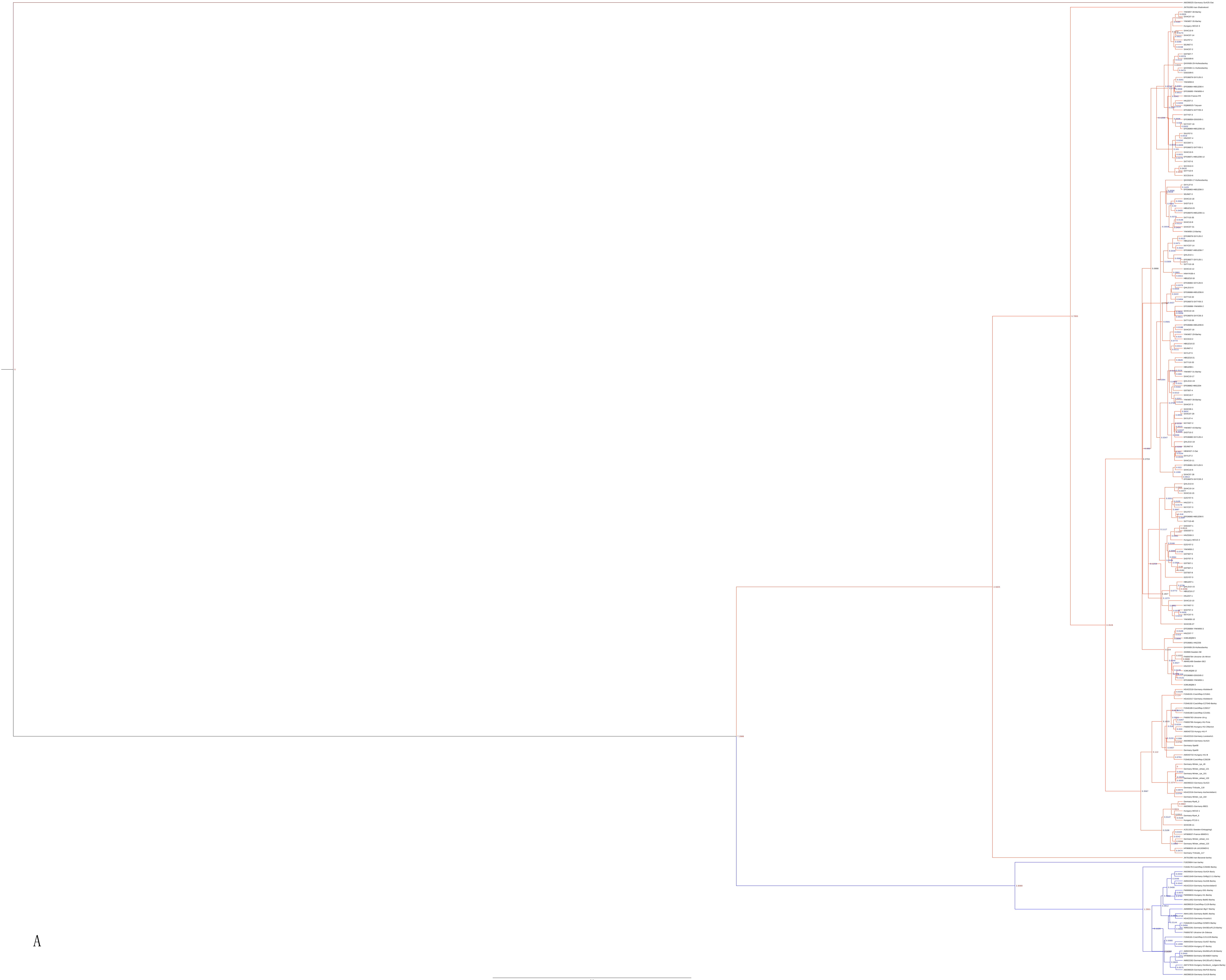

A

B

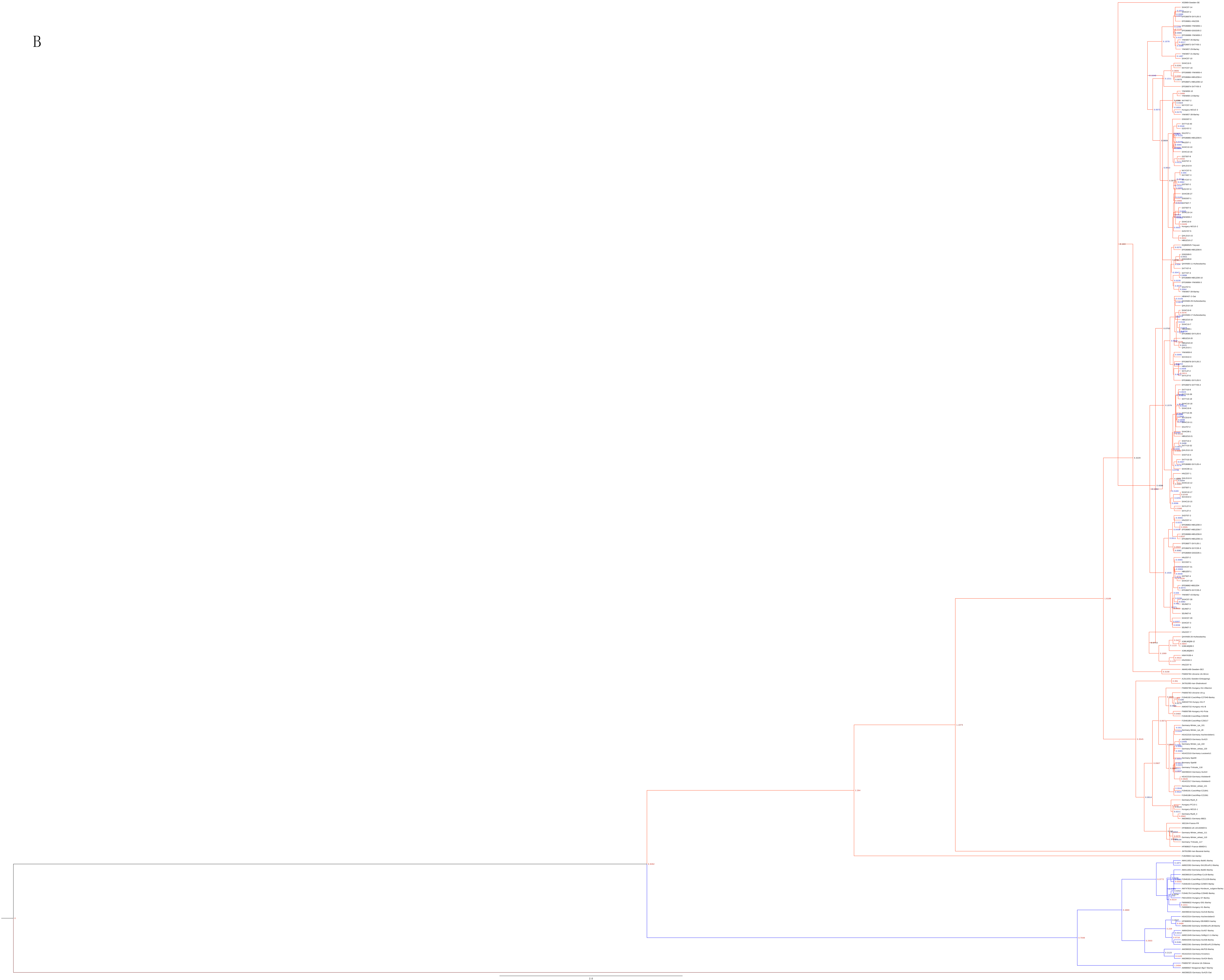

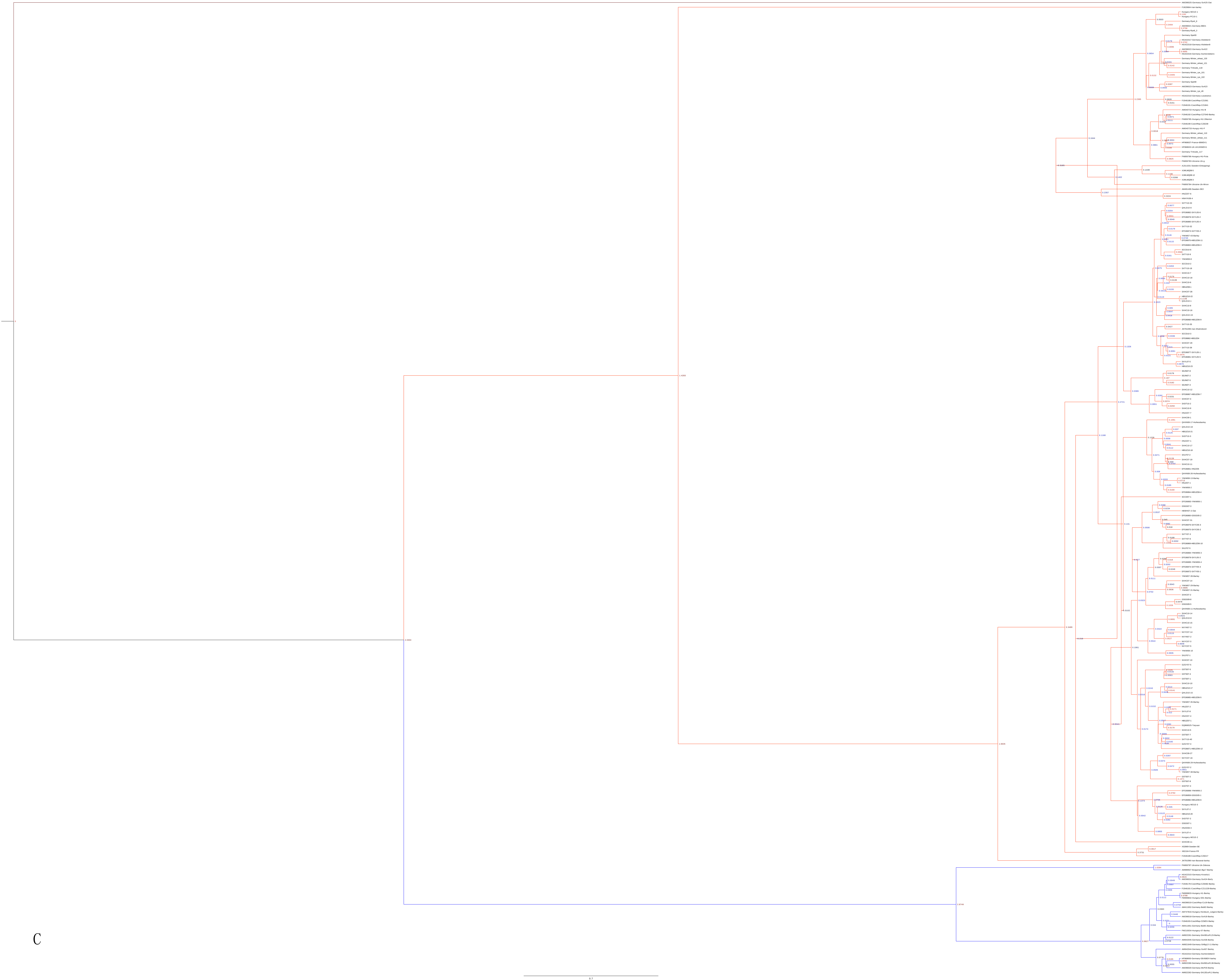

C

D

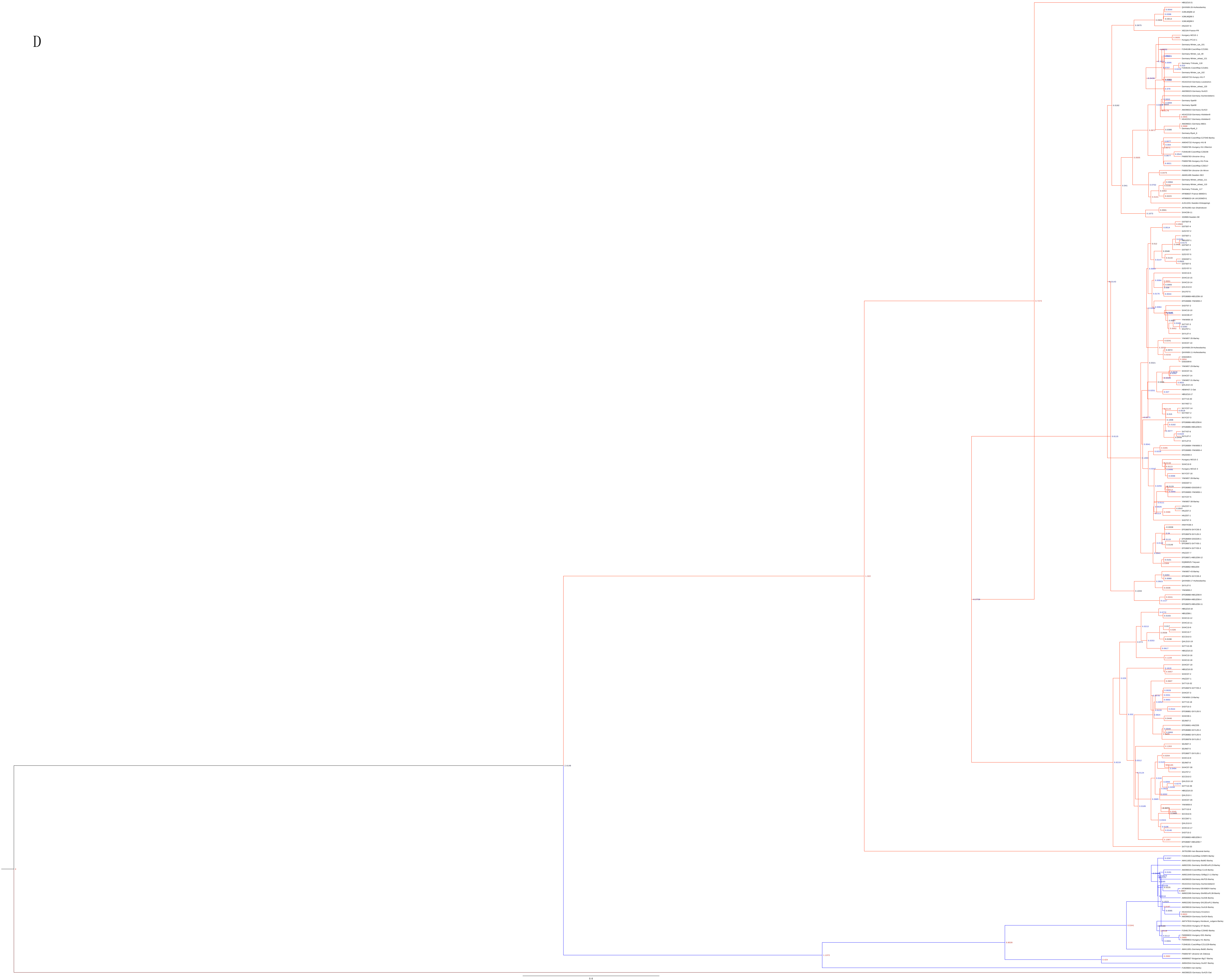

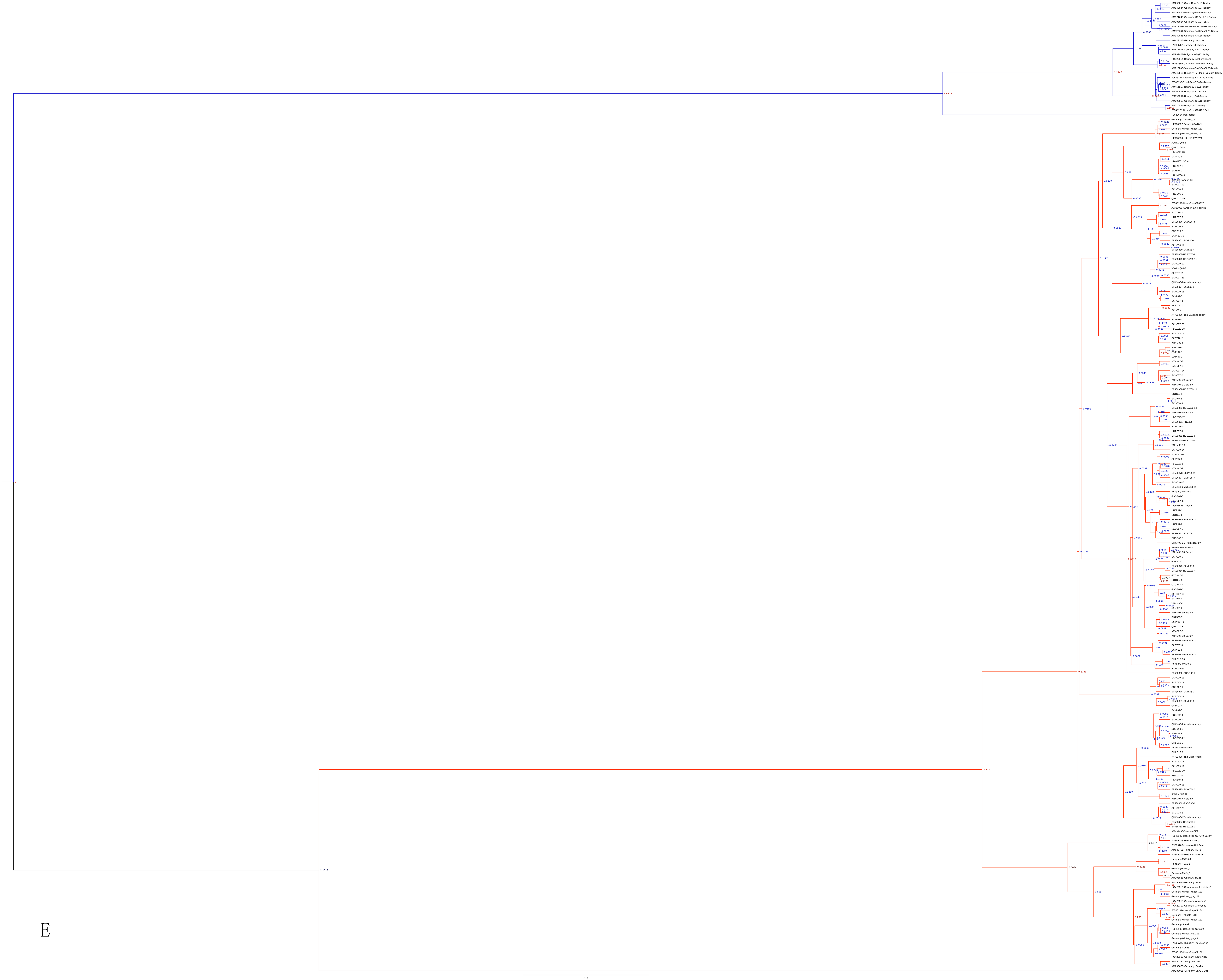

E

Supplementary Table. S1 The percent nucleotide similarities among each of the six strains in *Wheat dwarf virus*

Strains	Lowest identity (%)	Highest identity (%)
A(N:9)	94.37	99.53
B(N:1)	NA	NA
C(N:14)	97.68	100
D(N:2)	94.11	94.11
E(N:181)	96.43	100
F(N:22)	97.86	100

N: The number of the isolates in the group

NA: not available.

Supplementary Table S2. Genomic sequences of *Wheat dwarf virus* obtained in our laboratory and this study.

Years	Isolates/hosts	Collection date	Country	Accession No.
2004	DQ868525/SXTY04/wheat	4-May	China	DQ868525
2005	EF536859/GSGG05-1/wheat	5-Apr	China	EF536859
2005	EF536860/GSGG05-2/wheat	5-Apr	China	EF536860
2005	EF536861/HNZZ05/wheat	5-Apr	China	EF536861
2004	EF536862/HBSJZ04/wheat	4-Apr	China	EF536862
2005	EF536863/HBSJZ05-3 /wheat	5-Apr	China	EF536863
2005	EF536864/HBSJZ05-4/wheat	5-Apr	China	EF536864
2005	EF536865/HBSJZ05-5/wheat	5-Apr	China	EF536865
2005	EF536866/HBSJZ05-6/wheat	5-Apr	China	EF536866
2005	EF536867/HBSJZ05-7/wheat	5-Apr	China	EF536867
2005	EF536868/HBSJZ05-9/wheat	5-Apr	China	EF536868
2005	EF536869/HBSJZ05-10/wheat	5-Apr	China	EF536869
2005	EF536870/HBSJZ05-11/wheat	5-Apr	China	EF536871
2005	EF536871/HBSJZ05-12/wheat	5-Apr	China	EF536871
2005	EF536872/SXTY05-1/wheat	5-May	China	EF536872
2005	EF536873/SXTY05-2/wheat	5-May	China	EF536873
2005	EF536874/SXTY05-3/wheat	5-May	China	EF536874
2005	EF536875/SXYC05-2/wheat	5-Apr	China	EF536875
2005	EF536876/SXYC05-3/wheat	5-Apr	China	EF536876
2005	EF536877/SXYL05-1/wheat	5-Apr	China	EF536877
2005	EF536878/SXYL05-2/wheat	5-Apr	China	EF536878
2005	EF536879/SXYL05-3/wheat	5-Apr	China	EF536879
2005	EF536880/SXYL05-4/wheat	5-Apr	China	EF536880
2005	EF536881/SXYL05-5/wheat	5-Apr	China	EF536881
2005	EF536882/SXYL05-6/wheat	5-Apr	China	EF536882
2006	EF536883/YNKM06-1/wheat	6-Apr	China	EF536883
2006	EF536884/YNKM06-3/wheat	6-Apr	China	EF536884
2006	EF536885/YNKM06-4/wheat	6-Apr	China	EF536885
2006	EF536886/YNKM06-2/wheat	6-Apr	China	EF536886
2007	EU541489/07YNKM29 /barley	2007/3/15	China	EU541489
2007	KJ536143/07YNKM35 /barley	2007/3/15	China	KJ536143
2007	KJ536144/07YNKM38-/barley	2007/3/15	China	KJ536144
2007	KJ536145/07YNKM39-/barley	2007/3/15	China	KJ536145
2007	KJ536146/07YNKM43-/barley	2007/3/15	China	KJ536146
2007	KJ536142/07YNKM31-/barley	2007/3/15	China	KJ536142
2007	KJ536086/07GZGY2-/wheat	2007/3/17	China	KJ536086
2007	KJ536087/07GZGY3/wheat	2007/3/17	China	KJ536087
2007	KJ536088/07GZGY5/wheat	2007/3/17	China	KJ536088
2007	KJ536125/07SCCD1/wheat	2007/3/12	China	KJ536125
2007	KJ536090/07HNJZ1/wheat	2007/4/1	China	KJ536090

2007	KJ536091/07HNJZ2/wheat	2007/4/1	China	KJ536091
2007	KJ536126/07HBWH2/oat	2007/3/15	China	KJ536126
2007	KJ536078/07GSGG1/wheat	2007/4/15	China	KJ536078
2007	KJ536079/07GSGG3/wheat	2007/4/15	China	KJ536079
2007	KJ536080/07GSTS1/wheat	2007/4/16	China	KJ536080
2007	KJ536081/07GSTS2/wheat	2007/4/16	China	KJ536081
2007	KJ536082/07GSTS4/wheat	2007/4/16	China	KJ536082
2007	KJ536083/07GSTS5/wheat	2007/4/16	China	KJ536083
2007	KJ536084/07GSTS7/wheat	2007/4/16	China	KJ536084
2007	KJ536085/07GSTS8/wheat	2007/4/16	China	KJ536085
2007	KJ536111/07SXHC2/wheat	2007/5/15	China	KJ536111
2007	KJ536114/07SXHC3/wheat	2007/5/15	China	KJ536114
2007	KJ536108/07SXHC10/wheat	2007/5/15	China	KJ536108
2007	KJ536109/07SXHC14/wheat	2007/5/15	China	KJ536109
2007	KJ536110/07SXHC19/wheat	2007/5/15	China	KJ536110
2007	KJ536112/07SXHC28/wheat	2007/5/15	China	KJ536112
2007	KJ536113/07SXHC29/wheat	2007/5/15	China	KJ536113
2007	KJ536115/07SXHC31/wheat	2007/5/15	China	KJ536115
2007	KJ536121/07SXYL2/wheat	2007/4/17	China	KJ536121
2007	KJ536122/07SXYL4/wheat	2007/4/17	China	KJ536122
2007	KJ536123/07SXYL5/wheat	2007/4/17	China	KJ536123
2007	KJ536124/07SXYL8/wheat	2007/4/17	China	KJ536124
2007	KJ536089/07HBSJZ1/wheat	2007/4/18	China	KJ536089
2007	KJ536116/07SXLF1/wheat	2007/4/27	China	KJ536116
2007	KJ536117/07SXLF2/wheat	2007/4/27	China	KJ536117
2007	KJ536118/07SXLF5/wheat	2007/4/27	China	KJ536118
2007	KJ536102/07SDJN2/wheat	2007/5/10	China	KJ536102
2007	KJ536103/07SDJN3/wheat	2007/5/10	China	KJ536103
2007	KJ536105/07SDJN5/wheat	2007/5/10	China	KJ536105
2007	KJ536104/07SDJN8/wheat	2007/5/10	China	KJ536104
2007	KJ536119/07SXTY3/wheat	2007/5/8	China	KJ536119
2007	KJ536120/07SXTY6/wheat	2007/5/8	China	KJ536120
2007	KJ536100/07NXYN2/wheat	2007/4/20	China	KJ536100
2007	KJ536101/07NXYN3/wheat	2007/4/20	China	KJ536101
2007	KJ536098/07NXYC3/wheat	2007/4/20	China	KJ536098
2007	KJ536099/07NXYC5/wheat	2007/4/20	China	KJ536099
2007	KJ536096/07NXYC14/wheat	2007/4/20	China	KJ536096
2007	KJ536097/07NXYC16/wheat	2007/4/20	China	KJ536097
2007	KJ536106/07SXDT2/wheat	2007/6/15	China	KJ536106
2007	KJ536107/07SXDT3/wheat	2007/6/15	China	KJ536107
2008	KJ536131/08YNKM8/wheat	2008/3/15	China	KJ536131
2008	KJ536130/08YNKM2/wheat	2008/3/15	China	KJ536130
2008	KJ536129/08YNKM10/wheat	2008/3/15	China	KJ536129
2008	KJ536141/08YNKM13/barley	2008/3/15	China	KJ536141

2008	KJ536127/08HNZD-3/wheat	2008/4/1	China	KJ536127
2008	KJ536128/08HNHYK-4/wheat	2008/4/2	China	KJ536128
2008	KJ536092/08HNZZ1/wheat	2008/3/30	China	KJ536092
2008	KJ536093/08HNZZ4/wheat	2008/3/30	China	KJ536093
2008	KJ536094/08HNZZ6/wheat	2008/3/30	China	KJ536094
2008	KJ536095/08HNZZ7/wheat	2008/3/30	China	KJ536095
2008	KJ536133/08XJWLMQ-3/wheat	2008/5/15	China	KJ536133
2008	KJ536134/08XJWLMQ-5/wheat	2008/5/15	China	KJ536134
2008	KJ536135/08XJWLMQ-12/wheat	2008/5/15	China	KJ536135
2008	KJ536150/08QHXN-11/ highland barley	2008/6/15	China	KJ536150
2008	KJ536149/08QHXN-17/ highland barley	2008/6/15	China	KJ536149
2008	KJ536148/08QHXN-26/ highland barley	2008/6/15	China	KJ536148
2008	KJ536147/08QHXN-29/ highland barley	2008/6/15	China	KJ536147
2008	KJ536132/08HBSJZ-1/wheat	2008/4/17	China	KJ536132
2009	KJ536138/09SXHC-1/wheat	2008/5/13	China	KJ536138
2009	KJ536139/09SXHC-11/wheat	2008/5/13	China	KJ536139
2009	KJ536140/09SXHC-27/wheat	2008/5/13	China	KJ536140
2009	KJ536137/09GSGG-5/wheat	2008/4/15	China	KJ536137
2009	KJ536136/09GSGG-8/wheat	2008/4/15	China	KJ536136
2010	JQ647501/SXTY10-9/wheat	2010/5/8	China	JQ647501
2010	JQ647502/SXTY10-18/wheat	2010/5/8	China	JQ647502
2010	JQ647503/SXTY10-32/wheat	2010/5/8	China	JQ647503
2010	JQ647504/SXTY10-33/wheat	2010/5/8	China	JQ647504
2010	JQ647505/SXTY10-35/ wheat	2010/5/8	China	JQ647505
2010	JQ647506/SXTY10-39/ wheat	2010/5/8	China	JQ647506
2010	JQ647507/SXTY10-40/ wheat	2010/5/8	China	JQ647507
2010	JQ647486/SXDT10-2/ wheat	2010/5/8	China	JQ647486
2010	JQ647487/SXDT10-3/ wheat	2010/5/8	China	JQ647487
2010	JQ647470/HBSJZ10-10/ wheat	2010/4/17	China	JQ647470
2010	JQ647471/HBSJZ10-17/ wheat	2010/4/17	China	JQ647471
2010	JQ647472/HBSJZ10-18/ wheat	2010/4/17	China	JQ647472
2010	JQ647473/HBSJZ10-20/ wheat	2010/4/17	China	JQ647473
2010	JQ647474/HBSJZ10-21/ wheat	2010/4/17	China	JQ647474
2010	JQ647475/HBSJZ10-22/ wheat	2010/4/17	China	JQ647475
2010	JQ647476/HBSJZ10-23/ wheat	2010/4/17	China	JQ647476
2010	JQ647488/SXHC10-5/ wheat	2010/5/12	China	JQ647488
2010	JQ647489/SXHC10-6/ wheat	2010/5/12	China	JQ647489
2010	JQ647490/SXHC10-7/ wheat	2010/5/12	China	JQ647490
2010	JQ647491/SXHC10-8/ wheat	2010/5/12	China	JQ647491
2010	JQ647492/SXHC10-9/ wheat	2010/5/12	China	JQ647492
2010	JQ647493/SXHC10-10/ wheat	2010/5/12	China	JQ647493
2010	JQ647494/SXHC10-11/ wheat	2010/5/12	China	JQ647494
2010	JQ647495/SXHC10-12/ wheat	2010/5/12	China	JQ647495
2010	JQ647496/SXHC10-14/ wheat	2010/5/12	China	JQ647496

2010	JQ647497/SXHC10-15/ wheat	2010/5/12	China	JQ647497
2010	JQ647498SXHC10-16/ wheat	2010/5/12	China	JQ647498
2010	JQ647499/SXHC10-17/ wheat	2010/5/12	China	JQ647499
2010	JQ647500/SXHC10-18/ wheat	2010/5/12	China	JQ647500
2010	JQ647483/SCCD10-2/ wheat	2010/3/12	China	JQ647483
2010	JQ647484/SCCD10-3/ wheat	2010/3/12	China	JQ647484
2010	JQ647485/SCCD10-6/ wheat	2010/3/12	China	JQ647485
2010	JQ647477/QHLD10-1/ wheat	2010/6/15	China	JQ647477
2010	JQ647478/QHLD10-8/ wheat	2010/6/15	China	JQ647478
2010	JQ647479/QHLD10-9/ wheat	2010/6/15	China	JQ647479
2010	JQ647480/QHLD10-15/ wheat	2010/6/15	China	JQ647480
2010	JQ647481/QHLD10-18/ wheat	2010/6/15	China	JQ647481
2010	JQ647482/QHLD10-19/ wheat	2010/6/15	China	JQ647482
2010	JQ647508/XZ10-2// wheat	2010/6/25	China	JQ647508
2010	JQ647467/Hungary-MO10-1/ wheat	2010/4/30	China	JQ647467
2010	JQ647468/Hungary-MO10-2/ wheat	2010/4/30	China	JQ647468
2010	JQ647469/Hungary-MO10-3/ wheat	2010/4/30	China	JQ647469
2010	JQ647455/Hungary-kp10-1/ wheat	2010/4/30	China	JQ647455
2010	JQ647456/Hungary-kp10-3/ wheat	2010/4/30	China	JQ647456
2010	JQ647457/Hungary-kp10-4/ wheat	2010/4/30	China	JQ647457
2010	JQ647458/Hungary-kp10-5/ wheat	2010/4/30	China	JQ647458
2010	JQ647459/Hungary-kp10-6/ wheat	2010/4/30	China	JQ647459
2010	JQ647460/Hungary-kp10-8/ wheat	2010/4/30	China	JQ647460
2010	JQ647461/Hungary-kp10-9/ wheat	2010/4/30	China	JQ647461
2010	JQ647462/Hungary-kp10-10/ wheat	2010/4/30	China	JQ647462
2010	JQ647463/Hungary-kp10-11/ wheat	2010/4/30	China	JQ647463
2010	JQ647464/Hungary-kp10-13/ wheat	2010/4/30	China	JQ647464
2010	JQ647465/Hungary-kp10-15/ wheat	2010/4/30	China	JQ647465
2010	JQ647466/Hungary-kp10-16/ wheat	2010/4/30	China	JQ647466
2010	JQ647454/Hungary-pc10-1/ wheat	2010/4/30	China	JQ647454
2008	KJ473695/Spelt8/Spelt	2008/5/15	Germany	KJ473695
2008	KJ473696/Spelt9/Spelt	2008/5/15	Germany	KJ473696
2008	KJ473697/Winter rye 49/Rye	2008/5/15	Germany	KJ473697
2008	KJ473698/Winter rye 101/Rye	2008/5/15	Germany	KJ473698
2008	KJ473699/Winter rye 102/Rye	2008/5/15	Germany	KJ473699
2008	KJ473700/Winter wheat 110/wheat	2008/5/15	Germany	KJ473700
2008	KJ473701/Winter wheat 111/wheat	2008/5/15	Germany	KJ473701
2008	KJ473702/Triticale 117/ Triticale	2008/5/15	Germany	KJ473702
2008	KJ473703/Triticale 118/ Triticale	2008/5/15	Germany	KJ473703
2008	KJ473704/Winter wheat 120/wheat	2008/5/15	Germany	KJ473704
2008	KJ473705/Winter wheat 121/wheat	2008/5/15	Germany	KJ473705
2008	KJ473706/Rye4 6/Rye	2008/5/15	Germany	KJ473706
2008	KJ473707/Rye9 3/Rye	2008/5/15	Germany	KJ473707

Supplementary Table S3. Genomic sequences of *Wheat dwarf virus* and *Oat dwarf virus* from GenBank used in this study.

Accession No.	Hosts	Countries	Isolates	Collection date
AM040733	Wheat	Hungary	WDV-[HU-F]	2005/6/28
AM040732	Wheat	Hungary	WDV-[HU-B]	2005/6/28
AJ311031	Wheat	Sweden	WDV-[Enkopping]	2006/11/14
X82104	Wheat	France	WDV-[FR]	2005/4/18
AM296018	Barley	Germany	BDV-[SxA18]	2008/1/25
AM296019	Barley	Czech Rep.	BDV-[Cz19]	2008/1/25
AM296020	Barley	Germany	BDV-[McP20]	2008/1/25
AM296021	Wheat	Germany	WDV-[BB21]	2008/1/25
AM296022	Wheat	Germany	WDV-[SxA22]	2008/1/25
AM296023	Wheat	Germany	WDV-[SxA23]	2008/1/25
AM296024	Barley	Germany	WDV-[SxA24]	2008/1/25
AM296025	Oat	Germany	ODV-[SxA25]	2008/1/25
AM411651	Barley	Germany	BDV-[BaW1]	2007/6/18
AM411652	Barley	Germany	BDV-[BaW2]	2007/6/18
X02869	Wheat	Sweden	WDV-[SE]	2005/7/1
AJ783960	Barley	Turkey	BDV-[TR2]	2006/11/14
AM491490	Wheat	Sweden	WDV-[SE2]	2008/11/1
FJ546178	Barley	Czech Rep.	WDV-[CZ6482]	2009/4/28
FJ546179	Barley	Czech Rep.	WDV-[CZ1800]	2009/4/28
FJ546180	Barley	Czech Rep.	WDV-[CZ11105]	2009/4/28
FJ546181	Barley	Czech Rep.	WDV-[CZ11229]	2009/4/28
FJ546191	Wheat	Czech Rep.	WDV-[CZ1841]	2009/4/28
FJ546192	Barley	Czech Rep.	WDV-[CZ7040]	2009/4/28
FJ546193	Barley	Czech Rep.	WDV-[CZWDV-B]	2009/4/28
FJ546190	Wheat	Czech Rep.	WDV-[CZ6239]	2009/4/28
FJ546189	Wheat	Czech Rep.	WDV-[CZ6217]	2009/4/28
FJ546188	Wheat	Czech Rep.	WDV-[CZ1561]	2009/4/28
FM999832	Barley	Hungary	WDV-[D01]	2009/2/19
FM999833	Barley	Hungary	WDV-[HE]	2009/2/19
FM210034	Barley	Hungary	WDV-[H07]	2008/9/13
AM989927	Barley	Bulgaria	WDV-[Bg17]	2008/5/7
FN806784	Wheat	Ukraine	WDV-[Uk-Miron]	2010/4/21
FN806785	Wheat	Hungary	WDV-[HU2-Marton]	2010/4/21
FN806786	Wheat	Hungary	WDV-HU-Pula	2010/4/21
FN806787	Wheat	Ukraine	WDV-Uk-Odessa	2010/4/21
AM747816	Barley	Hungary	WDV-HU	2009/3/12
AM942044	Barley	Germany	WDV-SxA57	2008/12/20
AM942045	Barley	Germany	WDV-SxA36	2008/12/20
AM921649	Barley	Germany	WDV-SABg12-11	2008/12/14
HG422310	Wheat	Germany	Leutewitz1	2013/5

HG422316	Wheat	Germany	Aschersleben1	2013/10/11
HG422317	Wheat	Germany	Alsleben3	2013/10/11
HG422318	Wheat	Germany	Alsleben9	2013/10/11
HF968637	Wheat	France	68WDV_1	2013/10/11
JN791095	Wheat	Iran	Shahrekord	2012/2/4
HF968633	Wheat	UK	UK193WDV1	2013/10/11
HG422314	Wheat	Germany	Aschersleben3	2013/10/11
HG422315	Wheat	Germany	Krostitz1	2013/10/11
HF968650	Barley	Germany	DE45BDV	2013/10/11
AM922260	Barely	Germany	SA45EcoFL38	2013/4/4
AM922261	Barely	Germany	SA43EcoFL23	2013/4/4
AM922262	Barely	Germany	SA12EcoFL2	2013/4/4
HF968639	Barely	Spain	ES1BDV_1	2013/10/11
HF968641	Barely	Spain	ES2BDV_1	2013/10/11
HF968644	Barely	Spain	ES3BDV_1	2013/10/11
AM989927	Barely	Bulgaria	Bg17	2008/5/7
HF968646	Barely	Austria	AU196BDV1	2013/10/11
FJ620684	Barely	Iran		2013/3/15
JN791096	Barely	Iran	Bavanat	2012/2/4

Supplementary Table S4. Selection pressure of the different proteins of *Wheat dwarf virus*.

**Group**	**Protein**	***N****	d_N	d_S	d_N/d_S
**WDV-wheat-specific group**	**CP**	184	0.00479	0.04489	0.10671
	**MP**	184	0.00601	0.03359	0.17892
	**Rep**	184	0.00466	0.03306	0.14096
	**Rep A**	184	0.00550	0.02662	0.20661
**WDV-barley-specific group**	**CP**	24	0.00460	0.04461	0.10312
	**MP**	24	0.00917	0.00762	1.20341
	**Rep**	24	0.00610	0.03146	0.19340
	**Rep A**	24	0.00751	0.04284	0.17530
**WDV**	**CP**	208	0.02212	0.13123	0.16856
	**MP**	208	0.02622	0.08440	0.31066
	**Rep**	208	0.01029	0.09712	0.10595
	**Rep A**	208	0.01450	0.07931	0.18283

***** *N*: isolate numbers: d_N: nonsynonymous substitution rate; d_S: synonymous substitution rate; Values of $d_N/d_S < 1$, $= 1$ or > 1 indicate negative selection, neutral evolution and positive selection, respectively.

Supplementary Table S5. Selection analysis and coevolutionary site residues of intra and inter protein in WDV and BDV

A．WDV-CP； B：WDV-MP； C：WDV-Rep； D：WDV-Rep A；E：BDV-CP；F：BDV-Rep； G：BDV-Rep A

A.

Coevolutionary sites of inter-protein of CP and Rep	Coevolutionary sites of inter-protein of CP and Rep A	Coevolutionary sites of intra-protein	Codon#	Triplet	Syn (*s*)	Nonsyn (*n*)	Syn sites (*S*)	Nonsyn sites (*N*)	d_S	d_N	$d_N - d_S$	*P*-value	Normalized $d_N - d_S$
			1	ATG	0	0	0	3	0	0	0	0	0
			2	GTG	0	0	1	2	0	0	0	0	0
			3	ACC	0	0	1	2	0	0	0	0	0
			4	AAC	0	0	0.604154	2.39585	0	0	0	0	0
			5	AAG	0	0	0.456094	2.16974	0	0	0	0	0
			6	GAC	0	0	0.604154	2.39585	0	0	0	0	0
			7	TCC	0	0	1	2	0	0	0	0	0
			8	CGA	0	0	1.1627	1.23315	0	0	0	0	0
			9	GGT	0	0	1	2	0	0	0	0	0
			10	AAG	0	0	0.456094	2.16974	0	0	0	0	0
			11	GGT	1	0	1	2	1	0	-1	1	-2.32188
			12	AAG	1	0	0.450855	2.17498	2.218	0	-2.218	1	-5.14995
			13	CGG	2	0	1.06134	1.93866	1.8844	0	-1.8844	1	-4.37536
			14	AAG	0	0	0.456094	2.16974	0	0	0	0	0
15	15		15	ATG	1.5	3.5	0.628247	2.37175	2.3876	1.4757	-0.9119	0.8262	-2.11731
			16	GAA	1	1	0.446725	2.35023	2.2385	0.4255	-1.813	0.9745	-4.20963
			17	GAA	1	0	0.434894	2.34446	2.2994	0	-2.2994	1	-5.33895
			18	GGT	3	1	1	1.95935	3	0.5104	-2.4896	0.987	-5.78061
			19	GAA	0	1	0.44129	2.35567	0	0.4245	0.42451	0.8422	0.985657

			20	TCC	9	0	1	1.97853	9	0	-9	1	-20.8969
			21	AGC	1	1	0.604865	2.39513	1.6533	0.4175	-1.2358	0.9593	-2.86926
			22	GGA	0	1	0.999443	1.77968	0	0.5619	0.5619	0.6404	1.30466
			23	AGG	1	0	0.648749	2.30141	1.5414	0	-1.5414	1	-3.57901
			24	TGG	0	0	0	2.08781	0	0	0	0	0
			25	AAG	0	0	0.456094	2.16974	0	0	0	0	0
			26	GGG	0	0	1	2	0	0	0	0	0
			27	GCT	0	0	1	2	0	0	0	0	0
			28	GTG	0	0	1	2	0	0	0	0	0
			29	TAT	0	1	0.575512	2.0019	0	0.4995	0.49953	0.7767	1.15984
			30	AAG	0	0	0.456094	2.16974	0	0	0	0	0
			31	CGG	0	0	1.1627	1.8373	0	0	0	0	0
			32	CGT	0	0	1	2	0	0	0	0	0
			33	AAA	0	0	0.429655	2.19618	0	0	0	0	0
			34	CAG	0	0	0.456094	1.93975	0	0	0	0	0
			35	GCG	1	4	1	2	1	2	1	0.4609	2.32188
			36	TAT	3	0	0.584584	2	5.1319	0	-5.1319	1	-11.9155
			37	AAG	0	0	0.456094	2.16974	0	0	0	0	0
			38	GTA	0	0	1	2	0	0	0	0	0
			39	GTA	0	2	1.00387	1.99613	0	1.0019	1.00194	0.4427	2.32638
			40	CCT	1	0	1	2	1	0	-1	1	-2.32188
			41	GTG	0	0	1	2	0	0	0	0	0
			42	AAG	0	0	0.456094	2.16974	0	0	0	0	0
			43	CCC	3	0	1	2	3	0	-3	1	-6.96564
			44	CCA	1	0	1	2	1	0	-1	1	-2.32188

			45	GCT	0	1	0.998101	2.0019	0	0.4995	0.49953	0.6673	1.15984
			46	CTC	10	0	1.17778	1.82222	8.4906	0	-8.4906	1	-19.714
			47	TGC	1	0	0.595982	2.20988	1.6779	0	-1.6779	1	-3.89589
			48	GTA	2	0	1	2	2	0	-2	1	-4.64376
			49	TTC	2	3	0.598854	2.36197	3.3397	1.2701	-2.0696	0.9403	-4.80532
			50	CGC	1	0	1.04642	1.95358	0.9556	0	-0.9556	1	-2.21888
			51	TAC	0	0	0.604154	2	0	0	0	0	0
			52	AAC	2	0	0.603941	2.39606	3.3116	0	-3.3116	1	-7.6891
			53	TGG	0	0	0	2.08781	0	0	0	0	0
			54	TTG	1	0	1.07728	1.6716	0.9283	0	-0.9283	1	-2.15532
			55	AAT	0	0	0.575512	2.42449	0	0	0	0	0
			56	AGC	1	3	0.635689	2.35998	1.5731	1.2712	-0.3019	0.8002	-0.700977
			57	GAC	1	0	0.604026	2.39597	1.6556	0	-1.6556	1	-3.844
			58	AGG	0	1	0.636621	2.33353	0	0.4285	0.42854	0.7857	0.995007
			59	ACC	0	0	1	2	0	0	0	0	0
			60	AAT	2	0	0.583942	2.41606	3.425	0	-3.425	1	-7.95243
			61	GTT	2	2	0.87906	2.12094	2.2752	0.943	-1.3322	0.9215	-3.09316
			62	GTT	1	0	1	2	1	0	-1	1	-2.32188
			63	GTG	0	1	1	2	0	0.5	0.5	0.6667	1.16094
			64	GGT	0	0	1	2	0	0	0	0	0
			65	AAT	1	0	0.583684	2.41632	1.7133	0	-1.7133	1	-3.97797
			66	ACA	1	0	1	2	1	0	-1	1	-2.32188
			67	CCC	1	1	1	2	1	0.5	-0.5	0.8889	-1.16094
			68	CGG	1	0	1.1627	1.66493	0.8601	0	-0.8601	1	-1.99698
			69	GTC	3	0	1	2	3	0	-3	1	-6.96564

			70	GAT	0	0	0.575512	2.42449	0	0	0	0	0
			71	CTG	1	0	1.6033	1.39629	0.6237	0	-0.6237	1	-1.44819
			72	ATT	0	0	0.848405	2.1516	0	0	0	0	0
			73	ACC	1	0	1	2	1	0	-1	1	-2.32188
			74	TGT	1	0	0.583684	2.17486	1.7133	0	-1.7133	1	-3.97797
			75	TTT	1	1	0.580974	2.41903	1.7213	0.4134	-1.3079	0.9625	-3.03669
			76	GCT	2	0	1	2	2	0	-2	1	-4.64376
			77	CAG	1	2	0.611081	1.97299	1.6364	1.0137	-0.6228	0.8587	-1.44596
			78	GGT	0	0	1	2	0	0	0	0	0
			79	AAG	0	0	0.456094	2.16974	0	0	0	0	0
			80	GCC	0	0	1	2	0	0	0	0	0
			81	GAT	0	0	0.575512	2.42449	0	0	0	0	0
			82	AAT	1	0	0.577797	2.4222	1.7307	0	-1.7307	1	-4.0185
			83	AAT	0	0	0.575512	2.42449	0	0	0	0	0
			84	CGG	1	0	1.16197	1.83803	0.8606	0	-0.8606	1	-1.99823
			85	CAT	0	0	0.575512	2.42449	0	0	0	0	0
			86	ACA	0	0	1	2	0	0	0	0	0
			87	AAT	0	0	0.575512	2.42449	0	0	0	0	0
			88	CAG	2	0	0.455937	1.93991	4.3866	0	-4.3866	1	-10.1851
			89	ACC	2	0	1	2	2	0	-2	1	-4.64376
			90	GTC	0	0	1	2	0	0	0	0	0
			91	CTA	2	0	1.55507	1.4443	1.2861	0	-1.2861	1	-2.98621
			92	TAC	0	0	0.604154	2	0	0	0	0	0
			93	AAA	1	0	0.437198	2.18863	2.2873	0	-2.2873	1	-5.31081
			94	TTT	1	0	0.583684	2.41632	1.7133	0	-1.7133	1	-3.97797

			95	AAC	0	5	0.604154	2.39585	0	2.087	2.08695	0.3249	4.84564
			96	ATA	0	0	0.570345	2.42965	0	0	0	0	0
			97	CAG	0	0	0.456094	1.93975	0	0	0	0	0
			98	GGT	1	0	1	2	1	0	-1	1	-2.32188
			99	ACC	0	0	1	2	0	0	0	0	0
			100	TGC	1	1	0.595982	2.21037	1.6779	0.4524	-1.2255	0.9549	-2.84544
			101	TAT	2	0	0.583727	2	3.4263	0	-3.4263	1	-7.95536
			102	ATG	0	1	0.285316	2.71468	0	0.3684	0.36837	0.9049	0.855303
			103	TCC	1	1	1	1.96656	1	0.5085	-0.4915	0.8864	-1.1412
			104	GAT	1	0	0.577797	2.4222	1.7307	0	-1.7307	1	-4.0185
			105	GCA	0	1	1	2	0	0.5	0.5	0.6667	1.16094
			106	TCA	1	1	1	1.60662	1	0.6224	-0.3776	0.8528	-0.876685
			107	GCT	0	1	1	2	0	0.5	0.5	0.6667	1.16094
108	108		108	CCG	0	3	0.826499	2.1735	0	1.3803	1.38026	0.3803	3.2048
109	109		109	TTC	1	2	0.605221	2.36194	1.6523	0.8468	-0.8055	0.8922	-1.87033
			110	ATC	2	1	0.752454	2.24755	2.658	0.4449	-2.213	0.9842	-5.13841
			111	GGT	1	0	1	2	1	0	-1	1	-2.32188
			112	CCA	1	0	1	2	1	0	-1	1	-2.32188
			113	GTC	1	1	0.917803	2.0822	1.0896	0.4803	-0.6093	0.9064	-1.41471
			114	CGC	1	0	1.04642	1.95358	0.9556	0	-0.9556	1	-2.21888
			115	CTC	2	1	1	2	2	0.5	-1.5	0.963	-3.48282
			116	TAC	2	0	0.595598	2	3.358	0	-3.358	1	-7.7968
			117	CAC	1	0	0.604112	2.39589	1.6553	0	-1.6553	1	-3.84346
			118	TGG	0	0	0	2.08781	0	0	0	0	0
			119	TTA	1	0	1.01271	1.60605	0.9874	0	-0.9874	1	-2.29274

			120	GTC	0	0	1	2	0	0	0	0	0
			121	TAT	0	0	0.575512	2	0	0	0	0	0
			122	GAT	0	0	0.575512	2.42449	0	0	0	0	0
			123	GCA	0	0	1	2	0	0	0	0	0
			124	GAG	0	1	0.458532	2.32181	0	0.4307	0.4307	0.8351	1.00003
			125	CTG	1	1	1.0018	1.9982	0.9982	0.5005	-0.4978	0.8885	-1.15572
			126	AAA	0	0	0.429655	2.19618	0	0	0	0	0
			127	CAG	0	0	0.456094	1.93975	0	0	0	0	0
			128	GCA	12	0	1	2	12	0	-12	1	-27.8625
			129	ATG	0	1	0.285316	2.71468	0	0.3684	0.36837	0.9049	0.855303
			130	CCA	1	0	1	2	1	0	-1	1	-2.32188
			131	GAC	0	1	0.603632	2.39571	0	0.4174	0.41741	0.7987	0.969183
132	132	132	132	GCC	0	2	1	2	0	1	1	0.4444	2.32188
			133	ACT	0	0	1	2	0	0	0	0	0
			134	GAC	0	1	0.605926	2.39407	0	0.4177	0.4177	0.798	0.969844
			135	ATC	0	0	0.766852	2.23315	0	0	0	0	0
			136	TTT	0	0	0.575512	2.42449	0	0	0	0	0
			137	ACG	0	0	1	2	0	0	0	0	0
			138	ATG	0	0	0	3	0	0	0	0	0
			139	CCT	4	0	1	2	4	0	-4	1	-9.28752
			140	TGG	0	0	0	2.08781	0	0	0	0	0
			141	AAT	0	0	0.575512	2.42449	0	0	0	0	0
			142	CTG	0	0	1.60415	1.39585	0	0	0	0	0
			143	CTG	3	0	1.59561	1.40032	1.8802	0	-1.8802	1	-4.3655
			144	CCG	0	0	1	2	0	0	0	0	0

			145	AGT	0	0	0.575512	2.42449	0	0	0	0	0
146	146	146	146	ACG	1	2	0.998511	1.95507	1.0015	1.023	0.02149	0.7344	0.0498985
			147	TGG	0	0	0	2.08781	0	0	0	0	0
			148	ACT	4	0	1	2	4	0	-4	1	-9.28752
			149	GTG	0	0	1	2	0	0	0	0	0
			150	CAA	2	0	0.431882	1.96396	4.6309	0	-4.6309	1	-10.7524
			151	CGT	2	0	1.0057	1.9943	1.9887	0	-1.9887	1	-4.61743
			152	GCT	0	0	1	2	0	0	0	0	0
			153	TGG	0	0	0	2.08781	0	0	0	0	0
			154	TCG	0	1	1	1.84508	0	0.542	0.54198	0.6485	1.25842
			155	CAT	0	1	0.574859	2.42244	0	0.4128	0.41281	0.8082	0.95849
			156	CGA	1	0	1.1627	1.40552	0.8601	0	-0.8601	1	-1.99698
			157	TTC	0	0	0.604154	2.39585	0	0	0	0	0
			158	GTG	0	0	1	2	0	0	0	0	0
			159	GTG	0	0	1	2	0	0	0	0	0
			160	AAA	1	0	0.437198	2.18863	2.2873	0	-2.2873	1	-5.31081
			161	AGG	0	0	0.652271	2.34773	0	0	0	0	0
			162	AAG	0	0	0.456094	2.16974	0	0	0	0	0
			163	TGG	0	0	0	2.08781	0	0	0	0	0
			164	ACC	0	2	0.887059	2.07325	0	0.9647	0.96467	0.4905	2.23985
			165	GCG	0	1	1	2	0	0.5	0.5	0.6667	1.16094
			166	AAC	0	1	0.605927	2.39407	0	0.4177	0.4177	0.798	0.969845
			167	CTT	1	0	1.0027	1.9973	0.9973	0	-0.9973	1	-2.31563
			168	GTT	1	0	1	2	1	0	-1	1	-2.32188
			169	ACT	1	1	1	2	1	0.5	-0.5	0.8889	-1.16094

			170	GAT	0	0	0.575512	2.42449	0	0	0	0	0
			171	GGA	1	0	1	1.79696	1	0	-1	1	-2.32188
			172	CGG	2	1	1.00141	1.96693	1.9972	0.5084	-1.4888	0.9616	-3.45674
			173	AAG	0	1	0.45668	2.17027	0	0.4608	0.46077	0.8262	1.06986
			174	GTC	2	0	1	2	2	0	-2	1	-4.64376
			175	GGG	1	0	1	1.9824	1	0	-1	1	-2.32188
			176	TCT	5	0	1	1.99421	5	0	-5	1	-11.6094
178	178		177	AAG	1	1	0.456599	2.1696	2.1901	0.4609	-1.7292	0.9698	-4.01497
			178	ACC	1	3	1	1.98702	1	1.5098	0.5098	0.59	1.18369
			179	GTT	1	0	1	2	1	0	-1	1	-2.32188
			180	GAC	2	0	0.601784	2.39822	3.3235	0	-3.3235	1	-7.71665
			181	CAA	1.5	1.5	0.598677	1.99452	2.5055	0.7521	-1.7535	0.9262	-4.07134
			182	CGC	2	0	1.00049	1.99771	1.999	0	-1.999	1	-4.6415
			183	TAC	0	1	0.604154	2.00118	0	0.4997	0.49971	0.7681	1.16026
			184	AAC	0	0	0.604154	2.39585	0	0	0	0	0
			185	TGG	0	2	0.197351	2.0784	0	0.9623	0.96228	0.8341	2.23429
			186	GTA	0	0	1	2	0	0	0	0	0
			187	GTC	4	1	1	2	4	0.5	-3.5	0.9959	-8.12658
			188	GGC	3	0	1	2	3	0	-3	1	-6.96564
			189	AAG	1	0	0.456055	2.16978	2.1927	0	-2.1927	1	-5.09122
			190	AAT	0	1	0.575512	2.42449	0	0.4125	0.41246	0.8082	0.957678
			191	ATC	2	0	0.790606	2.20939	2.5297	0	-2.5297	1	-5.87367
			192	GTG	3	0	1	2	3	0	-3	1	-6.96564
			193	GAC	0	1	0.604154	2.3955	0	0.4174	0.41745	0.7986	0.969267
			194	GCA	6	0	1	2	6	0	-6	1	-13.9313

			195	AGT	4	5	0.632373	2.3633	6.3254	2.1157	-4.2097	0.9754	-9.7744
			196	AAG	0	0	0.456094	2.16974	0	0	0	0	0
			197	TTC	0	0	0.604154	2.39585	0	0	0	0	0
			198	TTC	1	1	0.604112	2.34947	1.6553	0.4256	-1.2297	0.9582	-2.8552
			199	AAA	0	0	0.429655	2.19618	0	0	0	0	0
			200	GGT	0	0	1	2	0	0	0	0	0
			201	TTG	1	0	1.03416	1.69416	0.967	0	-0.967	1	-2.24517
			202	CGT	2	0	1.04642	1.95358	1.9113	0	-1.9113	1	-4.43776
			203	GTC	0	0	1	2	0	0	0	0	0
			204	ACG	0	0	1	2	0	0	0	0	0
			205	ACG	0	1	1	2	0	0.5	0.5	0.6667	1.16094
			206	GAG	0	0	0.456094	2.32326	0	0	0	0	0
			207	TGG	0	0	0	2.08781	0	0	0	0	0
			208	ATG	0	0	0	3	0	0	0	0	0
			209	AAC	0	0	0.604154	2.39585	0	0	0	0	0
			210	ACG	0	0	1	2	0	0	0	0	0
			211	GGT	0	0	1	2	0	0	0	0	0
			212	GAC	1	1	0.603806	2.39587	1.6562	0.4174	-1.2388	0.9595	-2.87629
			213	GGC	3	1	0.998819	2.00118	3.0036	0.4997	-2.5038	0.9877	-5.81362
			214	AAG	0	0	0.456094	2.16974	0	0	0	0	0
			215	ATA	0	0	0.570345	2.42965	0	0	0	0	0
			216	GGC	0	0	1	2	0	0	0	0	0
			217	GAC	0	0	0.604154	2.39585	0	0	0	0	0
			218	ATT	0	0	0.848405	2.1516	0	0	0	0	0
			219	AAG	0	0	0.456094	2.16974	0	0	0	0	0

			220	AAG	2	0	0.453201	2.17263	4.4131	0	-4.4131	1	-10.2466
			221	GGA	2	0	1	1.84231	2	0	-2	1	-4.64376
			222	GCT	3	0	1	2	3	0	-3	1	-6.96564
			223	CTG	0	0	1.60415	1.39585	0	0	0	0	0
			224	TAT	0	0	0.575512	2	0	0	0	0	0
			225	CTT	5	0	1.0527	1.9473	4.7497	0	-4.7497	1	-11.0282
			226	ATT	2	0	0.767868	2.23213	2.6046	0	-2.6046	1	-6.0476
			227	AGC	1.5	1.5	0.63494	2.35511	2.3624	0.6369	-1.7255	0.9371	-4.00644
			228	AGT	0	1	0.575512	2.40272	0	0.4162	0.4162	0.8068	0.966355
			229	ACT	0	0	1	2	0	0	0	0	0
			230	CGT	1	1	0.97314	1.9909	1.0276	0.5023	-0.5253	0.8922	-1.21972
			231	GGT	1	0	1	1.99901	1	0	-1	1	-2.32188
			232	GGT	0	0	1	2	0	0	0	0	0
			233	GTT	3	0	1	2	3	0	-3	1	-6.96564
			234	ACT	0	1	0.966138	2.03386	0	0.4917	0.49168	0.678	1.14161
			235	GGT	1	0	1	1.99967	1	0	-1	1	-2.32188
			236	GAC	5	0	0.600709	2.39929	8.3235	0	-8.3235	1	-19.3262
			237	AGT	0	0	0.575512	2.42449	0	0	0	0	0
			238	GCC	0	1	1	2	0	0.5	0.5	0.6667	1.16094
			239	TCT	0	0	1	2	0	0	0	0	0
240	240		240	ACG	1	2	0.842652	2.15735	1.1867	0.9271	-0.2597	0.8076	-0.602914
			241	GCG	0	1	1	1.95358	0	0.5119	0.51188	0.6614	1.18853
			242	TTT	1	0	0.583684	2.41632	1.7133	0	-1.7133	1	-3.97797
			243	GAT	0	1	0.533897	2.40315	0	0.4161	0.41612	0.8182	0.966182
			244	GTT	0	0	1	2	0	0	0	0	0

			245	GTA	0	0	1	2	0	0	0	0	0
			246	TGT	0	0	0.575512	2.1516	0	0	0	0	0
			247	GCC	0	0	1	2	0	0	0	0	0
			248	TAT	0	0	0.575512	2	0	0	0	0	0
			249	ACG	0	0	1	2	0	0	0	0	0
			250	CAC	0	0	0.604154	2.39585	0	0	0	0	0
			251	GCG	0	0	1	2	0	0	0	0	0
			252	TGT	0	0	0.575512	2.1516	0	0	0	0	0
			253	TAT	3	0	0.579149	2	5.18	0	-5.18	1	-12.0274
			254	TTC	1	0	0.604026	2.39597	1.6556	0	-1.6556	1	-3.844
			255	AAA	0	0	0.429655	2.19618	0	0	0	0	0
			256	GCC	1	1	1	2	1	0.5	-0.5	0.8889	-1.16094
			257	ATC	4	0	0.7908	2.2092	5.0582	0	-5.0582	1	-11.7445
			258	GGC	2	0	1	2	2	0	-2	1	-4.64376
			259	ATT	0	0	0.848405	2.1516	0	0	0	0	0
			260	CAG	1	0	0.448551	1.94729	2.2294	0	-2.2294	1	-5.1764

B.

Coevolutionary sites of intra-protein	Codon#	Triplet	Syn (s)	Nonsyn (n)	Syn sites (S)	Nonsyn sites (N)	d_S	d_N	d_N - d_S	P-value	Normalized d_N - d_S
	1	ATG	0	0	0	3	0	0	0	0	0
	2	GAG	0	1	0.441159	2.23854	0	0.4467	0.44672	0.8354	1.1235
	3	CAG	1	3	0.494701	1.94741	2.0214	1.5405	-0.4809	0.8152	-1.2095
	4	GCA	0	2	1	2	0	1	1	0.4444	2.51499
	5	ATC	1.5	1.5	0.714051	2.27253	2.1007	0.6601	-1.4406	0.9211	-3.62318
	6	GCC	1	2	1	2	1	1	0	0.7407	0
	7	CCC	1	1	1	2	1	0.5	-0.5	0.8889	-1.2575
	8	CCG	0	1	1.00296	1.99704	0	0.5007	0.50074	0.6657	1.25936
	9	CTT	0	1	1	2	0	0.5	0.5	0.6667	1.2575
	10	CCT	0	1	1	2	0	0.5	0.5	0.6667	1.2575
	11	ATA	0	0	0.616289	2.38371	0	0	0	0	0
	12	CGG	0	0	1.22056	1.77944	0	0	0	0	0
13	13	GAC	1.5	4.5	0.744348	2.24583	2.0152	2.0037	-0.0115	0.6844	-0.0288515
	14	TAT	0	1	0.578713	2.13516	0	0.4683	0.46835	0.7868	1.17789
	15	CAA	0	0	0.383711	1.98149	0	0	0	0	0
	16	TAC	0	0	0.634804	2	0	0	0	0	0
	17	CAG	0	0	0.426628	1.93857	0	0	0	0	0
	18	ACC	0	1	1	2	0	0.5	0.5	0.6667	1.2575

	19	CCT	1	1	1	2	1	0.5	-0.5	0.8889	-1.2575
	20	TCC	0	0	1	2	0	0	0	0	0
21	21	ATT	0	5	0.664156	2.33584	0	2.1406	2.14055	0.2862	5.38347
	22	CCC	1	1	1	2	1	0.5	-0.5	0.8889	-1.2575
	23	GGT	3	0	1	1.94333	3	0	-3	1	-7.54497
	24	TCC	0	1	1	2	0	0.5	0.5	0.6667	1.2575
	25	TCC	0	1	0.998295	2.0017	0	0.4996	0.49957	0.6672	1.25642
	26	GAC	0	0	0.634804	2.3652	0	0	0	0	0
	27	TAC	0	0	0.634804	2	0	0	0	0	0
	28	GCC	0	0	1	2	0	0	0	0	0
	29	TGG	0	0	0	2.14674	0	0	0	0	0
	30	CGA	0	0	1.22056	1.14464	0	0	0	0	0
	31	ACA	1	1	1	2	1	0.5	-0.5	0.8889	-1.2575
	32	TTT	0	0	0.578713	2.42129	0	0	0	0	0
	33	GTG	0	0	1	2	0	0	0	0	0
	34	TTC	1	0	0.634543	2.36546	1.5759	0	-1.5759	1	-3.96347
	35	GTT	0	1	1	2	0	0.5	0.5	0.6667	1.2575
	36	ACC	2	1	1	1.97674	2	0.5059	-1.4941	0.9621	-3.75769
	37	TTC	3	0	0.632698	2.3673	4.7416	0	-4.7416	1	-11.9251
	38	GGT	2	0	1	2	2	0	-2	1	-5.02998
	39	TTG	1	0	1.00124	1.77012	0.9988	0	-0.9988	1	-2.51187
	40	CTA	6	0	1.43104	1.45055	4.1928	0	-4.1928	1	-10.5447
	41	ATA	0	0	0.616289	2.38371	0	0	0	0	0
	42	GCC	0	0	1	2	0	0	0	0	0
	43	GTA	0	0	1	2	0	0	0	0	0

	44	GGC	1	1	1.02009	1.97991	0.9803	0.5051	-0.4752	0.8844	-1.19521
	45	GTT	2	2	0.992058	2.00794	2.016	0.996	-1.02	0.8912	-2.56521
	46	GCT	2	3	1	2	2	1.5	-0.5	0.7901	-1.2575
	47	TGG	0	1	0.0142343	2.14164	0	0.4669	0.46693	0.9934	1.17433
	48	CTT	1	2	1.001	1.999	0.999	1.0005	0.0015	0.7403	0.0037602
	49	GCT	2	0	1	2	2	0	-2	1	-5.02998
	50	TAC	2	0	0.63428	2	3.1532	0	-3.1532	1	-7.93022
	51	ACT	1	0	1	2	1	0	-1	1	-2.51499
	52	CTG	1	0	1.63186	1.36717	0.6128	0	-0.6128	1	-1.54118
	53	TTT	1	0	0.59264	2.40736	1.6874	0	-1.6874	1	-4.24371
	54	CTG	3	0	1.38976	1.55853	2.1587	0	-2.1587	1	-5.42898
	55	AAA	2	0	0.388012	2.30052	5.1545	0	-5.1545	1	-12.9635
	56	GAT	0	0	0.578713	2.42129	0	0	0	0	0
	57	TTA	1	2	0.916681	1.7044	1.0909	1.1734	0.08254	0.7186	0.20759
	58	ATT	2	0	0.789556	2.21044	2.5331	0	-2.5331	1	-6.37065
	59	TTA	1	0	0.962624	1.61708	1.0388	0	-1.0388	1	-2.61264
	60	GTG	0	0	1	2	0	0	0	0	0
61	61	TGT	0	1	0.435029	2.19655	0	0.4553	0.45526	0.8347	1.14497
	62	AAG	0	0	0.426628	2.2619	0	0	0	0	0
	63	GCG	0	0	1	2	0	0	0	0	0
	64	AAG	0	1	0.426628	2.26039	0	0.4424	0.4424	0.8412	1.11263
	65	AAG	1	0	0.426429	2.2621	2.3451	0	-2.3451	1	-5.8978
	66	CAA	1	0	0.394366	1.97083	2.5357	0	-2.5357	1	-6.37729
	67	AGG	0	1	0.733962	2.26604	0	0.4413	0.4413	0.7553	1.10986
	68	AGG	0	0	0.731447	2.26855	0	0	0	0	0

	69	ACC	0	0	1	2	0	0	0	0	0
	70	GAG	0	0	0.426628	2.21923	0	0	0	0	0
	71	GAA	1	0	0.384111	2.26175	2.6034	0	-2.6034	1	-6.54757
	72	ATT	0	0	0.787002	2.213	0	0	0	0	0
	73	GGT	0	0	1	2	0	0	0	0	0
	74	TAC	0	0	0.634804	2	0	0	0	0	0
	75	GGG	1	2	0.993387	2.00331	1.0067	0.9983	-0.0083	0.7432	-0.0208939
	76	AAT	0	0	0.578713	2.42129	0	0	0	0	0
	77	ACA	0	0	1	2	0	0	0	0	0
	78	CCG	0	0	1	2	0	0	0	0	0
	79	GCC	0	0	1	2	0	0	0	0	0
	80	AGA	0	0	0.68853	2	0	0	0	0	0
81	81	TTA	0.5	3.5	0.981356	1.70682	0.5095	2.0506	1.5411	0.3494	3.87586
	82	AAT	0	0	0.578713	2.42129	0	0	0	0	0
	83	GGT	0	0	1	2	0	0	0	0	0
	84	GAC	0	0	0.634804	2.3652	0	0	0	0	0
	85	CAA	0	0	0.383711	1.98149	0	0	0	0	0
	86	CAA	0	0	0.383711	1.98149	0	0	0	0	0
	87	GGA	0	0	1	1.64586	0	0	0	0	0
	88	CTC	0	0	1	2	0	0	0	0	0
	89	CCG	0	0	1	2	0	0	0	0	0
	90	AGG	0	0	0.731447	2.26855	0	0	0	0	0

C.

Coevolutionary sites of inter-protein of Rep and Rep A	Coevolutionary sites of inter-protein of CP and Rep	Coevolutionary sites of intra-protein	Codon#	Triplet	Syn (*s*)	Nonsyn (*n*)	Syn sites (*S*)	Nonsyn sites (*N*)	d_S	d_N	d_N - d_S	*P*-value	Normalized d_N - d_S
			1	ATG	0	0	0	3	0	0	0	0	0
2	2	2	2	GCC	1	3	1	2	1	1.5	0.5	0.5926	1.08432
			3	TCT	0	1	0.997889	2.00211	0	0.4995	0.49947	0.6674	1.08317
			4	TCA	0	0	1	1.58458	0	0	0	0	0
			5	TCT	0	1	0.997889	2.00211	0	0.4995	0.49947	0.6674	1.08317
6	6	6	6	ACA	1	5	1	1.99871	1	2.5016	1.50161	0.3509	3.25645
			7	CCC	0	1	0.997848	2.00215	0	0.4995	0.49946	0.6674	1.08315
			8	AGG	0	1	0.914437	2.08556	0	0.4795	0.47949	0.6952	1.03983
			9	TTC	0	0	0.584579	2.41542	0	0	0	0	0
			10	CGA	7	0	1.06058	1.80502	6.6002	0	-6.6002	1	-14.3133
			11	GTC	2	0	1	2	2	0	-2	1	-4.33726
			12	TAT	0	0	0.592643	2	0	0	0	0	0
			13	TCC	2	0	1	1.90089	2	0	-2	1	-4.33726
			14	AAG	1	0	0.624746	2.11226	1.6007	0	-1.6007	1	-3.47122
			15	TAC	0	1	0.584579	2.00086	0	0.4998	0.49979	0.7739	1.08385
			16	CTC	1	1	1.00121	1.99879	0.9988	0.5003	-0.4985	0.8886	-1.08104
			17	TTT	1	0	0.590724	2.40928	1.6928	0	-1.6928	1	-3.67114

			18	CTA	0	1	1.58276	1.41724	0	0.7056	0.7056	0.4724	1.53018
			19	ACA	0	0	1	2	0	0	0	0	0
			20	TAT	0	0	0.592643	2	0	0	0	0	0
			21	CCT	0	0	1	2	0	0	0	0	0
			22	CAA	0	1	0.488808	1.92934	0	0.5183	0.51831	0.7979	1.12403
			23	TGT	1	0	0.590724	2.12459	1.6928	0	-1.6928	1	-3.67114
24	24	24	24	ACC	0	2	0.962558	2.03744	0	0.9816	0.98162	0.4612	2.12878
			25	CTT	0	0	1	2	0	0	0	0	0
			26	GAG	1	2	0.624746	2.23043	1.6007	0.8967	-0.704	0.8773	-1.52663
			27	CCA	1	1	1.00547	1.99453	0.9946	0.5014	-0.4932	0.8877	-1.06955
			28	CAG	0	2	0.666158	1.75396	0	1.1403	1.14028	0.5253	2.47284
29	29	29	29	TAC	1	3	0.588489	2.10012	1.6993	1.4285	-0.2708	0.7896	-0.587221
30		30	30	GCC	0	3	1.25578	1.4558	0	2.0607	2.06072	0.1548	4.46894
31		31	31	TTG	0	2	0.600211	2.38527	0	0.8385	0.83848	0.6383	1.81835
32	32	32	32	GAT	1	1	1	1.62761	1	0.6144	-0.3856	0.8552	-0.83623
			33	TCA	1	2	1.00512	1.99488	0.9949	1.0026	0.00766	0.7385	0.0166049
			34	CTT	1	1	0.99905	1.99931	1.001	0.5002	-0.5008	0.889	-1.086
			35	CGC	0.5	1.5	0.996276	2.0019	0.5019	0.7493	0.24742	0.6677	0.536563
			36	ACT	0	1	1	2	0	0.5	0.5	0.6667	1.08432
			37	CTC	1.5	2.5	1.25541	1.45517	1.1948	1.718	0.52319	0.5552	1.1346
			38	TTG	0	3	0.683419	2.31658	0	1.295	1.29501	0.4604	2.8084
39	39	39	39	AAC	0	3	0.49413	2.24677	0	1.3353	1.33525	0.5508	2.89566
			40	AAA	0	1	0.592643	2.00121	0	0.4997	0.4997	0.7715	1.08366
			41	TAT	0	0	0.667188	2.18982	0	0	0	0	0
			42	GAG	2	0	1	2	2	0	-2	1	-4.33726

			43	CCC	2	1	1.00121	1.99879	1.9976	0.5003	-1.4973	0.9628	-3.24705
			44	CTC	0	0	0.584579	2	0	0	0	0	0
			45	TAC	0	0	0.848056	2.15194	0	0	0	0	0
			46	ATC	1	0	1	2	1	0	-1	1	-2.16863
			47	GCT	1	4	1	2	1	2	1	0.4609	2.16863
48	48	48	48	GCT	0	0	1	2	0	0	0	0	0
			49	GTT	0	0	0.737004	2	0	0	0	0	0
			50	AGA	0	0	0.667188	2.18982	0	0	0	0	0
			51	GAG	1	0	1	2	1	0	-1	1	-2.16863
			52	CTC	0	0	0.584579	2.41542	0	0	0	0	0
			53	CAC	0	0	0.488808	2.3682	0	0	0	0	0
			54	GAA	0	1	0.592643	2.40736	0	0.4154	0.41539	0.8025	0.900834
			55	GAT	0	0	1	1.857	0	0	0	0	0
			56	GGA	0	0	1	1.58458	0	0	0	0	0
			57	TCA	0	0	1	2	0	0	0	0	0
			58	CCA	0	0	0.592643	2.40736	0	0	0	0	0
			59	CAT	3	0	1.57685	1.41893	1.9025	0	-1.9025	1	-4.12587
			60	CTG	0	0	0.584579	2.41542	0	0	0	0	0
			61	CAC	1	1	0.99976	2.00024	1.0002	0.4999	-0.5003	0.8889	-1.08497
			62	GTT	0	0	1	2	0	0	0	0	0
			63	CTC	0	0	1	2	0	0	0	0	0
			64	GTG	3	0	0.64871	1.76671	4.6246	0	-4.6246	1	-10.029
			65	CAG	0	0	0.584579	2.41542	0	0	0	0	0
			66	AAC	0	1	0.668991	2.06992	0	0.4831	0.48311	0.7557	1.04769
			67	AAG	0	0	1	2	0	0	0	0	0

			68	CTT	0	0	1	2	0	0	0	0	0
			69	CGT	0	0	1	2	0	0	0	0	0
			70	GCT	4	0	1	1.91479	4	0	-4	1	-8.67452
			71	TCC	0	0	0.848056	2.15194	0	0	0	0	0
			72	ATC	0	0	1	2	0	0	0	0	0
			73	ACC	0	0	0.592643	2.40736	0	0	0	0	0
			74	AAT	1	0	1	2	1	0	-1	1	-2.16863
			75	CCC	0	2	0.592643	2.40736	0	0.8308	0.83079	0.6439	1.80167
			76	AAT	3	0	1	2	3	0	-3	1	-6.50589
			77	GCC	2	1	1.04647	1.72808	1.9112	0.5787	-1.3325	0.9463	-2.88972
			78	TTA	0	1	0.584579	2.4124	0	0.4145	0.41453	0.8049	0.89895
			79	AAC	3	0	1.0006	1.9994	2.9982	0	-2.9982	1	-6.50196
			80	CTC	0	0	1	2	0	0	0	0	0
			81	CGT	0	0	0	3	0	0	0	0	0
			82	ATG	1	0	0.590724	2.40928	1.6928	0	-1.6928	1	-3.67114
			83	GAT	0	0	1	2	0	0	0	0	0
			84	ACA	1	0	1	2	1	0	-1	1	-2.16863
			85	TCT	0	0	1	2	0	0	0	0	0
			86	CCA	3	1	0.588864	2.40945	5.0946	0.415	-4.6795	0.9985	-10.1482
			87	TTC	1	2	0.958646	2.02757	1.0431	0.9864	-0.0567	0.757	-0.123039
			88	TCC	2	0	0.600268	2.39973	3.3318	0	-3.3318	1	-7.22554
			89	ATA	1	0	0.584739	2.41526	1.7102	0	-1.7102	1	-3.70871
			90	TTC	1	0	0.590724	2.40928	1.6928	0	-1.6928	1	-3.67114
			91	CAT	1	0	1	2	1	0	-1	1	-2.16863
			92	CCT	0	1	0.592643	2.4044	0	0.4159	0.41591	0.8023	0.901943

			93	AAT	1	2	0.590767	2.40869	1.6927	0.8303	-0.8624	0.8989	-1.8702
94		94	94	ATA	1	0	0.53125	1.88417	1.8824	0	-1.8824	1	-4.08213
			95	CAA	1	0	1	2	1	0	-1	1	-2.16863
			96	GCT	0	0	1	2	0	0	0	0	0
			97	GCC	0	0	0.488808	2.2482	0	0	0	0	0
			98	AAA	3	1	0.587441	2.41256	5.1069	0.4145	-4.6924	0.9985	-10.1761
			99	GAC	0	1	0.587168	2.151	0	0.4649	0.4649	0.7856	1.0082
			100	TGC	1	1	0.584788	2.41521	1.71	0.414	-1.296	0.962	-2.8105
			101	AAC	1	1	0.496227	1.92344	2.0152	0.5199	-1.4953	0.9579	-3.24276
			102	CAA	1	0	1	2	1	0	-1	1	-2.16863
			103	GTT	0	0	1	2	0	0	0	0	0
			104	CGT	0	0	0.592643	2.40736	0	0	0	0	0
			105	GAT	2	0	0.584733	2	3.4204	0	-3.4204	1	-7.4175
			106	TAC	0	1	0.848528	2.15147	0	0.4648	0.4648	0.7172	1.00798
			107	ATC	0	1	1	2	0	0.5	0.5	0.6667	1.08432
			108	ACG	3	1	0.643128	2.09442	4.6647	0.4775	-4.1872	0.997	-9.08058
			109	AAG	1	0	0.666081	2.19092	1.5013	0	-1.5013	1	-3.25581
			110	GAG	0	0	1	2	0	0	0	0	0
			111	GTT	0	0	0.584579	2.41542	0	0	0	0	0
			112	GAC	0	0	1	2	0	0	0	0	0
			113	TCC	0	0	0.592643	2.40736	0	0	0	0	0
			114	GAT	1	6	1.0006	1.9994	0.9994	3.0009	2.00151	0.263	4.34054
115	115	115	115	GTA	0	0	0.584579	2.41542	0	0	0	0	0
			116	AAC	1	0	1	2	1	0	-1	1	-2.16863
			117	ACA	0	0	1	2	0	0	0	0	0

			118	GCT	1	0	0.665895	2.19111	1.5017	0	-1.5017	1	-3.25671
			119	GAG	0	0	0	1.66562	0	0	0	0	0
			120	TGG	0	0	1	1.857	0	0	0	0	0
			121	GGA	0	0	1	2	0	0	0	0	0
			122	ACA	0	0	0.584579	2.41542	0	0	0	0	0
			123	TTC	1.5	1.5	0.933078	2.06692	1.6076	0.7257	-0.8819	0.8699	-1.91244
			124	GTG	4	1	1	2	4	0.5	-3.5	0.9959	-7.59021
			125	GCT	0	1	1	2	0	0.5	0.5	0.6667	1.08432
			126	GTT	1	1	1	1.64081	1	0.6095	-0.3905	0.8566	-0.846947
			127	TCA	1	0	1	2	1	0	-1	1	-2.16863
			128	ACT	1	1	1	2	1	0.5	-0.5	0.8889	-1.08432
			129	CCA	0	0	1	2	0	0	0	0	0
			130	GGT	0	0	1	2	0	0	0	0	0
			131	CGT	1	0	0.489177	2.24783	2.0443	0	-2.0443	1	-4.43322
			132	AAA	0	1	0.58587	2.41413	0	0.4142	0.41423	0.8047	0.898307
			133	GAC	0	0	1	2	0	0	0	0	0
			134	CGT	4	0	0.591559	2.40844	6.7618	0	-6.7618	1	-14.6638
			135	GAT	2	0	1	2	2	0	-2	1	-4.33726
			136	GCG	0	0	0.592643	2.40736	0	0	0	0	0
			137	GAT	0	0	0	3	0	0	0	0	0
			138	ATG	0	0	0.488808	2.2482	0	0	0	0	0
			139	AAA	1	0	0.657406	1.75802	1.5211	0	-1.5211	1	-3.29877
			140	CAG	0	0	0.848056	2.15194	0	0	0	0	0
			141	ATC	1	0	0.880311	2.11969	1.136	0	-1.136	1	-2.46348
			142	ATT	1	0	0.506917	2.35009	1.9727	0	-1.9727	1	-4.27808

			143	GAA	5	0	1	1.95697	5	0	-5	1	-10.8432
			144	TCT	0	2	0.612062	2.38523	0	0.8385	0.8385	0.6333	1.81839
			145	AGT	0	0	1	2	0	0	0	0	0
			146	TCC	1	0	1	2	1	0	-1	1	-2.16863
			147	TCT	4	0	1.00027	1.99973	3.9989	0	-3.9989	1	-8.67216
			148	CGC	1	1	0.666634	2.18798	1.5001	0.457	-1.043	0.9455	-2.26195
			149	GAG	0	0	0.488808	2.3682	0	0	0	0	0
			150	GAA	2	0	0.585423	2.41458	3.4163	0	-3.4163	1	-7.40877
			151	TTC	0	0	1	2	0	0	0	0	0
			152	CTC	1	0	0.584621	2.41538	1.7105	0	-1.7105	1	-3.70946
			153	AGC	0	0	0	3	0	0	0	0	0
			154	ATG	1	0	1	2	1	0	-1	1	-2.16863
			155	GTT	0	0	0.584579	2.15194	0	0	0	0	0
			156	TGC	0	3	0.592643	2.40736	0	1.2462	1.24618	0.5167	2.7025
157	157	157	157	AAT	1	0	1.00055	1.99825	0.9995	0	-0.9995	1	-2.16745
			158	CGT	1	1	0.595157	2.40484	1.6802	0.4158	-1.2644	0.9606	-2.74202
			159	TTT	1	0	1	2	1	0	-1	1	-2.16863
			160	CCG	0	0	0.592643	2.40736	0	0	0	0	0
			161	TTT	0	0	0.488808	2.3682	0	0	0	0	0
			162	GAA	0	0	0	1.66562	0	0	0	0	0
			163	TGG	3	0	1	1.94118	3	0	-3	1	-6.50589
			164	TCT	4	0	0.766646	2.23335	5.2175	0	-5.2175	1	-11.3149
			165	ATC	0	0	1	2	0	0	0	0	0
			166	CGT	4	0	1.01334	1.98666	3.9473	0	-3.9473	1	-8.5603
			167	CTC	0	0	0.488808	2.2482	0	0	0	0	0

			168	AAA	0	1	0.586729	2.41327	0	0.4144	0.41438	0.8044	0.898627
			169	GAC	0	0	0.584579	2.41542	0	0	0	0	0
			170	TTC	1	0	0.649079	2.20792	1.5406	0	-1.5406	1	-3.34109
			171	GAG	0	0	0.584579	2	0	0	0	0	0
			172	TAC	1	1	0.997926	2.00044	1.0021	0.4999	-0.5022	0.8892	-1.08906
			173	ACG	0	0	1	2	0	0	0	0	0
			174	GCA	1	0	1.02675	1.91391	0.9739	0	-0.9739	1	-2.11214
			175	CGC	0	0	0.592643	2.40736	0	0	0	0	0
			176	CAT	0	0	1.58458	1.41542	0	0	0	0	0
			177	CTA	1	0	0.591824	2.40818	1.6897	0	-1.6897	1	-3.66432
			178	TTT	1	0	1	2	1	0	-1	1	-2.16863
			179	CCT	0	0	0.584579	2.41542	0	0	0	0	0
			180	GAC	0	1	0.998413	1.99977	0	0.5001	0.50006	0.667	1.08444
			181	CCA	0	0	1	2	0	0	0	0	0
			182	GTT	3	1	1	2	3	0.5	-2.5	0.9877	-5.42158
			183	GCC	0	0	1	2	0	0	0	0	0
			184	ACT	0	0	0.584579	2	0	0	0	0	0
			185	TAC	0	1	1	2	0	0.5	0.5	0.6667	1.08432
			186	ACA	2	1	1	2	2	0.5	-1.5	0.963	-3.25295
			187	CCT	0	0	0.667188	2.18982	0	0	0	0	0
			188	GAG	1	0	0.591824	2.40818	1.6897	0	-1.6897	1	-3.66432
			189	TTT	2	0	1	2	2	0	-2	1	-4.33726
			190	CCA	0	1	0.984575	2.01543	0	0.4962	0.49617	0.6718	1.07602
			191	ACC	0	2	0.489643	2.36855	0	0.8444	0.8444	0.6867	1.83119
			192	GAA	2	0	1	1.62835	2	0	-2	1	-4.33726

			193	TCA	0	0	1	2	0	0	0	0	0
			194	CTC	6	1	0.756755	2.24325	7.9286	0.4458	-7.4828	0.9999	-16.2274
			195	ATT	5	0	0.585707	2.14692	8.5367	0	-8.5367	1	-18.5129
			196	TGC	1	0	0.592593	2.40741	1.6875	0	-1.6875	1	-3.65956
			197	CAT	3	0	0.660961	2.19604	4.5389	0	-4.5389	1	-9.84308
			198	GAG	0	0	1	2	0	0	0	0	0
			199	ACC	0	0	0.883956	2.11604	0	0	0	0	0
			200	ATT	0	0	0.488808	2.3682	0	0	0	0	0
			201	GAA	0	0	0.584579	2.41542	0	0	0	0	0
			202	AGC	0	0	0	1.66562	0	0	0	0	0
			203	TGG	0	0	0.488808	2.2482	0	0	0	0	0
			204	AAA	0	0	0.592643	2.40736	0	0	0	0	0
			205	AAT	0	0	0.488808	2.3682	0	0	0	0	0
			206	GAA	1	0	0.591824	2.40818	1.6897	0	-1.6897	1	-3.66432
			207	CAT	2	0	1.26781	1.73219	1.5775	0	-1.5775	1	-3.42106
			208	CTA	0	0	0.584579	2	0	0	0	0	0
			209	TAC	5	0	1	1.99945	5	0	-5	1	-10.8432
			210	TCT	0	0	0.667188	2.18982	0	0	0	0	0
			211	GAG	0	0	1	1.58458	0	0	0	0	0
			212	TCA	4	0	1	2	4	0	-4	1	-8.67452
			213	CCG	1	0	1	1.98548	1	0	-1	1	-2.16863
			214	GGG	1	0	0.950722	2.04928	1.0518	0	-1.0518	1	-2.28104
			215	AGG	0	0	0.592643	2.40736	0	0	0	0	0
			216	CAT	4	0	0.641749	2.09525	6.233	0	-6.233	1	-13.517
			217	AAG	0	0	1	2	0	0	0	0	0

			218	TCC	0.5	1.5	0.843537	2.12889	0.5927	0.7046	0.11185	0.7162	0.242559
			219	ATC	0	0	0.584579	2	0	0	0	0	0
			220	TAC	0	2	0.848528	2.15147	0	0.9296	0.9296	0.5143	2.01595
			221	ATC	0	0	0.584579	2.15194	0	0	0	0	0
			222	TGC	2	0	1	1.98439	2	0	-2	1	-4.33726
			223	GGG	0	0	1	2	0	0	0	0	0
			224	CCA	4	0	1	2	4	0	-4	1	-8.67452
			225	ACC	0	0	0.737004	2	0	0	0	0	0
			226	AGA	1	0	1	2	1	0	-1	1	-2.16863
			227	ACA	1	1	0.99864	1.85819	1.0014	0.5382	-0.4632	0.8778	-1.00452
			228	GGA	0	0	0.667188	2.06982	0	0	0	0	0
			229	AAG	0	0	1	2	0	0	0	0	0
			230	ACT	0	0	1	2	0	0	0	0	0
			231	TCC	0	0	0	1.66562	0	0	0	0	0
			232	TGG	0	0	1	2	0	0	0	0	0
			233	GCA	1	0	0.917548	2.08245	1.0899	0	-1.0899	1	-2.36351
			234	AGG	1	0	1	2	1	0	-1	1	-2.16863
			235	TCT	0	0	1.58458	1.41542	0	0	0	0	0
			236	CTA	0	1	1	2	0	0.5	0.5	0.6667	1.08432
			237	GGG	1	0	1	2	1	0	-1	1	-2.16863
			238	ACA	7	0	0.590947	2.40905	11.845	0	-11.845	1	-25.6883
			239	CAT	2	0	0.591799	2.4082	3.3795	0	-3.3795	1	-7.32894
			240	AAT	1	0	0.591824	2	1.6897	0	-1.6897	1	-3.66432
			241	TAT	1	0	0.591824	2	1.6897	0	-1.6897	1	-3.66432
			242	TAT	0.5	1.5	0.585771	2.41396	0.8536	0.6214	-0.2322	0.8047	-0.503534

			243	AAC	1	1	0.593932	2.40607	1.6837	0.4156	-1.2681	0.9608	-2.74999
			244	AGT	1	0	1.58458	1.41542	0.6311	0	-0.6311	1	-1.36858
			245	CTA	0	0	1	2	0	0	0	0	0
			246	GTT	8	1	0.591332	2.40763	13.529	0.4153	-13.113	1	-28.4382
			247	GAC	0	1	0.586729	2.41327	0	0.4144	0.41438	0.8044	0.898627
			248	TTC	2	0	1	2	2	0	-2	1	-4.33726
			249	ACA	0	0	1	2	0	0	0	0	0
			250	ACA	2	0	0.592601	2	3.375	0	-3.375	1	-7.31902
			251	TAT	0	0	0.584579	2.41542	0	0	0	0	0
			252	GAC	1	0	1	2	1	0	-1	1	-2.16863
			253	GTC	0	1	0.584579	2.41542	0	0.414	0.41401	0.8051	0.897827
			254	AAC	0	0	1	2	0	0	0	0	0
			255	GCC	6	1	0.662698	2.07567	9.0539	0.4818	-8.5721	1	-18.5898
			256	AAG	2	1	0.590708	2.00296	3.3858	0.4993	-2.8865	0.9882	-6.25977
			257	TAT	1	0	0.591824	2.40818	1.6897	0	-1.6897	1	-3.66432
			258	AAT	1	1	0.852082	2.14792	1.1736	0.4656	-0.708	0.9193	-1.53545
			259	ATC	0	0	0.848056	2.15194	0	0	0	0	0
			260	ATC	1	0	0.58473	2.41527	1.7102	0	-1.7102	1	-3.70877
			261	GAC	0	0	0.584579	2.41542	0	0	0	0	0
			262	GAC	1	0	0.880311	2.11969	1.136	0	-1.136	1	-2.46348
			263	ATT	0	0	1	2	0	0	0	0	0
			264	CCA	3	0	0.584621	2.41538	5.1315	0	-5.1315	1	-11.1284
			265	TTC	0	1	0.667444	2.06983	0	0.4831	0.48313	0.7562	1.04773
			266	AAG	0	0	0.584579	2.41542	0	0	0	0	0
			267	TTC	0	0	1	2	0	0	0	0	0

			268	ACA	2	1	1	2	2	0.5	-1.5	0.963	-3.25295
			269	CCC	0	0	0.584579	2.41542	0	0	0	0	0
			270	AAC	0	0	0	1.66562	0	0	0	0	0
			271	TGG	0	0	0.667188	2.06982	0	0	0	0	0
			272	AAG	0	0	0.584579	2.15194	0	0	0	0	0
			273	TGC	0	0	0.584579	2.41542	0	0	0	0	0
			274	TTC	0	0	1	2	0	0	0	0	0
			275	GTC	5	0	1	1.99165	5	0	-5	1	-10.8432
			276	GGG	0	0	1	2	0	0	0	0	0
			277	GCT	0	0	0.667188	1.74823	0	0	0	0	0
			278	CAG	1	2	0.975222	1.98569	1.0254	1.0072	-0.0182	0.746	-0.039469
			279	CGT	0	2	0.58604	2.41292	0	0.8289	0.82887	0.6474	1.79752
280	280	280	280	GAC	0	0	0.584579	2.41542	0	0	0	0	0
			281	TTC	1	0	1	2	1	0	-1	1	-2.16863
			282	ACG	0	0	1	2	0	0	0	0	0
			283	GTC	2	0	0.592339	2.40766	3.3765	0	-3.3765	1	-7.32227
			284	AAT	1	0	1	2	1	0	-1	1	-2.16863
			285	CCA	1	0	0.506917	2.23009	1.9727	0	-1.9727	1	-4.27808
			286	AAA	2	0	0.588439	2	3.3988	0	-3.3988	1	-7.37079
			287	TAC	1	0	1	2	1	0	-1	1	-2.16863
			288	GGT	0	1	0.668986	2.06992	0	0.4831	0.48311	0.7557	1.04769
			289	AAG	2	0	1.23646	1.23892	1.6175	0	-1.6175	1	-3.50781
			290	CGA	1	0	0.489908	2.2471	2.0412	0	-2.0412	1	-4.42661
			291	AAA	7	3	0.884555	2.11544	7.9136	1.4181	-6.4954	0.9986	-14.0862
			292	GTG	0	0	0.511192	2.48881	0	0	0	0	0

			293	ATA	0	0	1.26348	1.73652	0	0	0	0	0
			294	CGG	1	0	1	2	1	0	-1	1	-2.16863
			295	GGT	7	0	1	1.97508	7	0	-7	1	-15.1804
			296	GGC	0	0	0.511192	2.48881	0	0	0	0	0
			297	ATA	0	0	1	2	0	0	0	0	0
			298	CCT	0	0	0.584579	2.15194	0	0	0	0	0
			299	TGC	0	0	0.848056	2.15194	0	0	0	0	0
			300	ATC	0	0	0.883956	2.11604	0	0	0	0	0
			301	ATT	0	0	1.08145	1.51119	0	0	0	0	0
			302	TTA	1	1	1	2	1	0.5	-0.5	0.8889	-1.08432
			303	GTT	1	0	0.591824	2.40818	1.6897	0	-1.6897	1	-3.66432
			304	AAT	0	0	1	2	0	0	0	0	0
			305	CCA	0	0	0.584579	2.41542	0	0	0	0	0
			306	GAC	1	0	0.488992	2.36801	2.045	0	-2.045	1	-4.4349
			307	GAA	0	0	0.592643	2.40736	0	0	0	0	0
			308	GAT	0	1	0.00623261	1.66771	0	0.5996	0.59963	0.9963	1.30037
			309	TGG	0	0	1	2	0	0	0	0	0
			310	CTC	6	0	0.643728	2.09328	9.3207	0	-9.3207	1	-20.2132
			311	AAG	0	0	0.592643	2.40736	0	0	0	0	0
			312	GAT	0	0	0	3	0	0	0	0	0
			313	ATG	1	0	1	2	1	0	-1	1	-2.16863
			314	ACT	1	0	1	2	1	0	-1	1	-2.16863
			315	CCC	0	1	0.499349	2.37217	0	0.4216	0.42156	0.8261	0.914196
			316	GAA	0	0	0.667188	1.74823	0	0	0	0	0
			317	CAG	0	0	1	1.73652	0	0	0	0	0

			318	TCG	0	0	0.592643	2.40736	0	0	0	0	0
			319	GAT	0	0	0.584579	2	0	0	0	0	0
			320	TAC	0	2	0.0029282	2.99707	0	0.6673	0.66732	0.998	1.44717
			321	ATG	0	0	0.584579	2	0	0	0	0	0
			322	TAC	1	0	1	2	1	0	-1	1	-2.16863
			323	TCT	0	0	0.584579	2.41542	0	0	0	0	0
			324	AAC	0	1	1	2	0	0.5	0.5	0.6667	1.08432
			325	GCT	0	1	0.988219	2.01178	0	0.4971	0.49707	0.6706	1.07797
			326	GTT	2	0	1	2	2	0	-2	1	-4.33726
			327	GTT	1	0	0.585398	2.4146	1.7082	0	-1.7082	1	-3.70454
			328	CAC	1	0	0.585398	2	1.7082	0	-1.7082	1	-3.70454
			329	TAC	0.5	1.5	0.00363278	2.99637	137.64	0.5006	-137.14	0.9988	-297.396
			330	ATG	0	0	0.592643	2	0	0	0	0	0
			331	TAT	0	1	0.489601	2.36859	0	0.4222	0.42219	0.8287	0.91558
			332	GAA	1	0	1	2	1	0	-1	1	-2.16863
			333	GGC	1	1	0.651502	2.20654	1.5349	0.4532	-1.0817	0.948	-2.34585
			334	GAG	6.6667	6.33333	0.996181	1.89067	6.6922	3.3498	-3.3424	0.9312	-7.24853
335		335	335	ACG	1	0	0.585398	2.4146	1.7082	0	-1.7082	1	-3.70454
			336	TTC	1	1	0.823069	2.17693	1.215	0.4594	-0.7556	0.9247	-1.63862
			337	ATC	1	2	0.594785	2.39129	1.6813	0.8364	-0.8449	0.8968	-1.83231
			338	AAC	1	1	0.585398	2.04163	1.7082	0.4898	-1.2184	0.9503	-2.64234
			339	TAC	4.5	1.5	0.981223	1.77697	4.5861	0.8441	-3.742	0.987	-8.11497
			340	TCG	0	0	0.584579	2.41542	0	0	0	0	0
			341	TTC	1	0	1	2	1	0	-1	1	-2.16863
			342	GCC	2	3	0.955676	2.03348	2.0928	1.4753	-0.6175	0.8099	-1.33903

343	343	343	343	TCC	1	2	0.998967	1.99589	1.001	1.0021	0.00102	0.7404	0.0022224
			344	GGC	3.5	4.5	0.50515	2.35574	6.9286	1.9102	-5.0184	0.9784	-10.8831
345		345	345	GAA	1	4	0.600695	2.39931	1.6647	1.6672	0.00241	0.7368	0.0052266
346		346	346	GAT	1	2	1	2	1	1	0	0.7407	0
			347	GTC	1	1	1	1.99651	1	0.5009	-0.4991	0.8886	-1.08242
			348	ACT	1	4	0.995489	2.00352	1.0045	1.9965	0.99195	0.4637	2.15117
			349	GCT	0	0	1	1.73652	0	0	0	0	0
			350	TCG	0	0	0.667188	1.74823	0	0	0	0	0

D.

Coevolutionary sites of intra-protein	Codon#	Triplet	Syn (s)	Nonsyn (n)	Syn sites (S)	Nonsyn sites (N)	d_S	d_N	d_N - d_S	P-value	Normalized d_N - d_S
	1	ATG	0	0	0	3	0	0	0	0	0
	2	GCC	1	4	1	2	1	2	1	0.4609	2.58058
	3	TCT	0	1	0.996298	2.0037	0	0.4991	0.49908	0.6679	1.28791
	4	TCA	0	0	1	1.54651	0	0	0	0	0
	5	TCT	0	1	0.996298	2.0037	0	0.4991	0.49908	0.6679	1.28791
	6	ACA	1	3	1	1.99851	1	1.5011	0.50112	0.5923	1.29317
	7	CCC	0	1	0.99702	2.00298	0	0.4993	0.49926	0.6677	1.28837
	8	AGG	0	1	0.904486	2.09551	0	0.4772	0.47721	0.6985	1.23148
	9	TTC	0	0	0.546508	2.45349	0	0	0	0	0
	10	CGA	8	0	1.07322	1.78689	7.4542	0	-7.4542	1	-19.2362
	11	GTC	2	0	1	2	2	0	-2	1	-5.16116
	12	TAT	0	0	0.549382	2	0	0	0	0	0
	13	TCC	2	0	1	1.89347	2	0	-2	1	-5.16116
	14	AAG	1	0	0.58457	2.10686	1.7107	0	-1.7107	1	-4.41449
	15	TAC	0	1	0.546508	2.00148	0	0.4996	0.49963	0.7855	1.28933
	16	CTC	0	1	1	2	0	0.5	0.5	0.6667	1.29029
	17	TTT	1	0	0.54871	2.45129	1.8225	0	-1.8225	1	-4.703
	18	CTA	0	1	1.54561	1.45439	0	0.6876	0.68758	0.4848	1.77434
	19	ACA	0	0	1	2	0	0	0	0	0

	20	TAT	0	0	0.549382	2	0	0	0	0	0
	21	CCT	0	0	1	2	0	0	0	0	0
	22	CAA	0	1	0.420205	2.03395	0	0.4917	0.49165	0.8288	1.26875
	23	TGT	1	0	0.54871	2.11853	1.8225	0	-1.8225	1	-4.703
	24	ACC	0	2	0.959346	2.04065	0	0.9801	0.98008	0.4627	2.52917
	25	CTT	0	0	1	2	0	0	0	0	0
	26	GAG	1	2	0.58457	2.27496	1.7107	0.8791	-0.8315	0.8917	-2.14581
	27	CCA	1	1	1.00811	1.99189	0.992	0.502	-0.4899	0.8871	-1.26428
	28	CAG	0	2	0.633004	1.82681	0	1.0948	1.0948	0.5515	2.82522
	29	TAC	1	3	0.553251	2.1083	1.8075	1.423	-0.3846	0.807	-0.992369
	30	GCC	0	3	1.17989	1.47873	0	2.0288	2.02877	0.1721	5.23539
	31	TTG	0	2	0.557117	2.43028	0	0.823	0.82295	0.6618	2.12369
	32	GAT	1	1	1	1.58909	1	0.6293	-0.3707	0.8508	-0.956639
	33	TCA	1	2	1.00591	1.99409	0.9941	1.003	0.00883	0.7381	0.0227929
	34	CTT	1	1	0.999367	1.99875	1.0006	0.5003	-0.5003	0.8889	-1.29112
	35	CGC	0.5	1.5	0.993493	2.00311	0.5033	0.7488	0.24556	0.6685	0.633692
	36	ACT	0	1	1	2	0	0.5	0.5	0.6667	1.29029
	37	CTC	1.5	2.5	1.18143	1.4773	1.2697	1.6923	0.42263	0.5831	1.09062
	38	TTG	0	3	0.652574	2.34743	0	1.278	1.278	0.4791	3.29797
	39	AAC	0	3	0.429788	2.26906	0	1.3221	1.32213	0.5943	3.41187
	40	AAA	0	1	0.549382	2.00171	0	0.4996	0.49957	0.7846	1.28919
	41	TAT	0	0	0.63475	2.22612	0	0	0	0	0
	42	GAG	2	0	1	2	2	0	-2	1	-5.16116
	43	CCC	2	1	1.00179	1.99821	1.9964	0.5004	-1.496	0.9628	-3.86049
	44	CTC	0	0	0.546508	2	0	0	0	0	0

	45	TAC	0	0	0.832532	2.16747	0	0	0	0	0
	46	ATC	1	0	1	2	1	0	-1	1	-2.58058
	47	GCT	1	3	1	2	1	1.5	0.5	0.5926	1.29029
	48	GCT	0	0	1	2	0	0	0	0	0
	49	GTT	0	0	0.691427	2	0	0	0	0	0
	50	AGA	0	0	0.63475	2.22612	0	0	0	0	0
	51	GAG	1	0	1	2	1	0	-1	1	-2.58058
	52	CTC	0	0	0.546508	2.45349	0	0	0	0	0
	53	CAC	0	0	0.420205	2.44066	0	0	0	0	0
	54	GAA	0	1	0.549382	2.45062	0	0.4081	0.40806	0.8169	1.05303
	55	GAT	0	0	1	1.86087	0	0	0	0	0
	56	GGA	0	0	1	1.54651	0	0	0	0	0
	57	TCA	0	0	1	2	0	0	0	0	0
	58	CCA	0	0	0.549382	2.45062	0	0	0	0	0
	59	CAT	3	0	1.53698	1.45734	1.9519	0	-1.9519	1	-5.03699
	60	CTG	0	0	0.546508	2.45349	0	0	0	0	0
	61	CAC	1	1	0.99966	2.00034	1.0003	0.4999	-0.5004	0.889	-1.29139
	62	GTT	0	0	1	2	0	0	0	0	0
	63	CTC	0	0	1	2	0	0	0	0	0
	64	GTG	2	0	0.614255	1.83924	3.256	0	-3.256	1	-8.4023
	65	CAG	0	0	0.546508	2.45349	0	0	0	0	0
	66	AAC	0	1	0.637426	2.05705	0	0.4861	0.48613	0.7634	1.25451
67	67	AAG	0	0	1	2	0	0	0	0	0
	68	CTT	0	0	1	2	0	0	0	0	0
	69	CGT	0	0	1	2	0	0	0	0	0

	70	GCT	4	0	1	1.90649	4	0	-4	1	-10.3223
	71	TCC	0	0	0.832532	2.16747	0	0	0	0	0
	72	ATC	0	0	1	2	0	0	0	0	0
	73	ACC	0	0	0.549382	2.45062	0	0	0	0	0
	74	AAT	1	0	1	2	1	0	-1	1	-2.58058
	75	CCC	0	2	0.549382	2.45062	0	0.8161	0.81612	0.6673	2.10606
76	76	AAT	3	0	1	2	3	0	-3	1	-7.74174
	77	GCC	2	1	0.983898	1.76194	2.0327	0.5676	-1.4652	0.954	-3.781
	78	TTA	0	1	0.546508	2.44902	0	0.4083	0.40833	0.8176	1.05372
79	79	AAC	3	0	1.00089	1.99911	2.9973	0	-2.9973	1	-7.73482
	80	CTC	0	0	1	2	0	0	0	0	0
	81	CGT	0	0	0	3	0	0	0	0	0
	82	ATG	1	0	0.54871	2.45129	1.8225	0	-1.8225	1	-4.703
	83	GAT	0	0	1	2	0	0	0	0	0
	84	ACA	1	0	1	2	1	0	-1	1	-2.58058
	85	TCT	0	0	1	2	0	0	0	0	0
	86	CCA	3	1	0.550018	2.44702	5.4544	0.4087	-5.0457	0.9989	-13.0209
	87	TTC	1	2	0.959168	2.02722	1.0426	0.9866	-0.056	0.7568	-0.144509
	88	TCC	2	0	0.654367	2.34563	3.0564	0	-3.0564	1	-7.88726
	89	ATA	1	0	0.546522	2.45348	1.8298	0	-1.8298	1	-4.72182
	90	TTC	1	0	0.54871	2.45129	1.8225	0	-1.8225	1	-4.703
	91	CAT	1	0	1	2	1	0	-1	1	-2.58058
	92	CCT	0	1	0.549382	2.44617	0	0.4088	0.4088	0.8166	1.05495
93	93	AAT	1	2	0.636909	2.36106	1.5701	0.8471	-0.723	0.8838	-1.86578
94	94	ATA	1	0	0.470384	1.98311	2.1259	0	-2.1259	1	-5.48611

	95	CAA	1	0	1	2	1	0	-1	1	-2.58058
	96	GCT	0	0	1	2	0	0	0	0	0
	97	GCC	0	0	0.420205	2.27122	0	0	0	0	0
	98	AAA	3	1	0.548708	2.45129	5.4674	0.4079	-5.0594	0.9989	-13.0563
	99	GAC	0	1	0.549488	2.16637	0	0.4616	0.4616	0.7977	1.1912
	100	TGC	1	1	0.546607	2.45339	1.8295	0.4076	-1.4219	0.9668	-3.66925
	101	AAC	1	1	0.426629	2.03226	2.344	0.4921	-1.8519	0.9699	-4.77896
102	102	CAA	1	0	1	2	1	0	-1	1	-2.58058
	103	GTT	0	0	1	2	0	0	0	0	0
	104	CGT	0	0	0.549382	2.45062	0	0	0	0	0
	105	GAT	2	0	0.546574	2	3.6592	0	-3.6592	1	-9.44275
	106	TAC	0	1	0.833355	2.16665	0	0.4615	0.46154	0.7222	1.19105
	107	ATC	0	1	1	2	0	0.5	0.5	0.6667	1.29029
	108	ACG	3	1	0.606191	2.08727	4.9489	0.4791	-4.4698	0.9974	-11.5348
109	109	AAG	1	0	0.634047	2.22682	1.5772	0	-1.5772	1	-4.07001
	110	GAG	0	0	1	2	0	0	0	0	0
	111	GTT	0	0	0.546508	2.45349	0	0	0	0	0
	112	GAC	0	0	1	2	0	0	0	0	0
	113	TCC	0	0	0.549382	2.45062	0	0	0	0	0
	114	GAT	1	5	1.0009	1.9991	0.9991	2.5011	1.50201	0.3506	3.87607
	115	GTA	0	0	0.546508	2.45349	0	0	0	0	0
	116	AAC	1	0	1	2	1	0	-1	1	-2.58058
	117	ACA	0	0	1	2	0	0	0	0	0
	118	GCT	0	0	0.63475	2.22612	0	0	0	0	0
	119	GAG	0	0	0	1.7305	0	0	0	0	0

	120	TGG	0	0	1	1.86087	0	0	0	0	0
	121	GGA	0	0	1	2	0	0	0	0	0
	122	ACA	0	0	0.546508	2.45349	0	0	0	0	0
	123	TTC	1.5	1.5	0.944411	2.05559	1.5883	0.7297	-0.8586	0.8669	-2.21562
	124	GTG	4	1	1	2	4	0.5	-3.5	0.9959	-9.03203
	125	GCT	0	1	1	2	0	0.5	0.5	0.6667	1.29029
	126	GTT	1	1	1	1.6013	1	0.6245	-0.3755	0.8522	-0.969025
	127	TCA	1	0	1	2	1	0	-1	1	-2.58058
	128	ACT	1	1	1	2	1	0.5	-0.5	0.8889	-1.29029
	129	CCA	0	0	1	2	0	0	0	0	0
	130	GGT	0	0	1	2	0	0	0	0	0
	131	CGT	1	0	0.420908	2.27052	2.3758	0	-2.3758	1	-6.13099
	132	AAA	0	1	0.547993	2.45201	0	0.4078	0.40783	0.8173	1.05244
	133	GAC	0	0	1	2	0	0	0	0	0
	134	CGT	5	0	0.549027	2.45097	9.107	0	-9.107	1	-23.5014
	135	GAT	2	0	1	2	2	0	-2	1	-5.16116
	136	GCG	0	0	0.549382	2.45062	0	0	0	0	0
	137	GAT	0	0	0	3	0	0	0	0	0
	138	ATG	0	0	0.420205	2.27122	0	0	0	0	0
	139	AAA	1	0	0.622454	1.83104	1.6065	0	-1.6065	1	-4.14581
	140	CAG	0	0	0.832532	2.16747	0	0	0	0	0
	141	ATC	1	0	0.890627	2.10937	1.1228	0	-1.1228	1	-2.89749
	142	ATT	1	0	0.439646	2.42122	2.2746	0	-2.2746	1	-5.86968
	143	GAA	5	0	1	1.95668	5	0	-5	1	-12.9029
	144	TCT	0	2	0.559739	2.43628	0	0.8209	0.82092	0.6613	2.11846

	145	AGT	0	0	1	2	0	0	0	0	0
	146	TCC	1	0	1	2	1	0	-1	1	-2.58058
	147	TCT	3	0	1.00094	1.99906	2.9972	0	-2.9972	1	-7.73449
	148	CGC	0	1	0.63475	2.22529	0	0.4494	0.44938	0.7781	1.15966
	149	GAG	0	0	0.420205	2.44066	0	0	0	0	0
	150	GAA	2	0	0.546773	2.45323	3.6578	0	-3.6578	1	-9.43931
	151	TTC	0	0	1	2	0	0	0	0	0
	152	CTC	1	0	0.546527	2.45347	1.8297	0	-1.8297	1	-4.72178
	153	AGC	0	0	0	3	0	0	0	0	0
	154	ATG	1	0	1	2	1	0	-1	1	-2.58058
	155	GTT	0	0	0.546508	2.16747	0	0	0	0	0
	156	TGC	0	2	0.549382	2.45062	0	0.8161	0.81612	0.6673	2.10606
	157	AAT	1	0	1.00047	1.99864	0.9995	0	-0.9995	1	-2.57937
	158	CGT	1	1	0.552329	2.44767	1.8105	0.4086	-1.402	0.9661	-3.61788
	159	TTT	1	0	1	2	1	0	-1	1	-2.58058
	160	CCG	0	0	0.549382	2.45062	0	0	0	0	0
	161	TTT	0	0	0.420205	2.44066	0	0	0	0	0
	162	GAA	0	0	0	1.7305	0	0	0	0	0
	163	TGG	3	0	1	1.9328	3	0	-3	1	-7.74174
	164	TCT	3	0	0.773223	2.22678	3.8799	0	-3.8799	1	-10.0123
	165	ATC	0	0	1	2	0	0	0	0	0
	166	CGT	4	0	1.01799	1.98201	3.9293	0	-3.9293	1	-10.1399
	167	CTC	0	0	0.420205	2.27122	0	0	0	0	0
	168	AAA	0	1	0.54725	2.45275	0	0.4077	0.40771	0.8176	1.05212
	169	GAC	0	0	0.546508	2.45349	0	0	0	0	0

	170	TTC	1	0	0.615309	2.24556	1.6252	0	-1.6252	1	-4.19396
	171	GAG	0	0	0.546508	2	0	0	0	0	0
	172	TAC	1	1	0.9976	2.00037	1.0024	0.4999	-0.5025	0.8893	-1.29674
	173	ACG	0	0	1	2	0	0	0	0	0
	174	GCA	1	0	1.02592	1.92456	0.9747	0	-0.9747	1	-2.51539
	175	CGC	0	0	0.549382	2.45062	0	0	0	0	0
	176	CAT	0	0	1.54651	1.45349	0	0	0	0	0
	177	CTA	1	0	0.549121	2.45088	1.8211	0	-1.8211	1	-4.69947
	178	TTT	1	0	1	2	1	0	-1	1	-2.58058
	179	CCT	0	0	0.546508	2.45349	0	0	0	0	0
	180	GAC	0	1	0.998101	2.00011	0	0.5	0.49997	0.6671	1.29022
	181	CCA	0	0	1	2	0	0	0	0	0
	182	GTT	1	1	1	2	1	0.5	-0.5	0.8889	-1.29029
	183	GCC	0	0	1	2	0	0	0	0	0
	184	ACT	0	0	0.546508	2	0	0	0	0	0
	185	TAC	0	1	1	2	0	0.5	0.5	0.6667	1.29029
	186	ACA	2	1	1	2	2	0.5	-1.5	0.963	-3.87087
	187	CCT	0	0	0.63475	2.22612	0	0	0	0	0
	188	GAG	1	0	0.549121	2.45088	1.8211	0	-1.8211	1	-4.69947
	189	TTT	2	0	1	2	2	0	-2	1	-5.16116
	190	CCA	0	1	0.984825	2.01517	0	0.4962	0.49624	0.6717	1.28057
	191	ACC	0	2	0.421254	2.44076	0	0.8194	0.81942	0.7273	2.11458
	192	GAA	2	0	1	1.59058	2	0	-2	1	-5.16116
	193	TCA	0	0	1	2	0	0	0	0	0
	194	CTC	4	1	0.781398	2.2186	5.119	0.4507	-4.6683	0.9988	-12.0469

	195	ATT	4	0	0.546905	2.15864	7.3139	0	-7.3139	1	-18.8741
	196	TGC	1	0	0.549372	2.45063	1.8203	0	-1.8203	1	-4.69732
	197	CAT	3	0	0.624546	2.23632	4.8035	0	-4.8035	1	-12.3958
	198	GAG	0	0	1	2	0	0	0	0	0
	199	ACC	0	0	0.896415	2.10358	0	0	0	0	0
	200	ATT	0	0	0.420205	2.44066	0	0	0	0	0
	201	GAA	0	0	0.546508	2.45349	0	0	0	0	0
	202	AGC	0	0	0	1.7305	0	0	0	0	0
	203	TGG	0	0	0.420205	2.27122	0	0	0	0	0
	204	AAA	0	0	0.549382	2.45062	0	0	0	0	0
	205	AAT	0	0	0.420205	2.44066	0	0	0	0	0
	206	GAA	1	0	0.549121	2.45088	1.8211	0	-1.8211	1	-4.69947
	207	CAT	2	0	1.25449	1.74551	1.5943	0	-1.5943	1	-4.11416
	208	CTA	0	0	0.546508	2	0	0	0	0	0
	209	TAC	5	0	1	1.99812	5	0	-5	1	-12.9029
	210	TCT	0	1	1.00359	1.99641	0	0.5009	0.5009	0.6655	1.29261
	211	GTA	0	1	0.546508	2.45349	0	0.4076	0.40758	0.8178	1.0518
	212	AGC	0	0	1	2	0	0	0	0	0
	213	CTC	0	0	0.420205	2.44066	0	0	0	0	0
	214	GAA	4	0	1	1.9232	4	0	-4	1	-10.3223
	215	TCC	1	1	0.549121	2.00074	1.8211	0.4998	-1.3213	0.9536	-3.40966
	216	TAT	1	0	0.832636	2.16736	1.201	0	-1.201	1	-3.09929
	217	ATC	0	1	0.99983	2.00017	0	0.5	0.49996	0.6667	1.29018
	218	CTT	0	0	0.549382	2.10358	0	0	0	0	0
	219	TGT	0	0	1	2	0	0	0	0	0

	220	ACT	1	0	1	1.99702	1	0	-1	1	-2.58058
	221	TCC	0	0	1	2	0	0	0	0	0
	222	ACT	0	0	1	2	0	0	0	0	0
	223	CCT	0	0	1	2	0	0	0	0	0
	224	GCG	0	0	0.549382	2.45062	0	0	0	0	0
	225	GAT	1	2	0.420908	2.05615	2.3758	0.9727	-1.4031	0.9232	-3.62088
	226	CAA	1	0	1	2	1	0	-1	1	-2.58058
	227	GCG	0	2	0.48925	2.02607	0	0.9871	0.98713	0.6488	2.54738
	228	CAA	0	0	1	2	0	0	0	0	0
	229	TCT	0	0	0.546508	2.45349	0	0	0	0	0
	230	GAC	0	0	0.969587	1.57979	0	0	0	0	0
	231	TTA	0	0	0.63475	2.22612	0	0	0	0	0
	232	GAG	0	1	0.00361364	1.73295	0	0.577	0.57705	0.9979	1.48912
233	233	TGG	0	1	0.00103852	2.99846	0	0.3335	0.33351	0.9997	0.860636
	234	ATG	0	0	0.546508	2.45349	0	0	0	0	0
	235	GAC	0	1	0.549382	2.45062	0	0.4081	0.40806	0.8169	1.05303
	236	GAT	0	1	0.549382	2.00222	0	0.4994	0.49945	0.7847	1.28886
	237	TAT	0	0	1	2	0	0	0	0	0
	238	TCC	0	1	0.903004	2.09284	0	0.4778	0.47782	0.6986	1.23305
	239	AGG	0	0	0.549382	2.45062	0	0	0	0	0
	240	AGT	0	0	0.546508	2.45349	0	0	0	0	0
	241	CAC	0	5	1.24528	1.72053	0	2.9061	2.90608	0.0657	7.49938
	242	CGG	0.5	1.5	0.955092	1.91838	0.5235	0.7819	0.2584	0.6676	0.666818
	243	GGA	0	0	1	2	0	0	0	0	0
	244	GGC	0	0	0.579795	2.42021	0	0	0	0	0

	245	ATA	0	3	0.549382	2.45062	0	1.2242	1.22418	0.5451	3.1591
	246	AGT	1	0	1	2	1	0	-1	1	-2.58058
	247	CCA	0	1	0.959168	2	0	0.5	0.5	0.6759	1.29029
	248	TCT	1	0	1	2	1	0	-1	1	-2.58058
	249	ACA	0	1	1	2	0	0.5	0.5	0.6667	1.29029
	250	TCT	0	0	1	2	0	0	0	0	0
	251	GCG	0	2	0.95297	2.04703	0	0.977	0.97703	0.4656	2.52129
	252	GGC	0	0	0.420205	2.03329	0	0	0	0	0
	253	CAA	0	4	0.944595	2.00051	0	1.9995	1.99949	0.2129	5.15985
	254	CCA	0	0	0.420205	2.44066	0	0	0	0	0
	255	GAA	1	1	0.637383	1.8179	1.5689	0.5501	-1.0188	0.9326	-2.62917
	256	CAG	0	1	0.423055	2.43781	0	0.4102	0.4102	0.8521	1.05856
	257	GAA	0	0	0.691427	2	0	0	0	0	0
	258	AGA	0	0	1	2	0	0	0	0	0
	259	CTT	0	0	1	2	0	0	0	0	0
	260	CCT	0	0	1	2	0	0	0	0	0
	261	GGG	0	1	0.420826	2.03535	0	0.4913	0.49132	0.8287	1.26788
	262	CAA	0	0	1	2	0	0	0	0	0
	263	GGT	0	1	1	2	0	0.5	0.5	0.6667	1.29029

E.

Coevolutionary sites of intra-protein	Codon#	Triplet	Syn (s)	Nonsyn (n)	Syn sites (S)	Nonsyn sites (N)	d_S	d_N	d_N - d_S	P-value	Normalized d_N - d_S
	1	ATG	0	0	0	3	0	0	0	0	0
	2	GTG	0	0	1	2	0	0	0	0	0
	3	ACC	0	0	1	2	0	0	0	0	0
	4	AAC	0	0	0.580584	2.41942	0	0	0	0	0
	5	AAG	0	0	0.555986	2.27732	0	0	0	0	0
	6	GAC	0	0	0.580584	2.41942	0	0	0	0	0
	7	TCC	0	0	1	2	0	0	0	0	0
	8	CGA	0	0	1.28935	1.13007	0	0	0	0	0
	9	GGT	0	0	1	2	0	0	0	0	0
	10	AAG	0	0	0.555986	2.27732	0	0	0	0	0
	11	GGA	0	0	1	1.70645	0	0	0	0	0
	12	AAG	0	0	0.555986	2.27732	0	0	0	0	0
	13	AGG	0	0	0.862346	2.13765	0	0	0	0	0
	14	AAG	0	0	0.555986	2.27732	0	0	0	0	0
	15	CTC	0	0	1	2	0	0	0	0	0
	16	GAG	0	0	0.555986	2.15047	0	0	0	0	0
	17	GAG	0	0	0.555986	2.15047	0	0	0	0	0
	18	GGC	0	0	1	2	0	0	0	0	0

	19	GAA	0	0	0.526942	2.17951	0	0	0	0	0
	20	TCG	0	1	1.00295	1.71046	0	0.5846	0.58464	0.6304	6.20618
	21	AGT	0	0	0.588602	2.4114	0	0	0	0	0
	22	GGA	0	0	1	1.70645	0	0	0	0	0
	23	AGG	0	0	0.862346	2.13765	0	0	0	0	0
	24	TGG	0	0	0	1.88803	0	0	0	0	0
	25	AAG	0	0	0.555986	2.27732	0	0	0	0	0
	26	GGG	0	0	1	2	0	0	0	0	0
	27	CAA	0	0	0.526942	1.89247	0	0	0	0	0
	28	GTG	0	0	1	2	0	0	0	0	0
	29	TAT	0	0	0.588602	2	0	0	0	0	0
	30	AAG	0	0	0.555986	2.27732	0	0	0	0	0
	31	CGG	1	0	1.26884	1.73116	0.7881	0	-0.7881	1	-8.3662
	32	CGT	0	0	1	2	0	0	0	0	0
33	33	AAG	0	3	0.596535	2.25847	0	1.3283	1.32834	0.495	14.1008
	34	CAG	3	4	0.593786	1.88538	5.0523	2.1216	-2.9307	0.9387	-31.111
	35	ACC	1	2	1	2	1	1	0	0.7407	0
	36	TAC	0	0	0.580584	2	0	0	0	0	0
	37	AAG	0	0	0.555986	2.27732	0	0	0	0	0
	38	GTA	0	0	1	2	0	0	0	0	0
	39	GTA	0	0	1	2	0	0	0	0	0
	40	CCT	2	0	1	2	2	0	-2	1	-21.2308
	41	GTG	0	0	1	2	0	0	0	0	0
	42	AGG	0	0	0.862346	2.13765	0	0	0	0	0
	43	CCA	0	0	1	2	0	0	0	0	0

	44	CCC	0	0	1	2	0	0	0	0	0
	45	GCC	0	0	1	2	0	0	0	0	0
	46	CTG	1	0	1.57663	1.42337	0.6343	0	-0.6343	1	-6.73299
	47	TGT	0	0	0.588602	2.25726	0	0	0	0	0
	48	GTG	0	0	1	2	0	0	0	0	0
	49	TAC	1	0	0.580638	2	1.7222	0	-1.7222	1	-18.2823
	50	CGG	0	0	1.28935	1.71065	0	0	0	0	0
	51	TAT	0	0	0.588602	2	0	0	0	0	0
	52	AAC	0	0	0.580584	2.41942	0	0	0	0	0
	53	TGG	0	0	0	1.88803	0	0	0	0	0
	54	CTC	0	0	1	2	0	0	0	0	0
	55	AAT	0	0	0.588602	2.4114	0	0	0	0	0
	56	TCG	1	0	1	1.59582	1	0	-1	1	-10.6154
	57	GAC	0	0	0.580584	2.41942	0	0	0	0	0
	58	AAG	0	0	0.555986	2.27732	0	0	0	0	0
	59	ACC	0	0	1	2	0	0	0	0	0
	60	AAT	0	0	0.588602	2.4114	0	0	0	0	0
	61	ATC	0	0	0.869933	2.13007	0	0	0	0	0
	62	GTG	0	0	1	2	0	0	0	0	0
	63	GTG	0	1	1	2	0	0.5	0.5	0.6667	5.3077
	64	GGT	0	0	1	2	0	0	0	0	0
	65	AAC	0	0	0.580584	2.41942	0	0	0	0	0
	66	ACC	0	0	1	2	0	0	0	0	0
	67	CCT	0	0	1	2	0	0	0	0	0
	68	CGG	2	0	1.28541	1.69072	1.5559	0	-1.5559	1	-16.5168

	69	ATT	0	0	0.742743	2.25726	0	0	0	0	0
	70	GAT	0	0	0.588602	2.4114	0	0	0	0	0
	71	CTG	0	0	1.58058	1.41942	0	0	0	0	0
	72	ATT	0	0	0.742743	2.25726	0	0	0	0	0
	73	ACC	0	0	1	2	0	0	0	0	0
	74	TGC	0	0	0.580584	2.13007	0	0	0	0	0
	75	TTT	0	1	0.606629	2.3793	0	0.4203	0.42029	0.7968	4.46157
	76	GCA	0	0	1	2	0	0	0	0	0
	77	GCG	0	0	1	2	0	0	0	0	0
	78	GGT	1	0	1	2	1	0	-1	1	-10.6154
	79	AAG	0	0	0.555986	2.27732	0	0	0	0	0
	80	GCC	0	0	1	2	0	0	0	0	0
	81	GAT	0	0	0.588602	2.4114	0	0	0	0	0
	82	AAT	0	0	0.588602	2.4114	0	0	0	0	0
	83	AAT	0	0	0.588602	2.4114	0	0	0	0	0
	84	CGG	0	0	1.28935	1.71065	0	0	0	0	0
	85	CAT	0	0	0.588602	2.4114	0	0	0	0	0
	86	ACA	0	0	1	2	0	0	0	0	0
	87	AAT	0	0	0.588602	2.4114	0	0	0	0	0
	88	CAG	0	0	0.555986	1.86343	0	0	0	0	0
	89	ACC	0	0	1	2	0	0	0	0	0
	90	ATC	0	2	0.872594	2.12741	0	0.9401	0.94011	0.5029	9.97967
	91	CTC	0	0	1	2	0	0	0	0	0
	92	TAC	0	0	0.580584	2	0	0	0	0	0
	93	AAG	0	0	0.555986	2.27732	0	0	0	0	0

	94	TTT	0	0	0.588602	2.4114	0	0	0	0	0
	95	AAC	0	0	0.580584	2.41942	0	0	0	0	0
	96	CTG	3	0	1.58058	1.41942	1.898	0	-1.898	1	-20.1484
	97	CAG	0	0	0.555986	1.86343	0	0	0	0	0
	98	GGG	0	0	1	2	0	0	0	0	0
	99	ACC	0	0	1	2	0	0	0	0	0
	100	TGC	1	0	0.587663	2.24236	1.7017	0	-1.7017	1	-18.0638
	101	TAC	0	0	0.580584	2	0	0	0	0	0
	102	GTG	0	0	1	2	0	0	0	0	0
	103	TCG	0	0	1	1.71065	0	0	0	0	0
	104	GAC	0	0	0.580584	2.41942	0	0	0	0	0
	105	TCC	2	0	1	2	2	0	-2	1	-21.2308
	106	TCC	1	0	1	1.70477	1	0	-1	1	-10.6154
	107	TGT	0	0	0.588602	2.25726	0	0	0	0	0
	108	ATG	0	0	0	3	0	0	0	0	0
	109	TTC	1	0	0.580858	2.41914	1.7216	0	-1.7216	1	-18.2754
	110	ATA	0	0	0.473058	2.52694	0	0	0	0	0
	111	ACC	0	0	1	2	0	0	0	0	0
	112	CCA	0	0	1	2	0	0	0	0	0
	113	ATC	0	0	0.869933	2.13007	0	0	0	0	0
	114	AGG	0	0	0.862346	2.13765	0	0	0	0	0
	115	GTT	0	0	1	2	0	0	0	0	0
	116	TAT	0	0	0.588602	2	0	0	0	0	0
	117	CAC	0	0	0.580584	2.41942	0	0	0	0	0
	118	TGG	0	0	0	1.88803	0	0	0	0	0

	119	TTG	1	0	1.15055	1.69742	0.8691	0	-0.8691	1	-9.22636
	120	GTC	0	0	1	2	0	0	0	0	0
	121	TAT	0	0	0.588602	2	0	0	0	0	0
	122	GAT	0	0	0.588602	2.4114	0	0	0	0	0
	123	GCA	1	0	1	2	1	0	-1	1	-10.6154
	124	GAG	0	0	0.555986	2.15047	0	0	0	0	0
	125	CCG	0	0	1	2	0	0	0	0	0
	126	AAA	1	0	0.552584	2.28072	1.8097	0	-1.8097	1	-19.2105
	127	CAG	0	0	0.555986	1.86343	0	0	0	0	0
	128	GCT	0	0	1	2	0	0	0	0	0
	129	ACG	0	0	1	2	0	0	0	0	0
	130	CCT	0	0	1	2	0	0	0	0	0
	131	GAC	0	0	0.580584	2.41942	0	0	0	0	0
	132	TCC	0	0	1	2	0	0	0	0	0
	133	ACT	0	0	1	2	0	0	0	0	0
	134	GAC	0	0	0.580584	2.41942	0	0	0	0	0
	135	ATA	2	0	0.822578	2.17742	2.4314	0	-2.4314	1	-25.8101
	136	TTT	0	0	0.588602	2.4114	0	0	0	0	0
137	137	ACG	0	1	1	2	0	0.5	0.5	0.6667	5.3077
	138	ATG	0	0	0	3	0	0	0	0	0
	139	CCG	2	0	1	2	2	0	-2	1	-21.2308
	140	TGG	0	0	0	1.88803	0	0	0	0	0
	141	AAT	0	0	0.588602	2.4114	0	0	0	0	0
	142	CTC	4	0	1.30208	1.69792	3.072	0	-3.072	1	-32.6106
	143	CTG	0	0	1.58058	1.41942	0	0	0	0	0

	144	CCG	0	0	1	2	0	0	0	0	0
	145	AGT	0	0	0.588602	2.4114	0	0	0	0	0
	146	TCA	0	0	1	1.58058	0	0	0	0	0
	147	TGG	0	0	0	1.88803	0	0	0	0	0
	148	ACT	0	0	1	2	0	0	0	0	0
	149	GTG	0	0	1	2	0	0	0	0	0
	150	CAG	1	0	0.554989	1.86443	1.8018	0	-1.8018	1	-19.1272
	151	AGG	0	0	0.862346	2.13765	0	0	0	0	0
	152	GCT	0	0	1	2	0	0	0	0	0
	153	TGG	0	1	0.0136271	1.88955	0	0.5292	0.52923	0.9928	5.61794
	154	TCG	0	0	1	1.71065	0	0	0	0	0
	155	CAT	0	0	0.588602	2.4114	0	0	0	0	0
	156	CGG	0	0	1.28935	1.71065	0	0	0	0	0
	157	TTC	0	0	0.580584	2.41942	0	0	0	0	0
	158	GTG	0	0	1	2	0	0	0	0	0
	159	GTG	0	0	1	2	0	0	0	0	0
	160	AAG	0	0	0.555986	2.27732	0	0	0	0	0
	161	AGG	0	0	0.862346	2.13765	0	0	0	0	0
	162	AAG	0	0	0.555986	2.27732	0	0	0	0	0
	163	TGG	0	0	0	1.88803	0	0	0	0	0
	164	TGC	0	0	0.580584	2.13007	0	0	0	0	0
	165	GTG	0	0	1	2	0	0	0	0	0
	166	AAC	0	0	0.580584	2.41942	0	0	0	0	0
	167	CTG	0	0	1.58058	1.41942	0	0	0	0	0
	168	GTC	0	0	1	2	0	0	0	0	0

	169	TCC	0	0	1	2	0	0	0	0	0
	170	GAT	0	0	0.588602	2.4114	0	0	0	0	0
	171	GGA	0	0	1	1.70645	0	0	0	0	0
	172	AGG	0	0	0.862346	2.13765	0	0	0	0	0
	173	AAG	0	0	0.555986	2.27732	0	0	0	0	0
	174	GTT	0	0	1	2	0	0	0	0	0
	175	GGA	1	0	1	1.71046	1	0	-1	1	-10.6154
	176	TCC	0	0	1	2	0	0	0	0	0
	177	AAG	0	1	0.555986	2.2745	0	0.4397	0.43966	0.8036	4.66714
	178	ACG	0	0	1	2	0	0	0	0	0
	179	GTG	0	0	1	2	0	0	0	0	0
	180	GAC	0	0	0.580584	2.41942	0	0	0	0	0
	181	TCC	0	0	1	2	0	0	0	0	0
	182	CGC	1	0	1	2	1	0	-1	1	-10.6154
	183	TAC	0	0	0.580584	2	0	0	0	0	0
	184	AAC	0	0	0.580584	2.41942	0	0	0	0	0
	185	TAC	0	0	0.580584	2	0	0	0	0	0
	186	GTA	0	0	1	2	0	0	0	0	0
	187	GTT	1	0	1	2	1	0	-1	1	-10.6154
	188	GGG	0	0	1	2	0	0	0	0	0
	189	AAG	0	0	0.555986	2.27732	0	0	0	0	0
190	190	AAT	0	1	0.587483	2.4068	0	0.4155	0.41549	0.8038	4.4106
191	191	ATT	2	1	0.826118	2.17388	2.421	0.46	-1.961	0.9791	-20.8163
192	192	GTT	0	1	0.991171	2.00883	0	0.4978	0.4978	0.6696	5.28438
	193	GAC	0	0	0.580584	2.41942	0	0	0	0	0

	194	GCC	0	0	1	2	0	0	0	0	0
	195	TCC	0	0	1	2	0	0	0	0	0
	196	AAG	0	0	0.555986	2.27732	0	0	0	0	0
	197	TTC	1	0	0.580913	2.41909	1.7214	0	-1.7214	1	-18.2737
	198	GTC	1	0	1	2	1	0	-1	1	-10.6154
	199	AAG	1	0	0.55559	2.27771	1.7999	0	-1.7999	1	-19.1065
	200	GGT	0	0	1	2	0	0	0	0	0
	201	TTG	1	0	1.13823	1.65134	0.8786	0	-0.8786	1	-9.32621
	202	CGC	0	0	1	2	0	0	0	0	0
	203	GTG	0	0	1	2	0	0	0	0	0
	204	ACG	0	0	1	2	0	0	0	0	0
	205	ACG	0	0	1	2	0	0	0	0	0
	206	GAG	0	0	0.555986	2.15047	0	0	0	0	0
	207	TGG	0	0	0	1.88803	0	0	0	0	0
	208	ATG	0	0	0	3	0	0	0	0	0
	209	AAC	0	0	0.580584	2.41942	0	0	0	0	0
	210	ACG	0	0	1	2	0	0	0	0	0
	211	GGT	0	0	1	2	0	0	0	0	0
	212	GAC	0	0	0.580584	2.41942	0	0	0	0	0
	213	GGC	0	0	1	2	0	0	0	0	0
	214	AAG	0	0	0.555986	2.27732	0	0	0	0	0
	215	ATA	0	0	0.473058	2.52694	0	0	0	0	0
	216	GGC	0	0	1	2	0	0	0	0	0
	217	GAT	0	0	0.588602	2.4114	0	0	0	0	0
	218	ATT	0	0	0.742743	2.25726	0	0	0	0	0

	219	AAG	0	0	0.555986	2.27732	0	0	0	0	0
	220	AAG	0	1	0.557102	2.2819	0	0.4382	0.43823	0.8038	4.652
	221	GGT	0	0	1	2	0	0	0	0	0
	222	GCC	0	0	1	2	0	0	0	0	0
	223	CTG	0	0	1.58058	1.41942	0	0	0	0	0
	224	TAT	1	0	0.585695	2	1.7074	0	-1.7074	1	-18.1245
	225	TTA	1	0	1.52611	1.4257	0.6553	0	-0.6553	1	-6.95586
	226	GTA	0	0	1	2	0	0	0	0	0
	227	ACC	0	0	1	2	0	0	0	0	0
	228	TGT	0	0	0.588602	2.25726	0	0	0	0	0
	229	ACT	0	0	1	2	0	0	0	0	0
	230	CGT	0	0	1	2	0	0	0	0	0
	231	GGA	0	0	1	1.70645	0	0	0	0	0
	232	GGT	1	0	1	1.93577	1	0	-1	1	-10.6154
233	233	ATA	1	1	0.828792	2.17121	1.2066	0.4606	-0.746	0.9237	-7.91911
	234	ACT	0	1	1	2	0	0.5	0.5	0.6667	5.3077
	235	GGG	2	0	1	1.80506	2	0	-2	1	-21.2308
	236	GAC	0	0	0.580584	2.41942	0	0	0	0	0
	237	AGT	0	0	0.588602	2.4114	0	0	0	0	0
	238	TCC	0	1	1	2	0	0.5	0.5	0.6667	5.3077
	239	TCC	0	0	1	2	0	0	0	0	0
	240	ATT	0	0	0.742743	2.25726	0	0	0	0	0
	241	TCA	0	0	1	1.58058	0	0	0	0	0
	242	TTC	0	0	0.580584	2.41942	0	0	0	0	0
	243	GAA	0	0	0.526942	2.17951	0	0	0	0	0

	244	GTT	0	0	1	2	0	0	0	0	0
	245	GTA	0	0	1	2	0	0	0	0	0
	246	TGT	0	0	0.588602	2.25726	0	0	0	0	0
	247	GCC	0	0	1	2	0	0	0	0	0
	248	TAT	0	0	0.588602	2	0	0	0	0	0
	249	ACG	0	0	1	2	0	0	0	0	0
	250	CAC	0	0	0.580584	2.41942	0	0	0	0	0
	251	GCG	0	0	1	2	0	0	0	0	0
	252	TGT	0	1	0.568466	2.24463	0	0.4455	0.44551	0.7979	4.72926
	253	TAC	0	0	0.580584	2	0	0	0	0	0
	254	TTC	0	0	0.580584	2.41942	0	0	0	0	0
	255	AAA	0	0	0.526942	2.30636	0	0	0	0	0
	256	TCC	0	0	1	2	0	0	0	0	0
	257	ATT	0	0	0.742743	2.25726	0	0	0	0	0
	258	GGG	0	0	1	2	0	0	0	0	0
	259	ATT	0	0	0.742743	2.25726	0	0	0	0	0
	260	CAA	0	0	0.526942	1.89247	0	0	0	0	0

F.

Coevolutionary sites of intra-protein of BDV-CP and BDV-Rep	Codon#	Triplet	Syn (s)	Nonsyn (n)	Syn sites (S)	Nonsyn sites (N)	d_S	d_N	d_N - d_S	P-value	Normalized d_N - d_S
	1	ATG	0	0	0	3	0	0	0	0	0
	2	GCT	1	0	1	2	1	0	-1	1	-7.0526
	3	TCT	0	0	1	2	0	0	0	0	0
	4	TCA	0	1	1	1.65492	0	0.6043	0.60426	0.6233	4.26159
	5	TCT	0	0	1	2	0	0	0	0	0
	6	GCA	0	0	1	2	0	0	0	0	0
	7	CCC	0	0	1	2	0	0	0	0	0
	8	AGG	0	0	0.729575	2.27042	0	0	0	0	0
	9	TTC	0	0	0.583919	2.41608	0	0	0	0	0
	10	CGT	0	0	1	2	0	0	0	0	0
	11	GTC	0	0	1	2	0	0	0	0	0
	12	TAT	0	0	0.368245	2	0	0	0	0	0
	13	TCG	1	0	1	1.95195	1	0	-1	1	-7.0526
	14	AAA	0	0	0.378273	2.1926	0	0	0	0	0
	15	TAC	0	0	0.583919	2	0	0	0	0	0
	16	CTC	1	0	1.02577	1.97423	0.9749	0	-0.9749	1	-6.8754

	17	TTC	0	0	0.583919	2.41608	0	0	0	0	0
	18	CTA	0	0	1.58392	1.41608	0	0	0	0	0
	19	ACA	0	0	1	2	0	0	0	0	0
	20	TAT	0	0	0.368245	2	0	0	0	0	0
	21	CCT	0	0	1	2	0	0	0	0	0
	22	CAA	0	0	0.378273	2.03781	0	0	0	0	0
	23	TGC	0	0	0.583919	2.13799	0	0	0	0	0
	24	ATC	0	0	0.86201	2.13799	0	0	0	0	0
	25	CTT	1	0	1.48303	1.51697	0.6743	0	-0.6743	1	-4.75553
	26	GAA	1	0	0.509555	2.15723	1.9625	0	-1.9625	1	-13.8407
	27	CCA	0	0	1	2	0	0	0	0	0
	28	CAG	0	0	0.536975	1.87911	0	0	0	0	0
	29	TAC	0	0	0.583919	2	0	0	0	0	0
	30	GCC	0	0	1	2	0	0	0	0	0
	31	TTG	0	0	0.90522	1.68641	0	0	0	0	0
32	32	GAG	0	1	0.575808	2.36662	0	0.4225	0.42254	0.8043	2.98003
	33	TCT	0	0	1	2	0	0	0	0	0
	34	CTT	0	0	1	2	0	0	0	0	0
	35	CGC	0	0	1	2	0	0	0	0	0
	36	ACT	0	0	1	2	0	0	0	0	0
	37	CTC	0	0	1	2	0	0	0	0	0
	38	TTG	0	0	0.90522	1.68641	0	0	0	0	0
	39	GCA	1	0	1	2	1	0	-1	1	-7.0526
	40	AAA	1	1	0.383013	2.19077	2.6109	0.4565	-2.1544	0.9779	-15.1942
	41	TAT	1	0	0.371178	2	2.6941	0	-2.6941	1	-19.0006

	42	GAG	1	0	0.405693	2.26109	2.4649	0	-2.4649	1	-17.3841
	43	CCT	0	0	1	2	0	0	0	0	0
	44	CTC	1	0	1.48303	1.51697	0.6743	0	-0.6743	1	-4.75553
	45	TAC	0	0	0.583919	2	0	0	0	0	0
	46	ATC	1	0	0.663241	2.33676	1.5078	0	-1.5078	1	-10.6335
	47	GCT	0	0	1	2	0	0	0	0	0
	48	GCA	1	0	1	2	1	0	-1	1	-7.0526
	49	GTT	0	0	1	2	0	0	0	0	0
	50	CGA	2	0	0.818443	2.08643	2.4437	0	-2.4437	1	-17.2342
	51	GAG	1	0	0.405693	2.26109	2.4649	0	-2.4649	1	-17.3841
	52	CTT	1	0	1	2	1	0	-1	1	-7.0526
	53	CAC	0	0	0.583919	2.41608	0	0	0	0	0
	54	GAA	0	0	0.378273	2.28851	0	0	0	0	0
	55	GAT	0	1	0.368245	2.63175	0	0.38	0.37998	0.8773	2.67981
	56	GGA	0	0	1	1.66678	0	0	0	0	0
	57	TCA	0	0	1	1.58392	0	0	0	0	0
	58	CCA	0	0	1	2	0	0	0	0	0
	59	CAT	0	0	0.368245	2.63175	0	0	0	0	0
	60	CTG	1	0	1.58392	1.41608	0.6313	0	-0.6313	1	-4.45263
	61	CAC	0	0	0.583919	2.41608	0	0	0	0	0
	62	GTA	0	0	1	2	0	0	0	0	0
	63	CTC	0	1	0.997316	2.00268	0	0.4993	0.49933	0.6676	3.52158
	64	GTG	0	1	0.980546	2.01945	0	0.4952	0.49518	0.6732	3.49233
	65	CAA	0	0	0.378273	2.03781	0	0	0	0	0
	66	AAC	0	1	0.583598	2.41347	0	0.4143	0.41434	0.8053	2.92218

	67	AAG	0	0	0.536975	2.0339	0	0	0	0	0
	68	CTT	0	0	1	2	0	0	0	0	0
	69	CGT	0	0	1	2	0	0	0	0	0
	70	GCT	0	0	1	2	0	0	0	0	0
	71	TCG	1	0	1	1.95195	1	0	-1	1	-7.0526
	72	ATC	0	0	0.86201	2.13799	0	0	0	0	0
	73	ACC	0	0	1	2	0	0	0	0	0
	74	AAT	0	0	0.368245	2.63175	0	0	0	0	0
	75	CCC	0	0	1	2	0	0	0	0	0
	76	AAC	1	0	0.405508	2.59449	2.466	0	-2.466	1	-17.392
	77	GCC	1	0	1	2	1	0	-1	1	-7.0526
	78	CTA	1	0	1.10089	1.89911	0.9084	0	-0.9084	1	-6.4063
	79	AAC	0	0	0.583919	2.41608	0	0	0	0	0
80	80	CTT	0	1	0.815212	2.18479	0	0.4577	0.45771	0.7283	3.22805
81	81	TGT	0	1	0.89085	2.03859	0	0.4905	0.49053	0.6959	3.45954
	82	ATG	0	0	0	3	0	0	0	0	0
83	83	GAC	0	1	0.583919	2.41608	0	0.4139	0.41389	0.8054	2.91903
	84	ACC	2	0	1	2	2	0	-2	1	-14.1052
	85	TCT	1	0	1	1.71255	1	0	-1	1	-7.0526
	86	CCA	0	0	1	2	0	0	0	0	0
	87	TTT	0	0	0.368245	2.63175	0	0	0	0	0
	88	TCT	2	1	1	1.66051	2	0.6022	-1.3978	0.9469	-9.85795
89	89	ATA	0.5	1.5	0.431865	2.54464	1.1578	0.5895	-0.5683	0.8549	-4.00797
	90	TTC	1	0	0.405508	2.59449	2.466	0	-2.466	1	-17.392
	91	CAT	0	0	0.368245	2.63175	0	0	0	0	0

	92	CCT	0	0	1	2	0	0	0	0	0
	93	AAT	1	0	0.546656	2.45334	1.8293	0	-1.8293	1	-12.9014
	94	ATT	0	0	0.776618	2.22338	0	0	0	0	0
	95	CAA	0	0	0.378273	2.03781	0	0	0	0	0
	96	GCT	0	0	1	2	0	0	0	0	0
	97	GCC	0	0	1	2	0	0	0	0	0
	98	AAA	1	0	0.37935	2.19152	2.6361	0	-2.6361	1	-18.5913
	99	GAT	1	0	0.546656	2.45334	1.8293	0	-1.8293	1	-12.9014
	100	TGC	0	0	0.583919	2.13799	0	0	0	0	0
	101	AAC	0	0	0.583919	2.41608	0	0	0	0	0
	102	CAA	0	0	0.378273	2.03781	0	0	0	0	0
	103	GTT	0	0	1	2	0	0	0	0	0
	104	CGT	0	0	1	2	0	0	0	0	0
	105	GAT	0	0	0.368245	2.63175	0	0	0	0	0
	106	TAC	0	0	0.583919	2	0	0	0	0	0
	107	ATC	0	0	0.86201	2.13799	0	0	0	0	0
	108	ACG	0	2	0.176163	2.82384	0	0.7083	0.70826	0.886	4.99505
	109	AAG	0	0	0.536975	2.0339	0	0	0	0	0
	110	GAG	0	0	0.536975	2.12981	0	0	0	0	0
	111	GTT	0	0	1	2	0	0	0	0	0
	112	GAC	0	0	0.583919	2.41608	0	0	0	0	0
	113	TCC	0	1	0.996737	2.00326	0	0.4992	0.49919	0.6678	3.52056
	114	GAT	0	0	0.368245	2.63175	0	0	0	0	0
	115	GTC	0	0	1	2	0	0	0	0	0
	116	AAC	0	0	0.583919	2.41608	0	0	0	0	0

	117	ACG	0	0	1	2	0	0	0	0	0
	118	GCT	0	0	1	2	0	0	0	0	0
	119	GAG	0	0	0.536975	2.12981	0	0	0	0	0
	120	TGG	0	0	0	1.92605	0	0	0	0	0
	121	GGA	0	0	1	1.66678	0	0	0	0	0
	122	ACA	0	0	1	2	0	0	0	0	0
	123	TTC	0	0	0.583919	2.41608	0	0	0	0	0
124	124	ATA	0	1	0.934644	2.06536	0	0.4842	0.48418	0.6885	3.41472
	125	GCA	0	0	1	2	0	0	0	0	0
	126	GTT	0	0	1	2	0	0	0	0	0
	127	ACC	0	0	1	2	0	0	0	0	0
	128	ACT	0	0	1	2	0	0	0	0	0
	129	CCG	0	0	1	2	0	0	0	0	0
	130	GGT	0	0	1	2	0	0	0	0	0
	131	CGT	0	0	1	2	0	0	0	0	0
	132	AAA	0	0	0.378273	2.1926	0	0	0	0	0
	133	GAC	0	0	0.583919	2.41608	0	0	0	0	0
	134	CGA	2	2	1.15903	1.38829	1.7256	1.4406	-0.285	0.7518	-2.00971
	135	GAC	1	2	0.576916	2.39328	1.7334	0.8357	-0.8977	0.9015	-6.33099
	136	GCG	0.6667	2.33333	0.9434	2.03708	0.7067	1.1454	0.43877	0.615	3.09447
	137	GAT	0	2	0.404181	2.55988	0	0.7813	0.78129	0.7459	5.5101
	138	ATG	0	2	0.137561	2.83921	0	0.7044	0.70442	0.9097	4.96801
	139	AAA	0.25	2.75	0.412011	2.24176	0.6068	1.2267	0.61994	0.6859	4.37218
	140	CAG	1.5	2.5	0.568313	1.97505	2.6394	1.2658	-1.3736	0.8725	-9.68746
	141	ATC	1	1	0.785161	2.21484	1.2736	0.4515	-0.8221	0.9315	-5.79812

	142	ATT	0	1	0.70693	2.29307	0	0.4361	0.4361	0.7644	3.07562
	143	GAG	0.25	2.75	0.559143	2.17075	0.4471	1.2668	0.81973	0.5999	5.78124
	144	TCG	0.5	1.5	0.922737	1.83922	0.5419	0.8156	0.2737	0.6659	1.93029
	145	AGT	0	2	0.391475	2.5853	0	0.7736	0.77361	0.7543	5.45594
	146	ACC	1	2	0.923984	2.07602	1.0823	0.9634	-0.1189	0.7739	-0.838455
	147	TCA	1.5	1.5	0.922737	1.83768	1.6256	0.8162	-0.8094	0.8511	-5.70803
	148	AGA	0.25	2.75	0.555312	2.10322	0.4502	1.3075	0.85732	0.5932	6.04635
	149	GAG	0.25	2.75	0.559143	2.17075	0.4471	1.2668	0.81973	0.5999	5.78124
	150	GAA	0.25	2.75	0.437679	2.28745	0.5712	1.2022	0.63102	0.6763	4.45033
	151	TTT	0	0	0.368245	2.63175	0	0	0	0	0
	152	CTC	1	1	0.90446	2.09554	1.1056	0.4772	-0.6284	0.9091	-4.43205
	153	AGC	1	2	0.566638	2.41384	1.7648	0.8286	-0.9362	0.9053	-6.60292
	154	ATG	0	0	0	3	0	0	0	0	0
	155	GTC	0	0	1	2	0	0	0	0	0
	156	TGC	0	0	0.583919	2.13799	0	0	0	0	0
	157	CAT	0	0	0.368245	2.63175	0	0	0	0	0
	158	CGT	0	0	1	2	0	0	0	0	0
	159	TTT	0	0	0.368245	2.63175	0	0	0	0	0
	160	CCG	1	0	1	2	1	0	-1	1	-7.0526
	161	TTT	1	1	0.397156	2.60284	2.5179	0.3842	-2.1337	0.9825	-15.0482
	162	GAA	0.5	1.5	0.406635	2.2784	1.2296	0.6584	-0.5713	0.8486	-4.0288
	163	TGG	0	0	0	1.92605	0	0	0	0	0
	164	TCT	1	0	1	2	1	0	-1	1	-7.0526
	165	ATC	0	2	0.86957	2.13043	0	0.9388	0.93878	0.5043	6.62083
	166	CGT	0	0	1	2	0	0	0	0	0

	167	CTG	1	0	1.5363	1.4637	0.6509	0	-0.6509	1	-4.59064
	168	AAA	0	0	0.378273	2.1926	0	0	0	0	0
	169	GAC	0	1	0.589582	2.41042	0	0.4149	0.41487	0.8035	2.92588
	170	TTC	0	1	0.586743	2.41326	0	0.4144	0.41438	0.8044	2.92244
	171	GAG	1	0	0.405693	2.26109	2.4649	0	-2.4649	1	-17.3841
	172	TAC	0	0	0.583919	2	0	0	0	0	0
	173	ACG	1	0	1	2	1	0	-1	1	-7.0526
	174	GCA	0	0	1	2	0	0	0	0	0
	175	CGC	0	0	1	2	0	0	0	0	0
	176	CAC	1	0	0.582455	2.41755	1.7169	0	-1.7169	1	-12.1084
	177	TTA	1	0	1.43924	1.45161	0.6948	0	-0.6948	1	-4.90023
	178	TTC	0	0	0.583919	2.41608	0	0	0	0	0
	179	CCA	0	0	1	2	0	0	0	0	0
	180	GAC	0	0	0.583919	2.41608	0	0	0	0	0
	181	CCA	0	0	1	2	0	0	0	0	0
	182	GTT	0	0	1	2	0	0	0	0	0
183	183	AAC	0	2	0.585899	2.4141	0	0.8285	0.82847	0.6475	5.84284
	184	ACT	1	0	1	2	1	0	-1	1	-7.0526
	185	TAC	0	0	0.583919	2	0	0	0	0	0
	186	ACA	0	1	1	2	0	0.5	0.5	0.6667	3.5263
	187	CCT	0	0	1	2	0	0	0	0	0
	188	GAG	1	0	0.533193	2.13359	1.8755	0	-1.8755	1	-13.2271
	189	TTT	0	0	0.368245	2.63175	0	0	0	0	0
	190	CCT	1	0	1	2	1	0	-1	1	-7.0526
	191	ATC	0	0	0.86201	2.13799	0	0	0	0	0

	192	GAG	0	0	0.536975	2.12981	0	0	0	0	0
	193	TCC	0	0	1	2	0	0	0	0	0
	194	CTC	0	0	1	2	0	0	0	0	0
	195	ATG	0	1	0.00845381	2.99155	0	0.3343	0.33428	0.9972	2.35751
	196	TGT	0	0	0.368245	2.22338	0	0	0	0	0
	197	CAT	0	1	0.372557	2.62744	0	0.3806	0.3806	0.8758	2.68421
	198	GAG	0	0	0.536975	2.12981	0	0	0	0	0
	199	ACC	0	0	1	2	0	0	0	0	0
	200	ATT	0	0	0.776618	2.22338	0	0	0	0	0
	201	GAA	0	0	0.378273	2.28851	0	0	0	0	0
	202	AGC	0	0	0.583919	2.41608	0	0	0	0	0
	203	TGG	0	0	0	1.92605	0	0	0	0	0
	204	AAA	0	0	0.378273	2.1926	0	0	0	0	0
	205	AAT	0	1	0.385024	2.61498	0	0.3824	0.38241	0.8717	2.69701
	206	GAA	0	3	0.376965	2.3333	0	1.2857	1.28573	0.6381	9.06777
	207	CAT	0	0	0.368245	2.63175	0	0	0	0	0
	208	CTA	2	0	1.45457	1.54543	1.375	0	-1.375	1	-9.69717
	209	TAC	0	0	0.583919	2	0	0	0	0	0
	210	TCC	0	0	1	2	0	0	0	0	0
	211	GAG	0	0	0.536975	2.12981	0	0	0	0	0
	212	TCA	0	0	1	1.58392	0	0	0	0	0
	213	CCG	0	0	1	2	0	0	0	0	0
	214	GGA	0	0	1	1.66678	0	0	0	0	0
	215	CGG	1	0	1.27063	1.72937	0.787	0	-0.787	1	-5.5505
	216	CAT	0	0	0.368245	2.63175	0	0	0	0	0

	217	AAG	0	0	0.536975	2.0339	0	0	0	0	0
	218	TCC	0	0	1	2	0	0	0	0	0
	219	ATC	1	2	0.843505	2.1451	1.1855	0.9324	-0.2532	0.806	-1.78551
	220	TAC	0	0	0.583919	2	0	0	0	0	0
	221	ATC	0	0	0.86201	2.13799	0	0	0	0	0
	222	TGC	0	0	0.583919	2.13799	0	0	0	0	0
	223	GGT	0	0	1	2	0	0	0	0	0
	224	CCG	0	0	1	2	0	0	0	0	0
	225	ACA	0	0	1	2	0	0	0	0	0
	226	AGA	0	0	0.570874	2	0	0	0	0	0
	227	ACA	0	0	1	2	0	0	0	0	0
	228	GGA	0	0	1	1.66678	0	0	0	0	0
	229	AAG	1	0	0.530454	2.04042	1.8852	0	-1.8852	1	-13.2954
	230	ACT	0	0	1	2	0	0	0	0	0
	231	TCC	0	0	1	2	0	0	0	0	0
	232	TGG	0	0	0	1.92605	0	0	0	0	0
	233	GCA	0	0	1	2	0	0	0	0	0
	234	AGA	0	0	0.570874	2	0	0	0	0	0
	235	TCT	0	0	1	2	0	0	0	0	0
	236	CTA	0	0	1.58392	1.41608	0	0	0	0	0
	237	GGG	0	0	1	2	0	0	0	0	0
	238	ATC	0	0	0.86201	2.13799	0	0	0	0	0
	239	CAC	0	0	0.583919	2.41608	0	0	0	0	0
	240	AAC	0	0	0.583919	2.41608	0	0	0	0	0
	241	TAC	0	0	0.583919	2	0	0	0	0	0

	242	TAC	0	0	0.583919	2	0	0	0	0	0
	243	AAC	0	0	0.583919	2.41608	0	0	0	0	0
	244	AGC	0	1	0.586744	2.41326	0	0.4144	0.41438	0.8044	2.92244
	245	CTA	0	0	1.58392	1.41608	0	0	0	0	0
	246	GCT	1	1	1	2	1	0.5	-0.5	0.8889	-3.5263
	247	GAT	0	0	0.368245	2.63175	0	0	0	0	0
	248	TTC	0	1	0.583919	2.40617	0	0.4156	0.4156	0.8047	2.93106
	249	ACG	0	0	1	2	0	0	0	0	0
	250	ACC	0	0	1	2	0	0	0	0	0
	251	TAT	0	0	0.368245	2	0	0	0	0	0
	252	GAC	0	0	0.583919	2.41608	0	0	0	0	0
	253	GTC	0	0	1	2	0	0	0	0	0
	254	AAC	0	0	0.583919	2.41608	0	0	0	0	0
	255	GCC	1	0	1	2	1	0	-1	1	-7.0526
	256	AAG	0	0	0.536975	2.0339	0	0	0	0	0
	257	TAC	0	0	0.583919	2	0	0	0	0	0
	258	AAC	0	0	0.583919	2.41608	0	0	0	0	0
	259	ATA	0	0	0.621727	2.37827	0	0	0	0	0
	260	ATT	0	0	0.776618	2.22338	0	0	0	0	0
	261	GAC	0	0	0.583919	2.41608	0	0	0	0	0
	262	GAC	0	0	0.583919	2.41608	0	0	0	0	0
	263	ATT	0	0	0.776618	2.22338	0	0	0	0	0
	264	CCA	0	0	1	2	0	0	0	0	0
	265	TTC	0	0	0.583919	2.41608	0	0	0	0	0
	266	AAG	0	1	0.537453	2.0378	0	0.4907	0.49073	0.7913	3.4609

	267	TTC	0	1	0.588161	2.41184	0	0.4146	0.41462	0.8039	2.92416
	268	ACG	0	0	1	2	0	0	0	0	0
	269	CCA	0	0	1	2	0	0	0	0	0
	270	AAC	0	0	0.583919	2.41608	0	0	0	0	0
	271	TGG	0	0	0	1.92605	0	0	0	0	0
	272	AAG	0	0	0.536975	2.0339	0	0	0	0	0
	273	TGC	0	0	0.583919	2.13799	0	0	0	0	0
	274	TTC	0	0	0.583919	2.41608	0	0	0	0	0
	275	GTC	0	0	1	2	0	0	0	0	0
	276	GGG	1	0	1	1.99206	1	0	-1	1	-7.0526
	277	GCT	0	0	1	2	0	0	0	0	0
	278	CAG	0	0	0.536975	1.87911	0	0	0	0	0
	279	CGT	1	0	1.00663	1.97946	0.9934	0	-0.9934	1	-7.00617
	280	GAC	0	0	0.583919	2.41608	0	0	0	0	0
	281	TTC	0	0	0.583919	2.41608	0	0	0	0	0
	282	ACC	0	1	1	2	0	0.5	0.5	0.6667	3.5263
	283	GTC	0	0	1	2	0	0	0	0	0
	284	AAT	0	0	0.368245	2.63175	0	0	0	0	0
	285	CCT	0	0	1	2	0	0	0	0	0
	286	AAG	0	0	0.536975	2.0339	0	0	0	0	0
	287	TAC	0	0	0.583919	2	0	0	0	0	0
	288	GGG	1	0	1	1.9966	1	0	-1	1	-7.0526
	289	AAG	0	0	0.536975	2.0339	0	0	0	0	0
	290	CGA	0	0	1.27809	1.13799	0	0	0	0	0
	291	AAA	0	0	0.378273	2.1926	0	0	0	0	0

	292	ATG	0	0	0	3	0	0	0	0	0
	293	ATA	0	0	0.621727	2.37827	0	0	0	0	0
	294	CGG	0	0	1.27809	1.72191	0	0	0	0	0
	295	GGT	0	0	1	2	0	0	0	0	0
	296	GGT	0	0	1	2	0	0	0	0	0
	297	ATA	0	0	0.621727	2.37827	0	0	0	0	0
	298	CCT	0	0	1	2	0	0	0	0	0
	299	TGC	0	0	0.583919	2.13799	0	0	0	0	0
	300	ATC	0	0	0.86201	2.13799	0	0	0	0	0
	301	ATT	0	0	0.776618	2.22338	0	0	0	0	0
	302	TTA	0	0	0.746518	1.62173	0	0	0	0	0
	303	GTT	0	0	1	2	0	0	0	0	0
	304	AAC	0	0	0.583919	2.41608	0	0	0	0	0
	305	CCA	0	0	1	2	0	0	0	0	0
	306	GAC	0	0	0.583919	2.41608	0	0	0	0	0
	307	GAA	0	0	0.378273	2.28851	0	0	0	0	0
	308	GAT	0	0	0.368245	2.63175	0	0	0	0	0
	309	TGG	0	0	0	1.92605	0	0	0	0	0
	310	CTC	0	0	1	2	0	0	0	0	0
	311	GAG	0	1	0.536975	2.03497	0	0.4914	0.49141	0.7912	3.46571
	312	GAT	0	0	0.368245	2.63175	0	0	0	0	0
	313	ATG	0	0	0	3	0	0	0	0	0
	314	ACT	0	0	1	2	0	0	0	0	0
	315	CCT	0	0	1	2	0	0	0	0	0
	316	GAA	0	0	0.378273	2.28851	0	0	0	0	0

	317	CAG	0	0	0.536975	1.87911	0	0	0	0	0
	318	TCG	0	0	1	1.72191	0	0	0	0	0
	319	GAT	0	0	0.368245	2.63175	0	0	0	0	0
	320	TAC	0	0	0.583919	2	0	0	0	0	0
	321	ATG	0	0	0	3	0	0	0	0	0
	322	TAC	0	0	0.583919	2	0	0	0	0	0
	323	TCT	0	0	1	2	0	0	0	0	0
	324	AAC	0	0	0.583919	2.41608	0	0	0	0	0
	325	GCT	0	0	1	2	0	0	0	0	0
	326	GTG	0	0	1	2	0	0	0	0	0
	327	ATA	0	0	0.621727	2.37827	0	0	0	0	0
	328	CAT	0	0	0.368245	2.63175	0	0	0	0	0
	329	TAT	0	0	0.368245	2	0	0	0	0	0
	330	ATG	0	0	0	3	0	0	0	0	0
	331	TAT	0	0	0.368245	2	0	0	0	0	0
	332	GAG	0	0	0.536975	2.12981	0	0	0	0	0
	333	GGT	0	0	1	2	0	0	0	0	0
	334	GAA	0	0	0.378273	2.28851	0	0	0	0	0
	335	TCA	0	0	1	1.58392	0	0	0	0	0
	336	TTC	0	0	0.583919	2.41608	0	0	0	0	0
	337	TTC	0	0	0.583919	2.41608	0	0	0	0	0
	338	GCC	0	0	1	2	0	0	0	0	0
	339	TTC	0	0	0.583919	2.41608	0	0	0	0	0
	340	GGC	0	0	1	2	0	0	0	0	0
	341	GAA	0	1	0.380381	2.2864	0	0.4374	0.43737	0.8574	3.08458

	342	AAT	0	0	0.368245	2.63175	0	0	0	0	0
	343	GTC	0	0	1	2	0	0	0	0	0
	344	ACT	0	0	1	2	0	0	0	0	0
	345	GCT	0	0	1	2	0	0	0	0	0
	346	TCG	0	0	1	1.72191	0	0	0	0	0
	347	CAG	0	0	0.536975	1.87911	0	0	0	0	0

G.

Coevolutionary sites of inter-protein of BDV-CP and BDV-Rep A	Coevolutionary sites of intra-protein	Codon#	Triplet	Syn (s)	Nonsyn (n)	Syn sites (S)	Nonsyn sites (N)	d_S	d_N	d_N - d_S	P-value	Normalized d_N - d_S
		1	ATG	0	0	0	3	0	0	0	0	0
2		2	GCT	1	0	1	2	1	0	-1	1	-5.59999
		3	TCT	0	0	1	2	0	0	0	0	0
		4	TCA	0	1	1	1.65767	0	0.6033	0.60326	0.6237	3.37823
		5	TCT	0	0	1	2	0	0	0	0	0
6		6	GCA	0	0	1	2	0	0	0	0	0
		7	CCC	0	0	1	2	0	0	0	0	0
		8	AGG	0	0	0.750493	2.24951	0	0	0	0	0
		9	TTC	0	0	0.582451	2.41755	0	0	0	0	0
		10	CGT	0	0	1	2	0	0	0	0	0
		11	GTC	0	0	1	2	0	0	0	0	0
		12	TAT	0	0	0.390699	2	0	0	0	0	0
		13	TCG	1	0	1	1.95333	1	0	-1	1	-5.59999
		14	AAA	0	0	0.397158	2.20753	0	0	0	0	0
		15	TAC	0	0	0.582451	2	0	0	0	0	0
		16	CTC	1	0	1.02064	1.97936	0.9798	0	-0.9798	1	-5.48672
		17	TTC	0	0	0.582451	2.41755	0	0	0	0	0

		18	CTA	0	0	1.58245	1.41755	0	0	0	0	0
		19	ACA	0	0	1	2	0	0	0	0	0
		20	TAT	0	0	0.390699	2	0	0	0	0	0
		21	CCT	0	0	1	2	0	0	0	0	0
		22	CAA	0	0	0.397158	2.02039	0	0	0	0	0
		23	TGC	0	0	0.582451	2.13813	0	0	0	0	0
24		24	ATC	0	0	0.861875	2.13813	0	0	0	0	0
		25	CTT	1	0	1.48517	1.51483	0.6733	0	-0.6733	1	-3.7706
		26	GAA	1	0	0.518611	2.15601	1.9282	0	-1.9282	1	-10.7981
		27	CCA	0	0	1	2	0	0	0	0	0
		28	CAG	0	0	0.542963	1.87459	0	0	0	0	0
29		29	TAC	0	0	0.582451	2	0	0	0	0	0
		30	GCC	0	0	1	2	0	0	0	0	0
		31	TTG	0	0	0.933662	1.68601	0	0	0	0	0
32	32	32	GAG	0	1	0.575856	2.3698	0	0.422	0.42198	0.8045	2.36306
		33	TCT	0	0	1	2	0	0	0	0	0
		34	CTT	0	0	1	2	0	0	0	0	0
		35	CGC	0	0	1	2	0	0	0	0	0
		36	ACT	0	0	1	2	0	0	0	0	0
		37	CTC	0	0	1	2	0	0	0	0	0
		38	TTG	0	0	0.933662	1.68601	0	0	0	0	0
39		39	GCA	1	0	1	2	1	0	-1	1	-5.59999
		40	AAA	1	1	0.401704	2.20579	2.4894	0.4534	-2.036	0.9763	-11.4018
		41	TAT	1	0	0.393422	2	2.5418	0	-2.5418	1	-14.234
		42	GAG	1	0	0.42151	2.25311	2.3724	0	-2.3724	1	-13.2855

		43	CCT	0	0	1	2	0	0	0	0	0
		44	CTC	1	0	1.48517	1.51483	0.6733	0	-0.6733	1	-3.7706
		45	TAC	0	0	0.582451	2	0	0	0	0	0
		46	ATC	1	0	0.646106	2.35389	1.5477	0	-1.5477	1	-8.66729
		47	GCT	0	0	1	2	0	0	0	0	0
		48	GCA	1	0	1	2	1	0	-1	1	-5.59999
		49	GTT	0	0	1	2	0	0	0	0	0
		50	CGA	2	0	0.833134	2.0769	2.4006	0	-2.4006	1	-13.4432
		51	GAG	1	0	0.42151	2.25311	2.3724	0	-2.3724	1	-13.2855
		52	CTT	1	0	1	2	1	0	-1	1	-5.59999
		53	CAC	0	0	0.582451	2.41755	0	0	0	0	0
		54	GAA	0	0	0.397158	2.27746	0	0	0	0	0
		55	GAT	0	1	0.390699	2.6093	0	0.3832	0.38324	0.8698	2.14616
		56	GGA	0	0	1	1.67462	0	0	0	0	0
		57	TCA	0	0	1	1.58245	0	0	0	0	0
		58	CCA	0	0	1	2	0	0	0	0	0
		59	CAT	0	0	0.390699	2.6093	0	0	0	0	0
		60	CTG	1	0	1.58245	1.41755	0.6319	0	-0.6319	1	-3.53881
		61	CAC	0	0	0.582451	2.41755	0	0	0	0	0
		62	GTA	0	0	1	2	0	0	0	0	0
		63	CTC	0	1	0.997074	2.00293	0	0.4993	0.49927	0.6676	2.7959
		64	GTG	0	1	0.978816	2.02118	0	0.4948	0.49476	0.6737	2.77065
		65	CAA	0	0	0.397158	2.02039	0	0	0	0	0
		66	AAC	0	1	0.582031	2.41376	0	0.4143	0.41429	0.8057	2.32002
		67	AAG	0	0	0.542963	2.06172	0	0	0	0	0

		68	CTT	0	0	1	2	0	0	0	0	0
		69	CGT	0	0	1	2	0	0	0	0	0
		70	GCT	0	0	1	2	0	0	0	0	0
		71	TCG	1	0	1	1.95333	1	0	-1	1	-5.59999
		72	ATC	0	0	0.861875	2.13813	0	0	0	0	0
		73	ACC	0	0	1	2	0	0	0	0	0
		74	AAT	0	0	0.390699	2.6093	0	0	0	0	0
		75	CCC	0	0	1	2	0	0	0	0	0
		76	AAC	1	0	0.422726	2.57727	2.3656	0	-2.3656	1	-13.2473
		77	GCC	1	0	1	2	1	0	-1	1	-5.59999
		78	CTA	1	0	1.09728	1.90272	0.9113	0	-0.9113	1	-5.10351
		79	AAC	0	0	0.582451	2.41755	0	0	0	0	0
	80	80	CTT	0	1	0.809266	2.19073	0	0.4565	0.45647	0.7302	2.55621
	81	81	TGT	0	1	0.898235	2.03824	0	0.4906	0.49062	0.6941	2.74746
		82	ATG	0	0	0	3	0	0	0	0	0
	83	83	GAC	0	1	0.582451	2.41755	0	0.4136	0.41364	0.8059	2.31639
		84	ACC	2	0	1	2	2	0	-2	1	-11.2
		85	TCT	1	0	1	1.71167	1	0	-1	1	-5.59999
		86	CCA	0	0	1	2	0	0	0	0	0
		87	TTT	0	0	0.390699	2.6093	0	0	0	0	0
		88	TCT	2	1	1	1.65711	2	0.6035	-1.3965	0.9467	-7.8206
	89	89	ATA	0.5	1.5	0.448238	2.52794	1.1155	0.5934	-0.5221	0.8494	-2.92381
		90	TTC	1	0	0.422726	2.57727	2.3656	0	-2.3656	1	-13.2473
		91	CAT	0	0	0.390699	2.6093	0	0	0	0	0
		92	CCT	0	0	1	2	0	0	0	0	0

		93	AAT	1	0	0.550425	2.44958	1.8168	0	-1.8168	1	-10.1739
		94	ATT	0	0	0.771022	2.22898	0	0	0	0	0
		95	CAA	0	0	0.397158	2.02039	0	0	0	0	0
		96	GCT	0	0	1	2	0	0	0	0	0
		97	GCC	0	0	1	2	0	0	0	0	0
		98	AAA	1	0	0.398709	2.20598	2.5081	0	-2.5081	1	-14.0453
		99	GAT	1	0	0.550425	2.44958	1.8168	0	-1.8168	1	-10.1739
		100	TGC	0	0	0.582451	2.13813	0	0	0	0	0
		101	AAC	0	0	0.582451	2.41755	0	0	0	0	0
		102	CAA	0	0	0.397158	2.02039	0	0	0	0	0
		103	GTT	0	0	1	2	0	0	0	0	0
		104	CGT	0	0	1	2	0	0	0	0	0
		105	GAT	0	0	0.390699	2.6093	0	0	0	0	0
		106	TAC	0	0	0.582451	2	0	0	0	0	0
		107	ATC	0	0	0.861875	2.13813	0	0	0	0	0
108		108	ACG	0	2	0.170562	2.82944	0	0.7069	0.70685	0.8895	3.95837
109		109	AAG	0	0	0.542963	2.06172	0	0	0	0	0
		110	GAG	0	0	0.542963	2.13166	0	0	0	0	0
		111	GTT	0	0	1	2	0	0	0	0	0
		112	GAC	0	0	0.582451	2.41755	0	0	0	0	0
		113	TCC	0	1	0.996906	2.00309	0	0.4992	0.49923	0.6677	2.79567
		114	GAT	0	0	0.390699	2.6093	0	0	0	0	0
		115	GTC	0	0	1	2	0	0	0	0	0
		116	AAC	0	0	0.582451	2.41755	0	0	0	0	0
		117	ACG	0	0	1	2	0	0	0	0	0

		118	GCT	0	0	1	2	0	0	0	0	0
		119	GAG	0	0	0.542963	2.13166	0	0	0	0	0
		120	TGG	0	0	0	1.91407	0	0	0	0	0
		121	GGA	0	0	1	1.67462	0	0	0	0	0
		122	ACA	0	0	1	2	0	0	0	0	0
		123	TTC	0	0	0.582451	2.41755	0	0	0	0	0
	124	124	ATA	0	1	0.933667	2.06633	0	0.4839	0.48395	0.6888	2.71011
		125	GCA	0	0	1	2	0	0	0	0	0
		126	GTT	0	0	1	2	0	0	0	0	0
		127	ACC	0	0	1	2	0	0	0	0	0
		128	ACT	0	0	1	2	0	0	0	0	0
		129	CCG	0	0	1	2	0	0	0	0	0
		130	GGT	0	0	1	2	0	0	0	0	0
		131	CGT	0	0	1	2	0	0	0	0	0
132		132	AAA	0	0	0.397158	2.20753	0	0	0	0	0
		133	GAC	0	0	0.582451	2.41755	0	0	0	0	0
		134	CGA	2	2	1.15677	1.4	1.729	1.4286	-0.3004	0.7553	-1.68213
		135	GAC	1	2	0.578738	2.39043	1.7279	0.8367	-0.8912	0.9008	-4.99086
		136	GCG	0.6667	2.33333	0.943128	2.03706	0.7069	1.1454	0.43857	0.6151	2.456
		137	GAT	0	2	0.427286	2.53613	0	0.7886	0.7886	0.7324	4.41617
		138	ATG	0	2	0.149283	2.82788	0	0.7072	0.70724	0.9022	3.96055
		139	AAA	0.25	2.75	0.431038	2.25303	0.58	1.2206	0.64058	0.6763	3.58726
		140	CAG	2.5	2.5	0.587342	1.96531	4.2565	1.2721	-2.9844	0.9525	-16.7126
		141	ATC	1	1	0.783055	2.21694	1.2771	0.4511	-0.826	0.9319	-4.62547
		142	ATT	0	1	0.70251	2.29749	0	0.4353	0.43526	0.7658	2.43744

		143	GAG	0.25	2.75	0.568926	2.17133	0.4394	1.2665	0.82708	0.5953	4.63163
		144	TCG	0.5	1.5	0.922843	1.84201	0.5418	0.8143	0.27252	0.6662	1.52613
		145	AGT	0	2	0.413537	2.56363	0	0.7801	0.78015	0.7415	4.3688
146		146	ACC	1	2	0.921562	2.07844	1.0851	0.9623	-0.1229	0.7749	-0.68798
		147	TCA	1.5	1.5	0.922843	1.8415	1.6254	0.8146	-0.8109	0.8514	-4.54081
		148	AGA	0.25	2.75	0.582491	2.10673	0.4292	1.3053	0.87615	0.5805	4.90642
		149	GAG	0.25	2.75	0.568926	2.17133	0.4394	1.2665	0.82708	0.5953	4.63163
		150	GAA	0.25	2.75	0.459236	2.27587	0.5444	1.2083	0.66395	0.6633	3.7181
		151	TTT	0	0	0.390699	2.6093	0	0	0	0	0
		152	CTC	1	1	0.901754	2.09825	1.109	0.4766	-0.6324	0.9096	-3.54122
		153	AGC	1	2	0.567717	2.41248	1.7614	0.829	-0.9324	0.905	-5.22153
		154	ATG	0	0	0	3	0	0	0	0	0
		155	GTC	0	0	1	2	0	0	0	0	0
		156	TGC	0	0	0.582451	2.13813	0	0	0	0	0
157		157	CAT	0	0	0.390699	2.6093	0	0	0	0	0
		158	CGT	0	0	1	2	0	0	0	0	0
		159	TTT	0	0	0.390699	2.6093	0	0	0	0	0
		160	CCG	1	0	1	2	1	0	-1	1	-5.59999
		161	TTT	1	1	0.419618	2.58038	2.3831	0.3875	-1.9956	0.9804	-11.1752
		162	GAA	0.5	1.5	0.425707	2.26755	1.1745	0.6615	-0.513	0.8419	-2.87285
		163	TGG	0	0	0	1.91407	0	0	0	0	0
		164	TCT	1	0	1	2	1	0	-1	1	-5.59999
		165	ATC	0	2	0.869786	2.13021	0	0.9389	0.93887	0.5042	5.25767
		166	CGT	0	0	1	2	0	0	0	0	0
		167	CTG	1	0	1.54319	1.45681	0.648	0	-0.648	1	-3.62884

		168	AAA	0	0	0.397158	2.20753	0	0	0	0	0
		169	GAC	0	1	0.586898	2.4131	0	0.4144	0.4144	0.8044	2.32066
		170	TTC	0	1	0.586891	2.41311	0	0.4144	0.4144	0.8044	2.32065
		171	GAG	1	0	0.42151	2.25311	2.3724	0	-2.3724	1	-13.2855
		172	TAC	0	0	0.582451	2	0	0	0	0	0
		173	ACG	1	0	1	2	1	0	-1	1	-5.59999
		174	GCA	0	0	1	2	0	0	0	0	0
		175	CGC	0	0	1	2	0	0	0	0	0
		176	CAC	1	0	0.581773	2.41823	1.7189	0	-1.7189	1	-9.62573
		177	TTA	1	0	1.44974	1.4485	0.6898	0	-0.6898	1	-3.86276
178		178	TTC	0	0	0.582451	2.41755	0	0	0	0	0
		179	CCA	0	0	1	2	0	0	0	0	0
		180	GAC	0	0	0.582451	2.41755	0	0	0	0	0
		181	CCA	0	0	1	2	0	0	0	0	0
		182	GTT	0	0	1	2	0	0	0	0	0
	183	183	AAC	0	2	0.584838	2.41516	0	0.8281	0.8281	0.6481	4.63736
		184	ACT	1	0	1	2	1	0	-1	1	-5.59999
		185	TAC	0	0	0.582451	2	0	0	0	0	0
		186	ACA	0	1	1	2	0	0.5	0.5	0.6667	2.79999
		187	CCT	0	0	1	2	0	0	0	0	0
		188	GAG	1	0	0.541413	2.13321	1.847	0	-1.847	1	-10.3433
		189	TTT	0	0	0.390699	2.6093	0	0	0	0	0
		190	CCT	1	0	1	2	1	0	-1	1	-5.59999
		191	ATC	0	0	0.861875	2.13813	0	0	0	0	0
		192	GAG	0	0	0.542963	2.13166	0	0	0	0	0

		193	TCC	0	0	1	2	0	0	0	0	0
		194	CTC	0	0	1	2	0	0	0	0	0
		195	ATG	0	1	0.00856171	2.99144	0	0.3343	0.33429	0.9971	1.872
		196	TGT	0	0	0.390699	2.22898	0	0	0	0	0
		197	CAT	0	1	0.397183	2.60282	0	0.3842	0.3842	0.8676	2.15151
		198	GAG	0	0	0.542963	2.13166	0	0	0	0	0
		199	ACC	0	0	1	2	0	0	0	0	0
		200	ATT	0	0	0.771022	2.22898	0	0	0	0	0
		201	GAA	0	0	0.397158	2.27746	0	0	0	0	0
		202	AGC	0	0	0.582451	2.41755	0	0	0	0	0
		203	TGG	0	0	0	1.91407	0	0	0	0	0
		204	AAA	0	0	0.397158	2.20753	0	0	0	0	0
		205	AAT	0	1	0.407036	2.59296	0	0.3857	0.38566	0.8643	2.15969
		206	GAA	0	2	0.396074	2.33315	0	0.8572	0.85721	0.7308	4.80036
		207	CAT	0	0	0.390699	2.6093	0	0	0	0	0
		208	CTA	2	0	1.44363	1.55637	1.3854	0	-1.3854	1	-7.75823
		209	TAC	0	0	0.582451	2	0	0	0	0	0
		210	TCC	0	0	1	2	0	0	0	0	0
		211	GTA	0	0	1	2	0	0	0	0	0
		212	AGC	0	0	0.582451	2.41755	0	0	0	0	0
		213	CTC	0	0	1	2	0	0	0	0	0
		214	GAA	0	0	0.397158	2.27746	0	0	0	0	0
		215	TCA	0	0	1	1.58245	0	0	0	0	0
		216	TAC	1	0	0.581773	2	1.7189	0	-1.7189	1	-9.62573
		217	ATC	0	0	0.861875	2.13813	0	0	0	0	0

		218	CTT	0	0	1	2	0	0	0	0	0
		219	TGT	0	0	0.390699	2.22898	0	0	0	0	0
		220	ACT	0	0	1	2	0	0	0	0	0
		221	TCC	1	0	1	1.96596	1	0	-1	1	-5.59999
		222	ACT	0	0	1	2	0	0	0	0	0
		223	CCT	0	0	1	2	0	0	0	0	0
		224	GCG	0	0	1	2	0	0	0	0	0
		225	GAT	0	0	0.390699	2.6093	0	0	0	0	0
		226	AAG	0	1	0.542963	2.06197	0	0.485	0.48497	0.7916	2.71584
		227	GCC	0	0	1	2	0	0	0	0	0
		228	GTA	1	0	1	2	1	0	-1	1	-5.59999
		229	ACT	0	0	1	2	0	0	0	0	0
		230	GAT	0	0	0.390699	2.6093	0	0	0	0	0
		231	TTA	1	0	0.791475	1.60491	1.2635	0	-1.2635	1	-7.07538
		232	GAG	0	1	0.542963	2.12984	0	0.4695	0.46952	0.7969	2.6293
		233	TGG	0	0	0	1.91407	0	0	0	0	0
		234	ATG	0	0	0	3	0	0	0	0	0
		235	GAC	0	0	0.582451	2.41755	0	0	0	0	0
		236	GAT	0	0	0.390699	2.6093	0	0	0	0	0
		237	TAT	0	0	0.390699	2	0	0	0	0	0
		238	TCC	0	0	1	2	0	0	0	0	0
		239	AGG	0	0	0.750493	2.24951	0	0	0	0	0
240		240	AGT	0	0	0.390699	2.6093	0	0	0	0	0
		241	CAC	0	0	0.582451	2.41755	0	0	0	0	0
		242	CGG	0	0	1.27942	1.72058	0	0	0	0	0

		243	GAC	0	1	0.580478	2.41606	0	0.4139	0.4139	0.8063	2.31782
		244	GGC	0	0	1	2	0	0	0	0	0
		245	ATA	0	0	0.602842	2.39716	0	0	0	0	0
		246	AGT	0	0	0.390699	2.6093	0	0	0	0	0
		247	CCA	1	0	1	2	1	0	-1	1	-5.59999
		248	TCT	0	2	0.99351	2	0	1	1	0.4464	5.59999
		249	ACA	0	0	1	2	0	0	0	0	0
		250	TCT	0	0	1	2	0	0	0	0	0
		251	GCG	0	0	1	2	0	0	0	0	0
		252	GTC	0	0	1	2	0	0	0	0	0
		253	CGA	0	0	1.27942	1.13813	0	0	0	0	0
		254	CAA	0	0	0.397158	2.02039	0	0	0	0	0
		255	GAA	0	0	0.397158	2.27746	0	0	0	0	0
		256	CAG	0	0	0.542963	1.87459	0	0	0	0	0
		257	GAA	0	0	0.397158	2.27746	0	0	0	0	0
		258	AGA	0	1	0.595773	2.00891	0	0.4978	0.49778	0.7713	2.78757
		259	CTT	0	0	1	2	0	0	0	0	0
		260	CCT	0	0	1	2	0	0	0	0	0
		261	GGG	0	0	1	2	0	0	0	0	0
		262	CAA	0	0	0.397158	2.02039	0	0	0	0	0
		263	GAT	0	0	0.390699	2.6093	0	0	0	0	0
		264	CTC	0	0	1	2	0	0	0	0	0

Supplementary Table S 6. The primers used in this study

Primer name	Primer sequence（ 5′→3′）
40F	TGAGTGCGCGGAGGCTTTTGG
806R	TCTGGCATTGCCTGTTTCGG
735F	TCCGTTCATCGGTCCAGTCCG
1886R	ACTCCGTAAGCCTCGAATCC
1828F	TTGCGCTTGATCCGCAGGAG
118R	AAGCTAAGGCATGGCACACATTTCC
